# Synthesis and Characterization of Pyridine Dipyrrolide Uranyl Complexes

**DOI:** 10.1021/acs.inorgchem.2c00348

**Published:** 2022-04-14

**Authors:** Brett M. Hakey, Dylan C. Leary, Lauren M. Lopez, Leyla R. Valerio, William W. Brennessel, Carsten Milsmann, Ellen M. Matson

**Affiliations:** † Department of Chemistry, University of Rochester, Rochester, New York 14627, United States; ‡ C. Eugene Bennett Department of Chemistry, West Virginia University, Morgantown, West Virginia 26506, United States

## Abstract

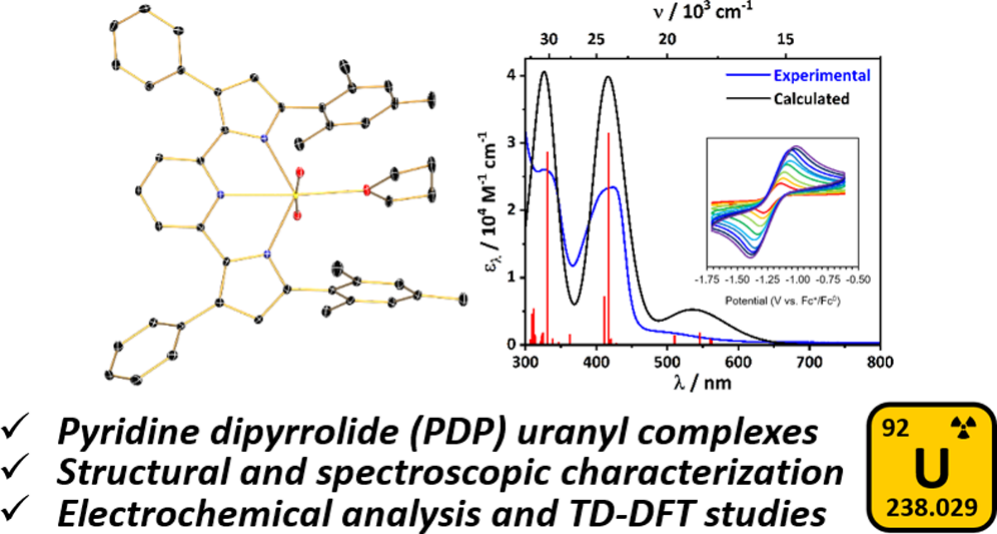

The first actinide complexes of the pyridine dipyrrolide (PDP) ligand class, (^Mes^PDP^Ph^)UO_2_(THF) and (^Cl2Ph^PDP^Ph^)UO_2_(THF), are reported as the U^VI^ uranyl adducts of the bulky aryl substituted pincers (^Mes^PDP^Ph^)^2–^ and (^Cl2Ph^PDP^Ph^)^2–^ (derived from 2,6-bis(5-(2,4,6-trimethylphenyl)-3-phenyl-1*H*-pyrrol-2-yl)pyridine (H_2_^Mes^PDP^Ph^, Mes = 2,4,6-trimethylphenyl), and 2,6-bis(5-(2,6-dichlorophenyl)-3-phenyl-1*H*-pyrrol-2-yl)pyridine (H_2_^Cl2Ph^PDP^Ph^, Cl_2_Ph = 2,6-dichlorophenyl), respectively). Following the in situ deprotonation of the proligand with lithium hexamethyldisilazide to generate the corresponding dilithium salts (e.g., Li_2_^Ar^PDP^Ph^, Ar = Mes of Cl_2_Ph), salt metathesis with [UO_2_Cl_2_(THF)_2_]_2_ afforded both compounds in moderate yields. The characterization of each species has been undertaken by a combination of solid- and solution-state methods, including combustion analysis, infrared, electronic absorption, and NMR spectroscopies. In both complexes, single-crystal X-ray diffraction has revealed a distorted octahedral geometry in the solid state, enforced by the bite angle of the rigid meridional (^Ar^PDP^Ph^)^2–^ pincer ligand. The electrochemical analysis of both compounds by cyclic voltammetry in tetrahydrofuran (THF) reveals rich redox profiles, including events assigned as U^VI^/U^V^ redox couples. A time-dependent density functional theory study has been performed on (^Mes^PDP^Ph^)UO_2_(THF) and provides insight into the nature of the transitions that comprise its electronic absorption spectrum.

## Abstract 2

The synthesis of two uranyl adducts of the pyridine dipyrrolide ligand class is reported. The characterization of these complexes by cyclic voltammetry and electronic absorption spectroscopy reveals intriguing electronic profiles.

## Introduction

For decades, synthetic chemists have devoted attention to the study of the coordination chemistry of the most ubiquitous form of uranium, the uranyl dication, [UO_2_]^2+^.^1,2^ Motivating factors for this research include furthering the fundamental understanding of the chemistry of this element, which has an important relationship to the nuclear fuel cycle.^1^ Interest in the reactivity of [UO_2_]^2+^ is also rooted in the need to develop strategies to combat ecological and anthropogenic contamination streams of this mobile form of uranium.^3^ Consequently, a common research theme that has materialized in this area is the pursuit of chemical agents capable of uranyl sequestration, and thus, scores of uranyl complexes featuring polydentate chelating ligands have been synthesized and structurally characterized.^1,4−6^

**Figure 1:**
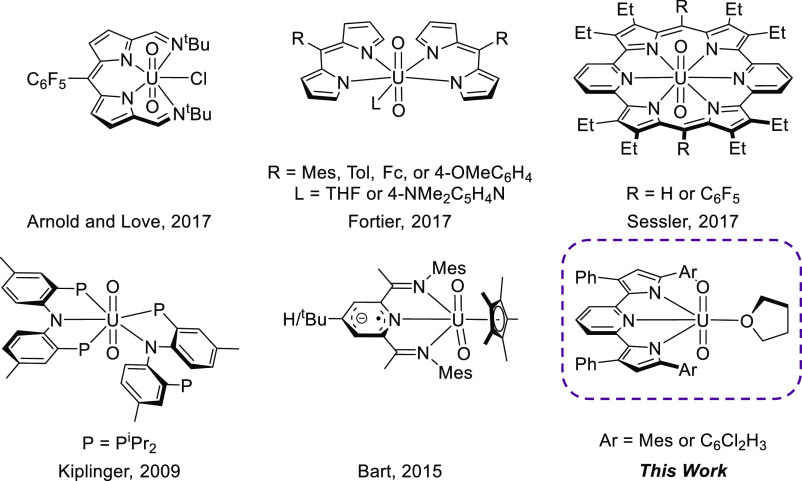
Selected uranyl complexes featuring pyrrole-based or anionic nitrogen-containing pincer ligands.

In recent years, the renewed interest in the nonaqueous chemistry of the actinides has spurred the investigation of the uranyl coordination chemistry of an increasingly diverse array of ligand classes, including pyrrole-derived frameworks.^2^ In particular, polypyrrolic macrocycles have proven quite successful in actinyl coordination chemistry (Figure 1). Following early contributions by Marks,^7,8^ the Sessler group has reported numerous innovations in this space.^9−24^ Notable developments include uranyl and neptunyl complexes of the expanded porphyrin hexaphyrin(1.0.1.0.0.0) and a bench-stable uranyl dipyriamethyrin complex. Moreover, in seminal contributions, Arnold and Love have demonstrated that polypyrrolic “Pacman” ligands are suitable platforms for stabilizing transition metal uranyl adducts^25^ and conducting uranyl silylation.^26^ Additional reports from these researchers have further elaborated on this chemistry^27−44^ and extended the scope of suitable supporting ligands to tetradentate dipyrrins.^45−47^ In complementary work, Fortier has disclosed emissive seven-coordinate uranyl complexes carrying two redox-active dipyrrinate ligands.^48^

Surprisingly, despite the vast number of structurally characterized compounds, the coordination chemistry of uranyl complexes supported by anionic pincer ligands has only recently emerged (Figure 1).^49^ Important early contributions in the area of anionic pincer uranyl chemistry arose from the study of bis-(thiophosphinoyl)methane type ligands, including the first uranyl carbene complex, [UO_2_(SCS)(py)_2_] (SCS = bis-(thiophosphinoyl)methane dianion and py = pyridine) described by Ephritikhine in 2011.^70^ Kiplinger and co-workers have also reported that pincers are capable of supporting structures for actinides that are unattainable using traditional carbocyclic ligands, such as Cp* (Cp* = 1,2,3,4,5-pentamethylcyclopentadienide), as demonstrated by the synthesis and reactivity studies of a PNP pincer uranyl species.^51^ Moreover, Bart and co-workers have extended uranyl chemistry to the tridentate redox-active pyridine diimine (PDI) and dioxophenoxazine frameworks.^52,53^ In the case of PDI, it was discovered that reducing equivalents stored on the chelate ligand promote the reductive silylation of the uranyl moiety.

Of late, a new family of tridentate pincer ligands, so called pyridine dipyrrolides (PDPs) in their dianionic, doubly deprotonated form (PDP^2–^), has garnered widespread attention in both transition metal and main group chemistry.^54−73^ Because H_2_PDP proligands are prepared in a modular fashion, the corresponding PDP^2–^ ligands are well suited for the manipulation of specific steric and electronic profiles of coordinated metals. With this in mind, researchers have leveraged PDP metal complexes in catalysis,^50^ atom and group transfer,^56,57,61^ and photochemistry.^64−68^ Despite this expanding wealth of research, PDP compounds of either lanthanide or actinide elements have not been reported.^74^

Accordingly, our research groups have endeavored to initiate the study of actinide-PDP compounds with the aim of establishing the fundamental coordination chemistry of these systems by utilizing the diamagnetic, largely inert, uranyl moiety as a convenient entry point. Interested in the synthesis of uranyl compounds as a means to gain entry to actinide PDP chemistry, we anticipated that the previously reported bulky ligands (^Ar^PDP^Ph^)^2–^ (Ar = Mes or Cl_2_Ph), (^Mes^PDP^Ph^)^2–^ and (^Cl2Ph^PDP^Ph^)^2–^, derived from the double deprotonation of 2,6-bis(5-(2,4,6-trimethylphenyl)-3-phenyl-1*H*-pyrrol-2-yl)pyridine (H_2_^Mes^PDP^Ph^, Mes = 2,4,6-trimethylphenyl) and 2,6-bis(5-(2,6-dichlorophenyl)-3-phenyl-1H-pyrrol-2-yl)pyridine (H_2_^Cl2Ph^PDP^Ph^, Cl_2_Ph = 2,6-dichlorophenyl), may support compounds of the general formula (^Ar^PDP^Ph^)UO_2_(L) (where L = a neutral donor ligand). These ligand precursors were attractive choices, as each may be synthesized in a modular fashion on gram scales without the need for chromatographic separations. Additionally, the rigid coordination environments imparted by these ligand platforms were anticipated to yield well-defined, symmetric compounds amenable to straightforward study by solution NMR spectroscopy. Interestingly, despite the steric similarities of these compounds, the coordination chemistry of this pair of ligands has been observed to have clear differences. For example, some of us have recently reported that the (^Cl2Ph^PDP^Ph^)^2–^ ligand engages in weak chloride-to-iron interactions in the solid state in iron compounds in both the +II and +IV formal oxidation states.^62^

Herein, we describe a two-step, one-pot metalation protocol for the synthesis of a series of PDP uranyl complexes, namely (^Mes^PDP^Ph^)UO_2_(THF) and (^Cl2Ph^PDP^Ph^)UO_2_(THF) (Figure 1). These complexes have been characterized via a combination of solid-state and solution-phase techniques, including single-crystal X-ray diffraction (SCXRD), infrared (IR) spectroscopy, combustion analysis, ^1^H and ^13^C{^1^H} NMR, electronic absorption spectroscopy, and cyclic voltammetry (CV). These efforts have demonstrated that tridentate (^Ar^PDP^Ph^)^2–^ ligands support rigid six-coordinate, distorted octahedral uranyl complexes both in the solid and solution state. CV studies demonstrate rich electrochemistry for both compounds, suggesting that the charged analogues may be chemically accessible. A complementary time-dependent density functional theory (TD-DFT) study has been conducted as a means of further elucidating the nature of the electronic transitions for (^Mes^PDP^Ph^)UO_2_(THF). These calculations have revealed that the nature of the dominant transitions in the visible region of the electronic absorption spectrum are primarily a result of intraligand charge transfer (ILCT) with some ligand-to-metal charge transfer (LMCT) contributions. We anticipate that the results presented here will serve as a foundation for future exploration in PDP f-element chemistry.

## Experimental Section

### General Considerations

All air- and moisture-sensitive manipulations were carried out using a standard high vacuum line, Schlenk, or cannula techniques or in an MBraun inert atmosphere drybox containing an atmosphere of purified dinitrogen. All solids were dried under a high vacuum in order to be brought into the glovebox. Solvents for air- and moisture-sensitive manipulations were dried and deoxygenated using a Glass Contour Solvent Purification System (Pure Process Technology, LLC) and stored over activated 4 Å molecular sieves (Fisher Scientific) prior to use. Deuterated solvents for NMR spectroscopy were purchased from Cambridge Isotope Laboratories, distilled from sodium metal (C_6_D_6_) or CaH_2_ (CD_2_Cl_2_) after three freeze–pump–thaw cycles, and stored in the glovebox over activated 3 Å molecular sieves. Uranyl chloride trihydrate was purchased from International Bio-Analytical Industries and used as received. All the remaining chemicals were purchased from commercial sources (Fisher Scientific, VWR, and Sigma-Aldrich) and used without further purification. H_2_^Mes^PDP^Ph^,^60^ H_2_^Cl2Ph^PDP^Ph^,^62^ and [UO_2_Cl_2_(THF)_2_]_2_^75^ were synthesized following reported procedures.

### Safety Considerations

Caution! Depleted uranium (primary isotope ^238^U) is a weak α-emitter (4.197 MeV) with a half-life of 4.47 × 10^9^ years; manipulations and reactions should be carried out in monitored fume hoods or in an inert atmosphere drybox in a radiation laboratory equipped with α- and β-counting equipment.

### Synthesis of (^Mes^PDP^Ph^)UO_2_(THF)

In the glovebox, a 20 mL scintillation vial equipped with a magnetic stirrer was loaded with H_2_^Mes^PDP^Ph^ (0.250 g, 0.418 mmol, 1.0 equiv) and 3 mL of diethyl ether, affording a yellow suspension. In a separate vial, lithium hexamethyldisilazide (LiHMDS; 143 mg, 0.855 mmol, 2.05 equiv) was dissolved in approximately 3 mL of diethyl ether. The LiHMDS solution was added dropwise to the suspension of H_2_^Mes^PDP^Ph^ with vigorous stirring, inducing an immediate color change to a brilliant yellow. Within minutes, complete dissolution of all the solids was observed; stirring was continued for approximately 2 h. In a separate vial, [UO_2_Cl_2_(THF)_2_]_2_ (0.203 g, 0.209 mmol, 0.5 equiv) was suspended in 3 mL of diethyl ether and added dropwise to the solution of Li_2_^Mes^PDP^Ph^, inducing an immediate color change to dark-red/brown. The mixture was stirred for 12 h, after which the suspension was filtered over a 1″ pad of Celite in a glass pipette plugged with a microfiber glass filter. A brown powder was collected on the Celite column and washed three times with 2 mL aliquots of pentane. The material was then extracted from the Celite plug using 10 mL of dichloromethane (DCM). The red/brown extracts were collected in a tared, 20 mL scintillation vial and volatiles were removed in vacuo. The residue was then extracted into benzene and passed through an additional Celite plug, collected in a tared, 20 mL scintillation vial, and reduced to dryness. The resulting dark residue was triturated with pentane, leaving a brick-red powder that was identified as the title compound. Yield: 0.209 g, 0.223 mmol, 53%. ^1^H NMR (400 MHz, C_6_D_6_): δ 7.83 (d, *J* = 7.1 Hz, 4H, *o*-Ph*H*), 7.57 (d, *J* = 8.0 Hz, 2H, 3-pyridine*H*), 7.29 (t, *J* = 7.6 Hz, 4H, *m*-Ph*H*), 7.18–7.14 (m, 2H, *p*-Ph*H*, overlapping w/residual benzene), 6.76 (m, 5H, 4-pyridine*H* and *m*-MesC*H*), 6.52 (s, 2H, pyrrole*H*), 3.84–3.59 (m, 4H, THF-α-C*H*_2_), 2.39 (s, 12H, *o*-MesC*H*_3_), 2.14 (s, 6H, *p*-MesC*H*_3_), 1.41–1.38 (m, 4H, THF-β-C*H*_2_). ^13^C NMR (126 MHz, C_6_D_6_): δ 20.99, 21.21, 26.27, 76.88, 114.03, 114.35, 126.58, 128.63, 128.70, 130.66, 131.31, 133.02, 137.42, 138.58, 138.89, 139.54, 140.44, 141.14, 158.05. The red crystals of (^Mes^PDP^Ph^)UO_2_(THF) suitable for SCXRD were grown from a mixture of toluene and pentane at −30 °C. Anal. Calcd for C_47_H_45_N_3_O_3_U (mol. wt. 937.924 g/mol): C, 60.19; H, 4.84; N, 4.48. Found: C, 60.79; H, 4.82; N, 4.38.

### Synthesis of (^Cl2Ph^PDP^Ph^)UO_2_(THF)

In the glovebox, a 20 mL scintillation vial equipped with a magnetic stirrer was loaded with H_2_^Cl2Ph^PDP^Ph^ (0.200 g, 0.307 mmol, 1.0 equiv) and 2 mL of diethyl ether, affording a clear, homogeneous solution. In a separate vial, LiHMDS (0.105 g, 0.630 mmol, 2.05 equiv) was dissolved in approximately 2 mL of diethyl ether. The LiHMDS solution was added to the solution of H_2_^Cl2Ph^PDP^Ph^ with vigorous stirring, inducing an immediate color change to a brilliant luminescent yellow, accompanied by the dissolution of all the solids. The resulting solution was stirred for approximately 2 h. In a separate vial, [UO_2_Cl_2_(THF)_2_]_2_ (0.149 g, 0.154 mmol, 0.5 equiv) was suspended in 3 mL of diethyl ether and added dropwise to the suspension of Li_2_^Cl2Ph^PDP^Ph^, inducing an immediate color change to dark-red/brown. The mixture was stirred for 16 h, at which time the resulting suspension was filtered over a 1″ pad of Celite in a glass pipette plugged with a microfiber glass filter. A brown powder was collected on the Celite column and washed with three 2 mL aliquots of pentane. The material was then extracted from the Celite plug using 10 mL of DCM. The red-brown extracts were collected and volatiles were removed in vacuo. The residue was then extracted into benzene and passed through an additional Celite plug, collected in a tared, 20 mL scintillation vial, and reduced to dryness. The resulting dark residue was triturated with pentane, leaving a brown powder that was identified as the title compound. Yield: 0.190 g, 0.192 mmol, 62%. Red, single crystals of (^Cl2Ph^PDP^Ph^)UO_2_(THF) suitable for SCXRD were grown from the diffusion of pentane into a toluene solution of the compound. ^1^H NMR (400 MHz, C_6_D_6_): δ 7.76 (d, *J* = 7.3 Hz, 4H, *o*-Ph*H*), 7.46 (d, *J* = 8.0 Hz, 2H, 3-pyridine*H*), 7.26 (t, *J* = 7.6 Hz, 4H, *m*-Ph*H*), 7.15–7.13 (m, 2H, *p*-Ph*H*, overlapping w/residual benzene), 6.99 (d, *J* = 8.1 Hz, 4H, *m*-Cl_2_Ph*H*), 6.91 (s, 2H, pyrrole*H*), 6.64 (t, *J* = 8.0 Hz, 1H, 4-pyridine*H*), 6.46 (t, *J* = 8.1 Hz, 2H, *p*- Cl_2_Ph*H*), 4.04 (m, 4H, THF-α-C*H*_2_), 1.45 (m, 4H, THF-β-C*H*_2_). ^13^C NMR (126 MHz, C_6_D_6_): δ 26.60, 77.40, 115.17, 117.03, 126.81, 128.33, 128.59, 128.74, 130.72, 131.20, 136.90, 137.35, 138.89, 138.94, 139.26, 156.66. One resonance was not detected. Anal. Calcd for C_41_H_29_Cl_4_N_3_O_3_U (mol. wt. 991.530 g/mol): C, 49.67; H, 2.95; N, 4.24. Found: C, 49.81; H, 2.80; N, 4.12.

### Physical Measurements

^1^H and ^13^C{^1^H} NMR spectra were recorded at room temperature on a 400 MHz Bruker AVANCE spectrometer or a 500 MHz Bruker AVANCE spectrometer locked on the signal of deuterated solvents. All the chemical shifts are reported relative to SiMe_4_ using ^1^H (residual) chemical shifts of the solvent as a secondary standard. The Fourier transform IR (FT-IR) spectra of compounds were recorded on a Shimadzu IRAffinity-1 FT-IR spectrophotometer and are reported in wavenumbers (cm^–1^). Electronic absorption measurements were recorded at room temperature in anhydrous tetrahydrofuran (THF) in sealed 1 cm quartz cuvettes using an Agilent Cary 60 UV–Vis spectrophotometer. CV experiments were performed at room temperature in an MBraun inert atmosphere drybox containing an atmosphere of purified nitrogen using a Bio-Logic SP200 potentiostat/galvanostat and the EC-Lab software suite. A three-electrode system cell configuration that consisted of a glassy carbon (ø = 3.0 mm) as the working electrode (CH Instruments, USA), a platinum wire as the counter electrode (CH Instruments, USA), and a silver wire as the quasi-reference electrode was employed. All the CV measurements utilized 1 mM sample solutions in THF or DCM with 0.1 M tetrabutylammonium hexafluorophosphate as the supporting electrolyte. Ferrocene (Fc) was added as an internal standard after the completion of the measurements, and all potentials were referenced versus the Fc^+^/Fc^0^ couple. Elemental analysis data were obtained from the Elemental Analysis Facility at the University of Rochester. Microanalysis samples were weighed with a PerkinElmer model AD6000 Autobalance and their compositions were determined with a PerkinElmer 2400 Series II analyzer. Air-sensitive samples were handled in a VAC Atmospheres glovebox.

### X-ray Crystallography

The single crystals of complexes **(**^**Mes**^**PDP**^**Ph**^**)UO**_**2**_**(THF)** and **(**^**Cl2Ph**^**PDP**^**Ph**^**)UO**_**2**_**(THF)** were mounted on a thin glass optical fiber or a nylon loop and mounted on a Rigaku XtaLAB Synergy-S Dualflex diffractometer equipped with a HyPix-6000HE HPC area detector for data collection at 100.00(10) K. A preliminary set of cell constants and an orientation matrix were calculated from a small sampling of reflections.^76^ A short pre-experiment was run, from which an optimal data collection strategy was determined. In the case of complex **(**^**Mes**^**PDP**^**Ph**^**)UO**_**2**_**(THF)**, the full data collection was carried out using a PhotonJet (Cu) X-ray source, whereas data collection for **(**^**Cl2Ph**^**PDP**^**Ph**^**)UO**_**2**_**(THF)** was performed using a PhotonJet (Mo) X-ray source. After the intensity data were corrected for absorption, the final cell constants were calculated from the xyz centroids of the strong reflections from the actual data collections after integration.^76^ See the Supporting Information file for additional crystal and refinement information. The structure was solved using SHELXT^77^ and refined using SHELXL.^78^ Most or all non-hydrogen atoms were assigned from the solution. Full-matrix least squares/difference Fourier cycles were performed, which located any remaining non-hydrogen atoms. All the non-hydrogen atoms were refined with anisotropic displacement parameters. All the hydrogen atoms were placed in ideal positions and refined as riding atoms with relative isotropic displacement parameters.

### Computational Methods

All the calculations were performed using the ORCA quantum chemical program package v5.0.1.^79,80^ Geometry optimizations used the PBE functional^81^ and were accelerated using the resolution of identity (RI) approximation.^82,83^ Scalar-relativistic effects were included *via* the zeroth-order regular approximation (ZORA)^84^ using relativistically recontracted triple-ζ quality basis sets, ZORA-def2-TZVP^85^ on nitrogen and oxygen atoms and SARC-ZORA-TZVP^86^ for uranium. All the other atoms were handled with the recontracted split-valence ZORA-def2-SVP basis set.^85^ Noncovalent interactions were considered via atom-pairwise dispersion corrections with Becke–Johnson (D3BJ) damping.^87,88^ The TD-DFT calculations used the B3LYP density functional^89^ and were accelerated using the RIJCOSX approximation.^90,91^ Relativistic effects were included using the Douglas–Kroll–Hess (DKH) Hamiltonian with DKH-specific basis sets analogous to those used in the geometry optimizations. The Tamm–Dancoff approximation was not used, and the effects of spin–orbit coupling (SOC) were probed using a spin–orbit mean field (SOMF) approach.^92^ All the solvation effects were handled using the conductor-like polarizable continuum model (C-PCM) and a Gaussian charge scheme.^93^

## Results and Discussion

**Scheme 1:**
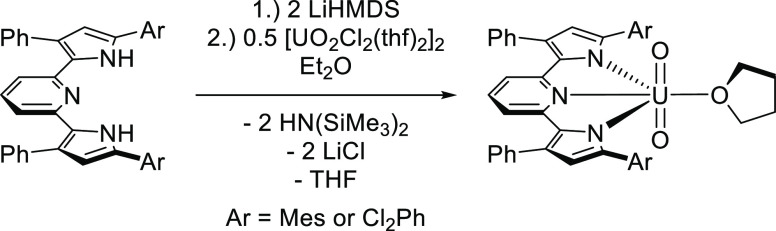
Synthesis of (^Ar^PDP^Ph^)UO_2_(THF) Complexes

Following literature protocols,^60−62^ the in situ preparation of the reported PDP dilithium salts Li_2_^Ar^PDP^Ph^ of the corresponding pyridine dipyrrole compounds H_2_^Ar^PDP^Ph^ was chosen as a convenient synthetic access point to metathesis chemistry with the popular nonaqueous uranyl starting material [UO_2_Cl_2_(THF)_2_]_2_.^75^ Upon the addition of a diethyl ether slurry of [UO_2_Cl_2_(THF)_2_]_2_ to a slurry of in situ prepared Li_2_^Mes^PDP^Ph^ in the same solvent, an immediate color change from a luminescent yellow to a dark-red/brown was observed. This color change was accompanied by the dissolution of all the solids. The reaction mixture was stirred overnight at room temperature, at which time the formation of a brown precipitate was evident. Following filtration and workup (see Experimental Section for details), a brick-red powder identified as (^Mes^PDP^Ph^)UO_2_(THF) was obtained in 53% yield (Scheme 1, *vide infra*). Furthermore, it was determined that (^Mes^PDP^Ph^)UO_2_(THF) was amenable to additional purification by means of recrystallization (vide infra) by the diffusion of pentane into a saturated toluene solution of the compound.

The initial characterization of (^Mes^PDP^Ph^)UO_2_(THF) was accomplished by IR spectroscopy (Figure S9). A spectrum obtained from a bulk powder sample of the compound revealed no indication of pyrrole N–H stretches, consistent with successful deprotonation and metalation of both pyrrolide nitrogens. A prominent band was identified at a frequency of 917 cm^–1^, attributed to the asymmetric (ν_asym_) O=U=O stretch of (^Mes^PDP^Ph^)UO_2_(THF).^94^ This value is in excellent agreement with that of a macrocylic dipyrrolide tetraimine uranyl complex reported by Sessler and co-workers, characterized by a ν_asym_ O=U=O stretch at 910 cm^–1^.^10^

**Figure 2:**
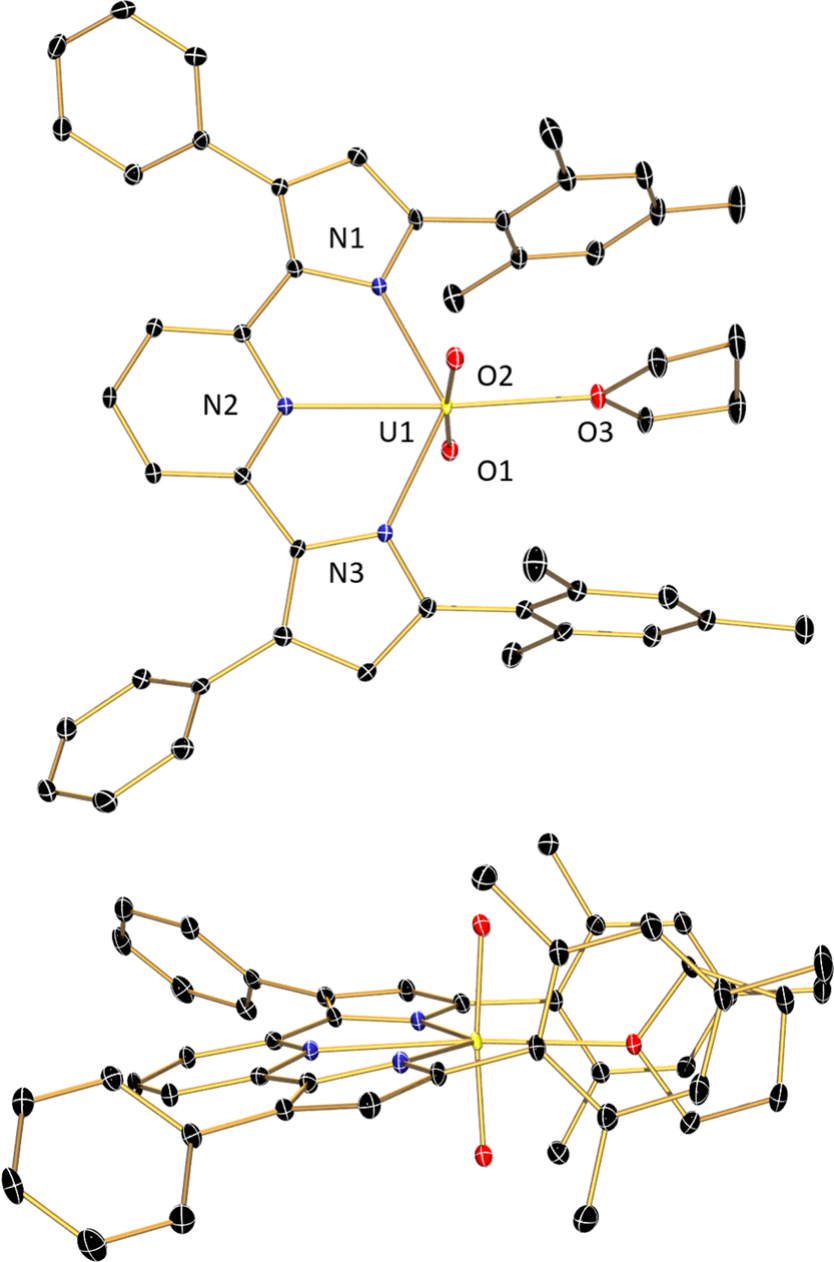
Top: Molecular structure of (^Mes^PDP^Ph^)UO_2_(THF) (100 K, λ = 1.54184 Å) viewed from above. Bottom: Side view of (^Mes^PDP^Ph^)UO_2_(THF) demonstrating PDP ligand folding. Both structures are depicted with 30% probability ellipsoids. All hydrogen atoms are omitted for clarity.

Furthermore, spectroscopic characterization of (^Mes^PDP^Ph^)UO_2_(THF) was also performed by means of ^1^H and ^13^C{^1^H} NMR spectroscopy at room temperature in benzene-*d*_6_ (C_6_D_6_). Both the spectra revealed the expected number of resonances with relative integrations (^1^H NMR), consistent with a *C*_2*v*_ symmetric structure in the solution (Figures S1–S3). Moreover, closer inspection revealed a diagnostic singlet resonance at 6.52 ppm, corresponding to the 4-pyrrolide hydrogens of (^Mes^PDP^Ph^)UO_2_(THF). The 4-pyridyl proton, also routinely used as a convenient spectroscopic handle for PDP compounds, was located at 7.14 ppm, overlapping with a singlet signal attributed to the *meta* protons of the 5-pyrrolide mesityl substituent. Resonances assigned to the two methylene groups of a bound THF ligand were identified at 4.01 and 1.98 ppm, respectively. The integrations of these two signals were consistent with a 1:1 PDP ligand to THF ratio, which agrees with the proposed assignment as a monosolvated uranyl complex. The ^1^H NMR spectra obtained from the samples of (^Mes^PDP^Ph^)UO_2_(THF) following prolonged exposure to dynamic vacuum did not reveal any changes in the relative integration of the resonances corresponding to the coordinated THF ligand, consistent with its retention under these conditions. However, (^Mes^PDP^Ph^)UO_2_(THF) was determined to engage in facile ligand substitution with stronger Lewis bases. For example, the addition of 1 equiv of 4-dimethylaminopyridine (DMAP) to a benzene-*d*_6_ solution of (^Mes^PDP^Ph^)UO_2_(THF) was found to readily displace the coordinated THF ligand, resulting in the formation of the corresponding DMAP adduct, (^Mes^PDP^Ph^)UO_2_(DMAP), as ascertained by ^1^H NMR spectroscopy (Figure S8).

The additional solid-state characterization of (^Mes^PDP^Ph^)UO_2_(THF) was obtained by SCXRD (Figure 2, Table 1). The recrystallization of the bulk material (toluene and pentane, −30 °C) yielded orange needle-like single crystals of (^Mes^PDP^Ph^)UO_2_(THF) suitable for analysis. The SCXRD study (100.00(10) K) confirmed the identity of the compound as the six-coordinate species (^Mes^PDP^Ph^)UO_2_(THF), which had crystallized in the monoclinic space group *I*2/*a*. An ORTEP representation of the molecular structure demonstrates that the solid-state structure is consistent with the apparent *C*_2*v*_ point group symmetry inferred from the solution phase ^1^H and ^13^C{^1^H} NMR spectroscopic studies. The equatorial positions of the distorted octahedral compound are defined by the meridional N_3_ (^Mes^PDP^Ph^) chelate and THF ligands, of which, the equatorial bond angles sum to 360.09°. Moreover, due to the bite angle enforced by the rigid ^Mes^PDP^Ph2–^ ligand, which routinely enforces N1–M–N3 bond angles of less than 140°, a significant deviation from an idealized octahedral geometry is noted for (^Mes^PDP^Ph^)UO_2_(THF), which contains an N1–U–N3 bond angle of 129.05(8)°. Additionally, a deviation from planarity arises from the pyridine of the PDP chelate folding below the plane established by the remainder of the PDP chelate (Figure 2), as demonstrated by the N2–U–O3 bond angle of 175.38(7)°.

**Figure 3:**
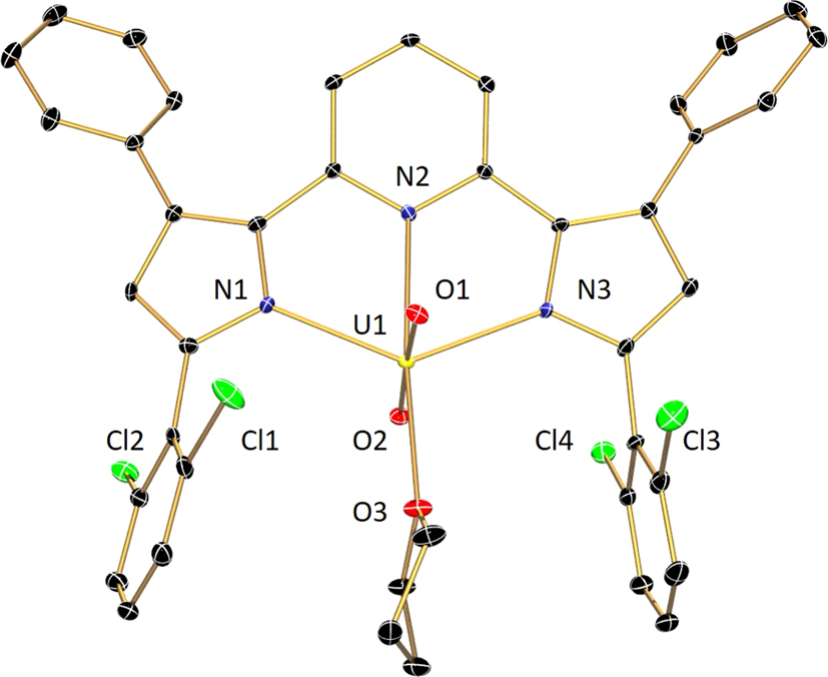
Molecular structure of (^Cl2Ph^PDP^Ph^)UO_2_(THF)·C_7_H_8_ (100 K, λ = 0.71073 Å) depicted with 30% probability ellipsoids. Hydrogen atoms and the toluene solvent molecule are omitted for clarity.

**Table 1:** Selected Bond Lengths (Å) and Angles (deg) for (^Mes^PDP^Ph^)UO_2_(THF) and (^Cl2Ph^PDP^Ph^)UO_2_(THF)

	(^Mes^PDP^Ph^)UO_2_(THF)	(^Cl2Ph^PDP^Ph^)UO_2_(THF)
U1–O1	1.773(2)	1.771(3)
U1–O2	1.774(2)	1.771(2)
U1–O3	2.3917(19)	2.408(2)
U1–N1	2.354(2)	2.381(3)
U1–N2	2.535(2)	2.504(3)
U1–N3	2.356(2)	2.367(3)
U1–Cl1		3.503
N2–U1–O3	175.38(7)	174.76(10)
N1–U1–N3	129.05(8)	130.61(9)
O1–U1–O2	173.94(9)	174.67(11)

The six-coordinate geometry of (^Mes^PDP^Ph^)UO_2_(THF) is noteworthy, as often the large ionic radius of uranium results in uranyl compounds with coordination numbers of seven, for mononuclear species. This holds true for most pincer uranyl complexes,^50,51^ with the PDI uranyl complexes, Cp*UO_2_(^Mes^PDI^Me^) and Cp*UO_2_(^t^Bu-^Mes^PDI^Me^) (^Mes^PDI^Me^ = 2,6-((Mes)N=CMe)_2_C_5_H_3_N; ^t^Bu-^Mes^PDI^Me^ = 2,6-((Mes)–N=CMe)_2_-*p*-C(CH_3_)_3_C_5_H_2_N; Me = methyl), reported by Bart and co-workers being notable exceptions.^52^ In these instances, the six-coordinate geometry results as a function of a bulky Cp* ligand, positioned *trans* to the PDI pyridine nitrogen and approximately orthogonal to the plane of the chelate ligand. Such an orientation presumably renders a coordination number higher than six unlikely for this class of compounds. Similarly, the steric bulk of the flanking mesityl groups present in (^Mes^PDP^Ph^)UO_2_(THF) prohibits access of an additional THF solvent ligand within the plane of the PDP chelate, disallowing the possibility of a seven-coordinate species [see Figure S20 for a space-filling representation of (^Mes^PDP^Ph^)UO_2_(THF)].

Other important structural metrics for (^Mes^PDP^Ph^)UO_2_(THF) include uranium-oxo (U-O_yl_) bond lengths of 1.773(2) and 1.774(2) Å for U(1)–O(1) and U(1)–O(2), respectively. These distances are typical for U^VI^ coordination complexes.^95^ For example, the uranyl PNP pincer complex, (PNP)_2_UO_2_ reported by Kiplinger and co-workers has U–O_yl_ bond lengths of 1.791(6) and 1.808(6) Å.^31^ Similar U–O_yl_ bond lengths (1.7792(12) and 1.7796(12) Å) have been reported previously by Liddle and co-workers for the pincer type uranyl complex, [U(BIPM^Mes^H)O_2_(Cl) (THF)].^96^ In the case of (^Mes^PDP^Ph^)UO_2_(THF) the oxo ligands are trans to one another, with an O(1)-U(1)-O(2) bond angle slightly deviated from linearity (173.94(9)°). The U(1)–N(2) (U-N_pyridine_) and U(1)–O(3) (U-O_THF_) bond distances for (^Mes^PDP^Ph^)UO_2_(THF) are 2.535(2) and 2.3917(19) Å, respectively. For comparison, the U–N_pyridine_ bond distance is shorter than that of the pyridine-2,6-dicarboxamide complex uranyl complex UO_2_Cl_2_L^97^ (L = *N*,*N*,*N*′,*N*′-tetraalkylpyridine-2,6-dicarboxamide) possessing (U–N_pyridine_ 2.634(2) Å); however, the U–O_THF_ distance is in good agreement with the average U–O_THF_ bond distance found in [UO_2_Cl_2_(THF)_2_]_2_ (2.40(3) Å).^98^ Completing the primary coordination sphere, the (^Mes^PDP^Ph^)UO_2_(THF) U–N_pyrrole_ contacts, U(1)–N(1) and U(1)–N(3), were determined to be 2.354(2) and 2.356(2) Å, which are shorter than in dipyriamethyrin and dipyrrinate analogues.^19,48^ It is proposed that the shorter U–N_pyridine_ and U–N_pyrrole_ contacts may occur as a result of a combination of steric and electronic effects, including a size matching between the coordination environment established by the (^Mes^PDP^Ph^)^2–^ ligand and the uranyl moiety and the pi-donor character of the pyrrolide nitrogens.

With (^Mes^PDP^Ph^)UO_2_(THF) in hand, we next targeted the preparation of the (^Cl2Ph^PDP^Ph^)UO_2_(THF) analogue by the extension of the same general synthetic protocol (Scheme 1). The addition of a diethyl ether slurry of [UO_2_Cl_2_(THF)_2_]_2_ to a slurry of in situ prepared Li_2_^Cl2Ph^PDP^Ph^ in the same solvent, followed by filtration and workup, provided (^Cl2Ph^PDP^Ph^)UO_2_(THF) in a 62% isolated yield as a polycrystalline brown powder. A solid-state IR spectrum acquired on a bulk powder sample indicated no pyrrole N–H stretching modes, consistent with metalation, and a ν_asym_ O=U=O stretch located at 930 cm^–1^, blue shifted relative to the same feature for (^Mes^PDP^Ph^)UO_2_(THF) (Figure S10). ^1^H and ^13^C{^1^H} NMR spectroscopic analysis of (^ClPh2^PDP^Ph^)UO_2_(THF) in benzene-*d*_6_ at room temperature revealed the expected number of resonances with relative integrations consistent with a *C*_2*v*_ symmetric structure in the solution (Figures S4–S6). This result is consistent with either (1) a solution-phase structure where the *ortho*-chloride substituents interact with the uranium center but rapidly interconvert on the timescale of the NMR experiment, or (2) the *ortho*-chloride substituents do not interact with the uranium center.^62^ A diagnostic singlet resonance assigned to the 4-pyrrolide hydrogens of (^Cl2Ph^PDP^Ph^)UO_2_(THF) was identified at 6.91 ppm, shifted significantly downfield relative to the same protons in the mesityl analogue. A triplet resonance located at 6.64 ppm in the spectrum of (^Cl2Ph^PDP^Ph^)UO_2_(THF) was assigned to the 4-pyridyl proton, upfield relative to the same proton in (^Mes^PDP^Ph^)UO_2_(THF). Resonances corresponding to a bound THF ligand were located at 1.45 and 4.04 ppm, respectively, with integrations consistent with a 1:1 THF/^Cl2^PDP^Ph^ ligand ratio.

The red single crystals of (^Cl2Ph^PDP^Ph^)UO_2_(THF) were obtained from the diffusion of pentane into a concentrated toluene solution of the compound (Figure 3, Table 1). Importantly, there are multiple differences between the solid-state structures of (^Cl2Ph^PDP^Ph^)UO_2_(THF) and (^Mes^PDP^Ph^)UO_2_(THF). First, despite similar crystallization conditions for the two compounds (the diffusion of pentane into a concentrated toluene solution), (^Cl2Ph^PDP^Ph^)UO_2_(THF) crystallizes as a toluene solvate in the monoclinic space group *P*2_1_/*n*. Second, one of the PDP Cl_2_Ph groups in (^Cl2Ph^PDP^Ph^)UO_2_(THF) is canted, whereas the second Cl_2_Ph group is approximately perpendicular to the chelate plane, similar to both mesityl substitudents in (^Mes^PDP^Ph^)UO_2_(THF). As a consequence, the *ortho* chlorides in the canted Cl_2_Ph group have distances to the uranium center of 3.503 Å (U1–Cl1) and 5.503 Å (U1–Cl2), whereas the other Cl_2_Ph group has U–Cl distances of 4.688 Å (U1–Cl3) and 4.488 Å (U1–Cl4), respectively. The U1–Cl1 distance in (^Cl2Ph^PDP^Ph^)UO_2_(THF) is significantly longer than Cl → U dative interactions (3.006(4)–3.227 (3) Å) involving the *ortho*-Cl atoms of C_6_Cl_5_ ligands in a pair of U(IV) complexes characterized by Hayton and co-workers.^99^ Moreover, the solid-state structure of (^Cl2Ph^PDP^Ph^)UO_2_(THF) is *C*_1_ symmetric. This result is inconsistent with the NMR data, which indicates that the complex possesses *C*_2*v*_ symmetry in the solution. We propose that in the solution, (^Cl2Ph^PDP^Ph^)UO_2_(THF) does not engage in any interactions with the *ortho*-chlorides, or that there is fast equilibrium of the Cl_2_Ph moieties on the NMR timescale. Despite the differences that the Cl_2_Ph_2_ substitution pattern imparts relative to the mesityl analogue, the remainder of the structure of (^Cl2Ph^PDP^Ph^)UO_2_(THF) is consistent with that of (^Mes^PDP^Ph^)UO_2_(THF). The uranyl bond lengths were determined to be 1.771(3) Å (U(1)–O(1)) and 1.771(2) Å (U(1)–O(2)) and the O(1)–U(1)–O(2) bond angle is 174.67(11)°. The primary coordination sphere is completed by bonds to the PDP pincer nitrogens (U(1)–N(*X*) (*X* = 1, 2, 3) lengths of 2.381(3), 2.504(3), and 2.367(3) Å), and to the THF oxygen, (U(1)–O(3)) with a bond distance of 2.408(2) Å. The O(3)–U(1)–N(2) and N(3)–U(1)–N(1) bond angles were determined to be 174.76(10) and 130.61(9)°, respectively. A summary of selected bond lengths and angles for both (^Cl2Ph^PDP^Ph^)UO_2_(THF) and (^Mes^PDP^Ph^)UO_2_(THF) is provided in Table 1.

**Figure 4:**
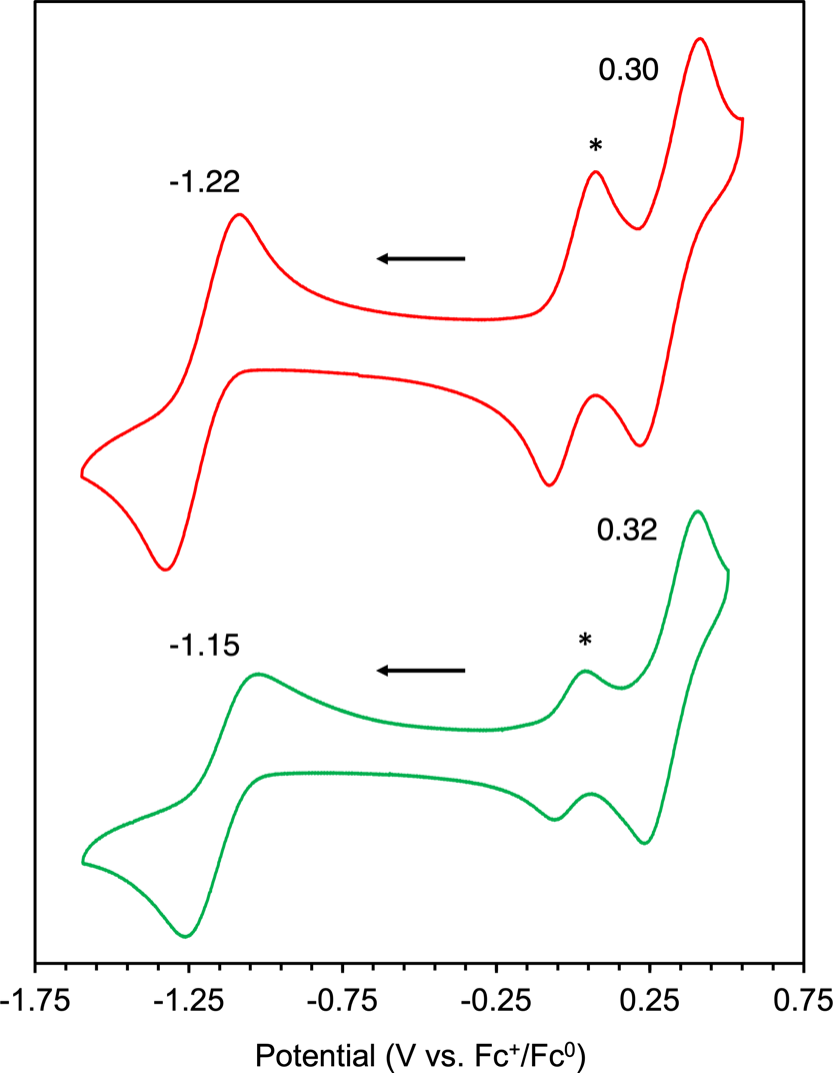
Cyclic voltammograms of 1 mM solutions of (^Mes^PDP^Ph^)UO_2_(THF) (red) and (^Cl2Ph^PDP^Ph^)UO_2_(THF) (green) with 100 mM [N^n^Bu_4_][PF_6_] supporting electrolyte in THF. Scan rate = 200 mV s^–1^. The middle redox couple in each voltammogram corresponds to the ferrocene reference, as indicated with an asterix (*). The black arrow indicates the direction of the scan.

Having established a firm understanding of the solution- and solid-state structures of both (^Ar^PDP^Ph^)UO_2_(L) complexes, we next pursued studies aimed at investigating the electrochemical properties of these compounds by means of cyclic voltammetry (CV;Figure 4). CV measurements of (^Mes^PDP^Ph^)UO_2_(THF) and (^Cl2Ph^PDP^Ph^)UO_2_(THF) were conducted on 1 mM solutions of the indicated compound in THF with 100 mM tetrabutylammonium hexafluorophosphate ([^n^Bu_4_N][PF_6_]) as the supporting electrolyte. For (^Mes^PDP^Ph^)UO_2_(THF), scanning anodically revealed a feature at 0.30 V (vs Fc^+/0^). This redox event is tentatively assigned as an oxidation of the PDP chelate ligand, as the uranyl center is in its fully oxidized (U^VI^) state. Noteworthy is that this event is cathodically shifted by over 200 mV relative to an analogous redox process for the Zr bis-PDP complex Zr(^Mes^PDP^Ph^)_2_ (*E*_1/2_ = 0.53 V vs Fc^+/0^).^67^ The apparent reversibility of this couple is surprising, as to date, Zr(^Mes^PDP^Ph^)_2_ is the only reported PDP complex to have reversible oxidative ligand chemistry. This is a consequence of the sterically protected pyrrolide functionality, which is normally susceptible to degradation under oxidizing conditions.^100^

When scanning cathodically, a reversible reduction event centered at −1.22 V (vs Fc^+/0^) is observed (see Figure S15 for peak dependence on the scan rate). Given the documented electrochemical stability of PDP ligands at similar potentials, we ascribed this event to a U^VI^/U^V^ redox couple.^66,69^ The reduction of the uranium center resembles values reported previously for the U^VI^/U^V^ redox couple for a number of uranyl compounds with nitrogen-based ligands, albeit anodically shifted by ∼100 mV in all cases.^95^ For example, the U^VI^/U^V^ reduction potential UO_2_(Ar_2_nacnac) (hfac) (Ar_2_nacnac= (2,6-^*i*^Pr_2_C_6_H_3_)NC(Me)CHC(Me)N(2,6-^*i*^Pr_2_C_6_H_3_)], hfac = hexafluoroacetylacetonate) reported by Hayton is −1.39 V versus Fc^+/0^.^101^ Furthermore, similar U^VI^/U^V^ reduction potentials (−1.32 and −1.36 V vs Fc^+/0^) have also been documented by Blakemore and co-workers for recently characterized heterobimetallic uranyl complexes.^102^ An additional irreversible reduction event is observed (*E*_pc_ = −3.07 V vs Fc^+/0^) and assigned as a one-electron reduction of the PDP chelate (Figure S14). Similar events at highly reducing potentials are well documented for previously reported transition metal PDP complexes; studies of reduced PDP Cr complexes have confirmed the nature of these events to be ligand based.^69^

The electrochemical profile of (^Cl2Ph^PDP^Ph^)UO_2_(THF) possessed similar features to that observed in the voltammagram of (^Mes^PDP^Ph^)UO_2_(THF). A reversible redox event is observed at 0.32 V versus Fc/Fc^+^ and is assigned as a ligand-based oxidation. The oxidation is shifted by 20 mV relative to (^Mes^PDP^Ph^)UO_2_(THF), consistent with the electron-withdrawing nature of the dichlorophenyl substituents of the pyrrolide moieties. Cathodic scans revealed a reversible reduction event centered at −1.15 V versus Fc^+/0^ (see Figure S16 for peak dependence on the scan rate). This feature is shifted anodically by 70 mV relative to the analogous process for (^Mes^PDP^Ph^)UO_2_(THF), suggesting the electron-withdrawing nature of the ligand further stabilizes the reduction of the uranyl ion. The electrochemical behavior of (^Cl2Ph^PDP^Ph^)UO_2_(THF) at extremely reducing potentials was similar to that of (^Mes^PDP^Ph^)UO_2_(THF), with a second reduction event assigned to the PDP ligand at −3.26 V. However, unlike (^Mes^PDP^Ph^)UO_2_(THF), in the case of (^Cl2Ph^PDP^Ph^)UO_2_(THF), this reduction event is entirely irreversible. Notably, the quasi-reversible U^VI/V^ redox process became strictly irreversible in these scans, supporting the rapid and complete decomposition of (^Cl2Ph^PDP^Ph^)UO_2_(THF) upon its two-electron reduction (Figure S14). Although the reason for this disparate behavior is not clear at this time, we propose that further one-electron reduction of putatitive [(^Cl2Ph^PDP^Ph^)UO_2_(THF)]^1–^ may result in reductive cleavage of an aryl chloride bond of one of the 2,6-dichlorophenyl substituents. Consistent with this hypothesis, the irreversible single-electron reduction of chlorobenzene has been reported at −3.23 V and results in the formation of an aryl radical and a chloride anion.^103^ Although outer-sphere fluoride abstraction from the [N^n^Bu_4_][PF_6_] supporting electrolyte could offer a similar halide-promoted degradation pathway, this possibility is disfavored in light of the quasi-reversible redox behavior observed for (^Mes^PDP^Ph^)UO_2_(THF) under the same experimental conditions.

In order to interrogate the optical properties of (^Mes^PDP^Ph^)UO_2_(THF) and (^Cl2Ph^PDP^Ph^)UO_2_(THF) in the solution phase we turned to electronic absorption spectroscopy. The spectra of both species in the THF solution are essentially identical (Figure S18) and are dominated by two charge-transfer bands at 326 and 424 nm (ε = 26,070 M^–1^ cm^–1^ and ε = 23,437 M^–1^ cm^–1^) for (^Mes^PDP^Ph^)UO_2_(THF) and 328 and 422 nm (ε = 24,052 M^–1^ cm^–1^ and ε = 18,062 M^–1^ cm^–1^) for (^Cl2Ph^PDP^Ph^)UO_2_(THF), respectively. It is noted that both complexes also weakly absorb past 500 nm. The electronic absorption spectra of both compounds are thus qualitatively similar to that of Sessler’s related pyrihexaphyrin (0.0.0.0.1.0)-uranyl complex, albeit only below 500 nm.^22,23^ Fortier’s family of DMAP-ligated bis-dipyrrinate compounds, which also have two pyrrolide and one pyridine donor, also possess similar charge-transfer bands, although the lower energy band, assigned as dipyrrin to uranium ligand-to-metal charge transfer (LMCT), is redshifted to 462–472 nm.^48^

**Figure 5:**
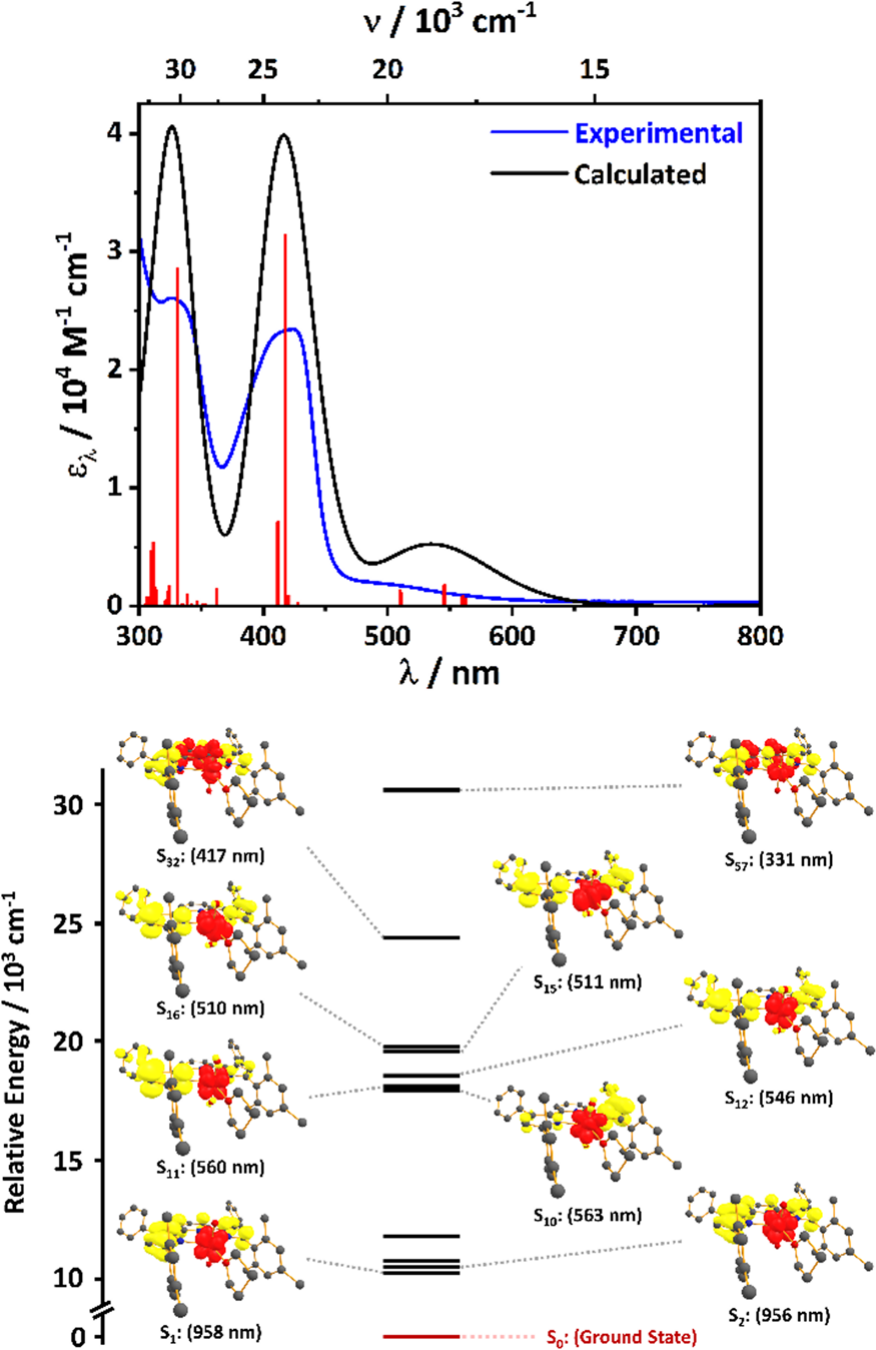
Top: lectronic absorption spectrum of (^Mes^PDP^Ph^)UO_2_(THF) in THF solution (blue) and the calculated spectrum (black). Vertical bars (red) indicate the position of the predicted transitions. Bottom: Relative energetic ordering of prominent electronic transitions from the TD-DFT calculations (bottom). The ground state (S_0_) is shown in maroon. Each state is represented by unrelaxed difference densities (red = gain of electron density and yellow = loss of electron density).

To gain further insight into the nature of the transitions in the electronic absorbance spectra of PDP complexes, time-dependent density functional theory (TD-DFT) calculations were undertaken on (^Mes^PDP^Ph^)UO_2_(THF) using ORCA quantum chemical program package v5.0.1. The C-PCM was utilized to account for solvation effects. Overall, the agreement between the calculated and experimental spectra is quite good (Computational SI Figure 1). Unfortunately, the lowest energy transitions are incredibly weak (Table 2), making them unlikely to be observable experimentally, especially because spin-orbit coupling (SOC) redshifts them to beyond 1200 nm in silico. It is clear, based on a visual analysis of the unrelaxed difference densities, that S_1_–S_4_ (the strongest low-energy transitions) are exclusively LMCT in character (Figure 5 and Computational Supporting Information Figure S5). This is further evidenced by a Mulliken population analysis on the ground and excited states which shows a charge reduction on uranium of nearly a full electron (Table 2). In addition, the experimental spectrum indicates an appreciable amount of intensity out to ca. 500–650 nm, which is consistent with the calculated spectrum, albeit with an overestimation of the intensity. The strongest transitions in this region are similarly assigned as LMCT based on visual inspection (Figure 5) and their population analyses (Table 2). The two strongest transitions, with calculated absorption maxima at 331 (S_57_) and 417 nm (S_32_), respectively, are primarily a π–π* transitions, although some LMCT character is mixed in Figure 5. This is further evidenced by a Mulliken population analysis which shows a minimal but nonzero charge reduction on uranium (Table 2).

**Table 2:** Summary of Relevant TD-DFT Calculated Excited States for (^Mes^PDP^Ph^)UO_2_(THF)

state	λ/nm	*f*_osc_	Δ*q*_Uranium_^a^/e	character^b^
1	958	0.0009	–0.959	LMCT
2	956	0.0014	–0.955	LMCT
3	939	0.0002	–0.969	LMCT
4	850	0.0004	–0.916	LMCT
10	563	0.0101	–0.918	LMCT
11	560	0.0125	–0.917	LMCT
12	546	0.0253	–0.922	LMCT
15	511	0.0158	–0.906	LMCT
16	510	0.0198	–0.909	LMCT
32	417	0.4359	–0.178	π–π*/LMCT
57	331	0.3961	–0.128	π–π*/LMCT

a Calculated by subtracting the Mulliken charges from the ground and excited state of interest (Δ*q* = *q*_ES_ – *q*_GS_).

b Assigned by visually inspecting the unrelaxed difference densities and analyzing the charge difference between the ground and excited state.

## Conclusions

We report the synthesis and characterization of two PDP uranyl complexes, (^Mes^PDP^Ph^)UO_2_(THF) and (^Cl2Ph^PDP^Ph^)UO_2_(THF), which have been thoroughly characterized by a variety of physical methods and a complementary TD-DFT study. Notably, the steric pressure imparted by the bulky aryl substituted PDP chelates results in six-coordinate distorted octahedral complexes in both the solid- and solution-state. The electronic absorption spectrum of (^Mes^PDP^Ph^)UO_2_(THF) is dominated by two charge-transfer bands at 326 and 424 nm with predominantly π–π* character, similar to previously reported uranyl complexes carrying pyrrole-based ligands, while the strongest transitions in the 500–600 nm region are assigned as LMCT. In THF solution, both (^Mes^PDP^Ph^)UO_2_(THF) and (^Cl2Ph^PDP^Ph^)UO_2_(THF) have been found to demonstrate rich electrochemistry, with reversible ligand-based oxidative chemistry and an apparent U^VI^/U^V^ reduction couple, which suggests that a reduced U^V^ analogue may be chemically isolable. In summary, we envision that this report will lay the foundation for future investigations of PDP actinide coordination and organometallic chemistry, including reactivity and photophysical studies.

## Supporting Information Available

Additional experimental procedures and spectroscopic and crystallographic data (PDF)
Computational details, input files, optimized coordinates, and vibrational frequencies, and references (PDF)

## Accession Codes

CCDC 2144690 and 2158758 contain the supplementary crystallographic data for this paper. These data can be obtained free of charge via www.ccdc.cam.ac.uk/data_request/cif, or by emailing data_request@ccdc.cam.ac.uk, or by contacting The Cambridge Crystallographic Data Centre, 12 Union Road, Cambridge CB2 1EZ, UK; fax: +44 1223 336033.
