# Supplementary material for: Synthesis and Characterization of Pyridine Dipyrrolide Uranyl Complexes
Source: Inorg Chem. 2022 Apr 14;61(16):6182–92. doi: 10.1021/acs.inorgchem.2c00348 (PMC9044449; doi:10.1021/acs.inorgchem.2c00348)
Supplement: Supplementary file 1 — ic2c00348_si_001.pdf [file ic2c00348_si_001.pdf]

# Electronic Supporting Information

# Synthesis and Characterization of Pyridine Dipyrrolide Uranyl Complexes

Brett M. Hakey[†], Dylan C. Leary[‡], Lauren M. Lopez[†], Leyla R. Valerio[†], William W. Brennessel[†], Carsten Milsmann[‡*], Ellen M. Matson[†*]

[†] Department of Chemistry, University of Rochester, Rochester, New York 14627, United States

[‡]C. Eugene Bennett Department of Chemistry, West Virginia University, Morgantown, West Virginia 26506, USA

*Corresponding author

**Supporting Information Table of Contents:**

**NMR Spectroscopy** ........................................ 1
**Figure S1.** $^{1}$H NMR spectrum of ($^{Mes}$PDP$^{Ph}$)$UO_2$(THF) in benzene-$d_6$ .......... 2
**Figure S2.** $^{1}$H NMR spectrum of ($^{Mes}$PDP$^{Ph}$)$UO_2$(THF) in dichloromethane-$d_2$ .......... 3
**Figure S3.** $^{13}$C NMR spectrum of ($^{Mes}$PDP$^{Ph}$)$UO_2$(THF) in benzene-$d_6$ .......... 4
**Figure S4.** $^{1}$H NMR spectrum of ($^{Cl2Ph}$PDP$^{Ph}$)$UO_2$(THF) in benzene-$d_6$ .......... 5
**Figure S5.** $^{1}$H NMR spectrum of ($^{Cl2Ph}$PDP$^{Ph}$)$UO_2$(THF) in dichloromethane-$d_2$ .......... 6
**Figure S6.** $^{13}$C NMR spectrum of ($^{Cl2Ph}$PDP$^{Ph}$)$UO_2$(THF) in benzene-$d_6$ .......... 7
**Figure S7.** Comparison of $^{1}$H NMR spectra of ($^{Ar}$PDP$^{Ph}$)$UO_2$(THF) .......... 8
**Figure S8.** $^{1}$H NMR spectrum of ($^{Mes}$PDP$^{Ph}$)$UO_2$(DMAP) in benzene-$d_6$ .......... 9
**Figure S9.** Infrared spectra of $H_2$$^{Mes}$PDP$^{Ph}$ and ($^{Mes}$PDP$^{Ph}$)$UO_2$(THF) .......... 10
**Figure S10.** Infrared spectra of $H_2$$^{Cl2Ph}$PDP$^{Ph}$ and ($^{Cl2Ph}$PDP$^{Ph}$)$UO_2$(THF) .......... 10
**Figure S11.** Infrared spectra of ($^{Mes}$PDP$^{Ph}$)$UO_2$(THF) and ($^{Cl2Ph}$PDP$^{Ph}$)$UO_2$(THF) .......... 11
**Figure S12.** Calculated (DFT) and experimental IR spectra of ($^{Mes}$PDP$^{Ph}$)$UO_2$(THF) .......... 12
**Figure S13.** CVs of ($^{Mes}$PDP$^{Ph}$)$UO_2$(THF) and ($^{Cl2Ph}$PDP$^{Ph}$)$UO_2$(THF) (-1.75 to -0.50 V) .......... 13
**Figure S14.** CVs of ($^{Ar}$PDP$^{Ph}$)$UO_2$(THF) (Ar = Mes, $Cl_2$Ph; -3.75 to +0.75 V) .......... 14
**Figure S15.** Scan rate dependence of CV of ($^{Mes}$PDP$^{Ph}$)$UO_2$(THF) (-1.75 to -0.50 V, THF) ...... 15
**Figure S16.** Scan rate dependence of CV of ($^{Cl2Ph}$PDP$^{Ph}$)$UO_2$(THF) (-1.75 to -0.50 V, THF) .... 15
**Figure S17.** Scan rate dependence of CV of ($^{Cl2Ph}$PDP$^{Ph}$)$UO_2$(THF) (-1.75 to -0.50 V, DCM)... 16
**Figure S18.** Electronic absorption spectra of ($^{Ar}$PDP$^{Ph}$)$UO_2$(THF) (Ar = Mes, $Cl_2$Ph; THF)...... 17
**Figure S19.** Electronic absorption spectra of $H_2$$^{Cl2Ph}$PDP$^{Ph}$ and ($^{Cl2Ph}$PDP$^{Ph}$)$UO_2$(THF) (THF) .. 17
**References** ........................................ 18
**Figure S20.** Space-filling diagram of structure of ($^{Mes}$PDP$^{Ph}$)$UO_2$(THF).......... 18
Complete X-ray Crystallographic Report for ($^{Mes}$PDP$^{Ph}$)$UO_2$(THF) .......... 20
Complete X-ray Crystallographic Report for ($^{Cl2Ph}$PDP$^{Ph}$)$UO_2$(THF) .......... 41

**NMR Spectroscopy**

In addition to the $^{1}H$ NMR characterization in $C_6D_6$ solution described in the article text, $^{1}H$ NMR spectra of both ($^{Mes}PDP^{Ph}$)$UO_2$(THF) and ($^{Cl2Ph}PDP^{Ph}$)$UO_2$(THF) were also obtained in dichloromethane-$d_2$.

($^{Mes}PDP^{Ph}$)$UO_2$(THF) (Figure S2): $^{1}H$ NMR (400 MHz, dichloromethane-$d_2$) δ 7.70 (d, *J* = 7.3 Hz, 4H, *o*-Ph*H*), 7.47 (t, *J* = 7.6 Hz, 4H, *m*-Ph*H*), 7.32-7.42 (m, 5H, p-Ph*H*, 3-pyridine*H*, 4-pyridine*H*), 6.16 (s, 2H, pyrrole*H*), 4.01 (s, 4H, THF-α-$CH_2$), 2.43 (s, 6H, *p*-MesC$H_3$), 2.20 (s, 12H, *o*-MesC$H_3$), 1.98 (m, 4H, THF-β-$CH_2$).

($^{Cl2Ph}PDP^{Ph}$)$UO_2$(THF) (Figure S2): $^{1}H$ NMR (400 MHz, dichloromethane-$d_2$) δ 7.69 (d, *J* = 7.1 Hz, 4H, Ar*H*), 7.57 (d, *J* = 8.1 Hz, 4H, Ar*H*), 7.48 (t, *J* = 7.6 Hz, 4H, *m*-Ph*H*), 7.42 – 7.32 (m, 7H, *p*-Ph*H*, *p*-$Cl_2$Ph*H*, 3-pyridine*H*, and 4-pyridine*H*), 6.53 (s, 2H, pyrrole*H*), 4.46 (m, 4H, THF-α-C$H_2$), 2.28 (m, 4H, THF-β-C$H_2$).

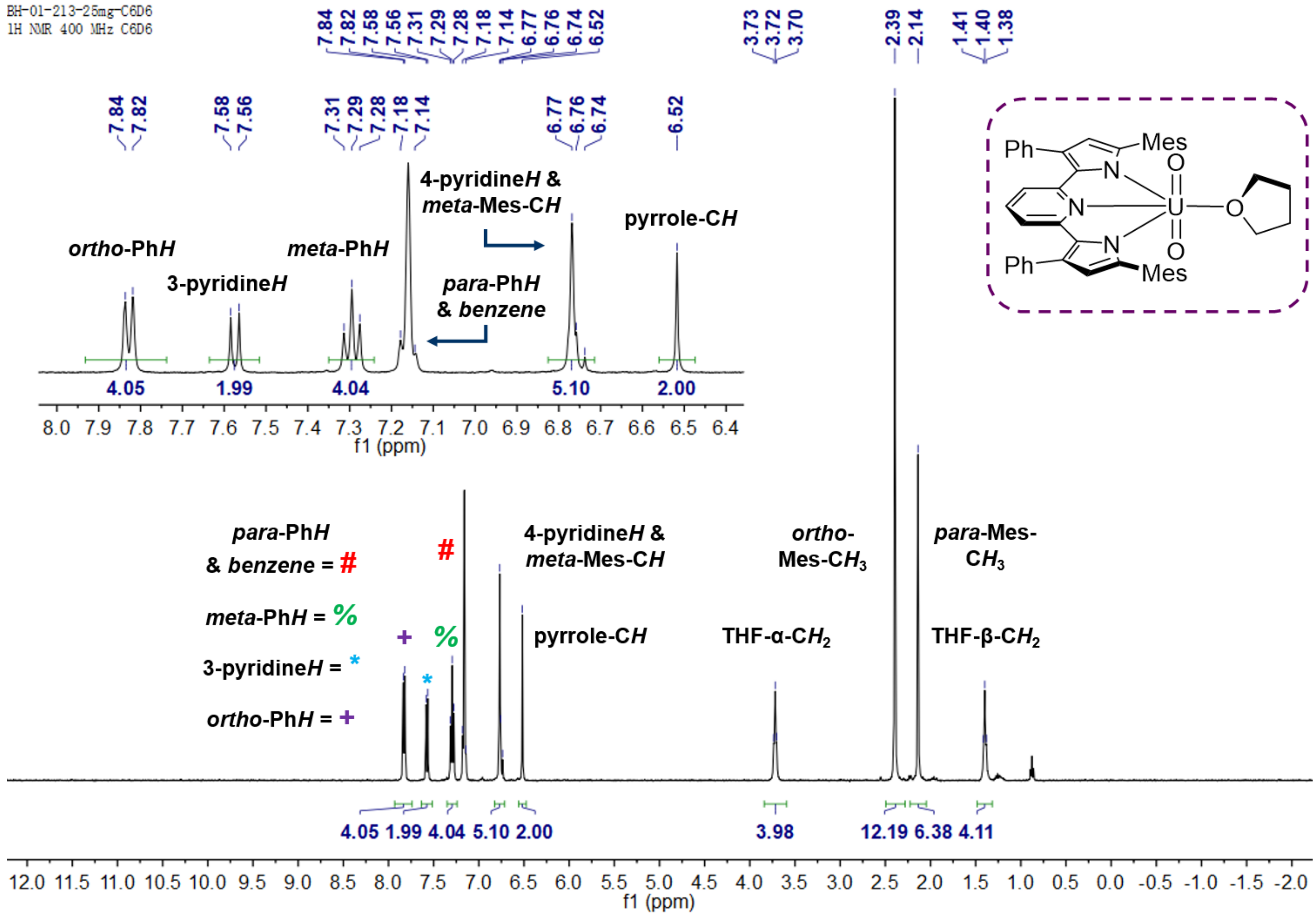


**Figure S1.** $^1$H NMR spectrum (400 MHz) of ($^{Mes}$PDP$^{Ph}$)$UO_2$(THF) in benzene-$d_6$. Note, the *para*-Ph*H* resonance overlaps with residual benzene. The unmarked resonances correspond to pentane present in the sample.

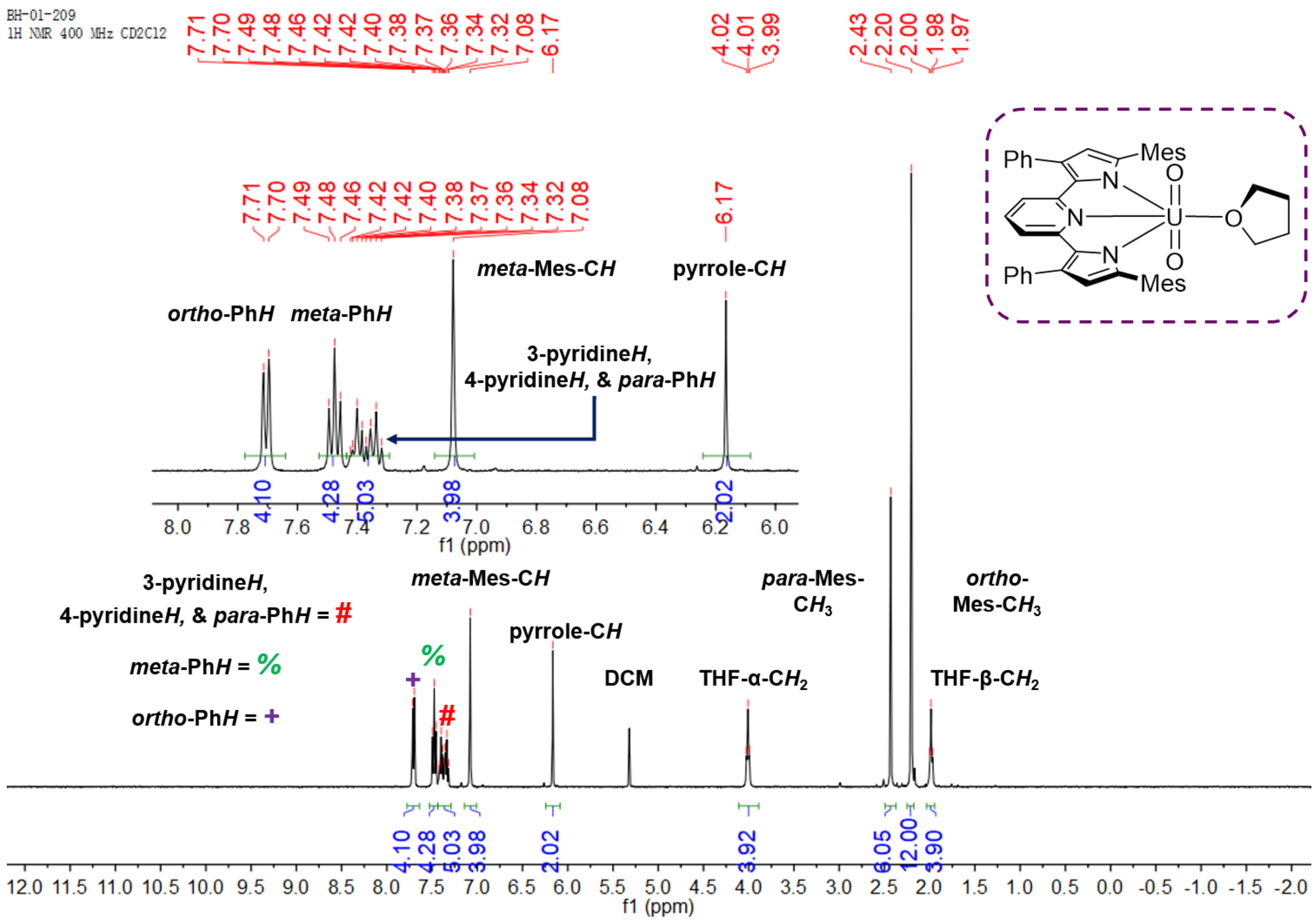


**Figure S2.** $^{1}$H NMR spectrum (400 MHz) of ($^{Mes}$PDP$^{Ph}$)$UO_2$(THF) in dichloromethane-$d_2$.

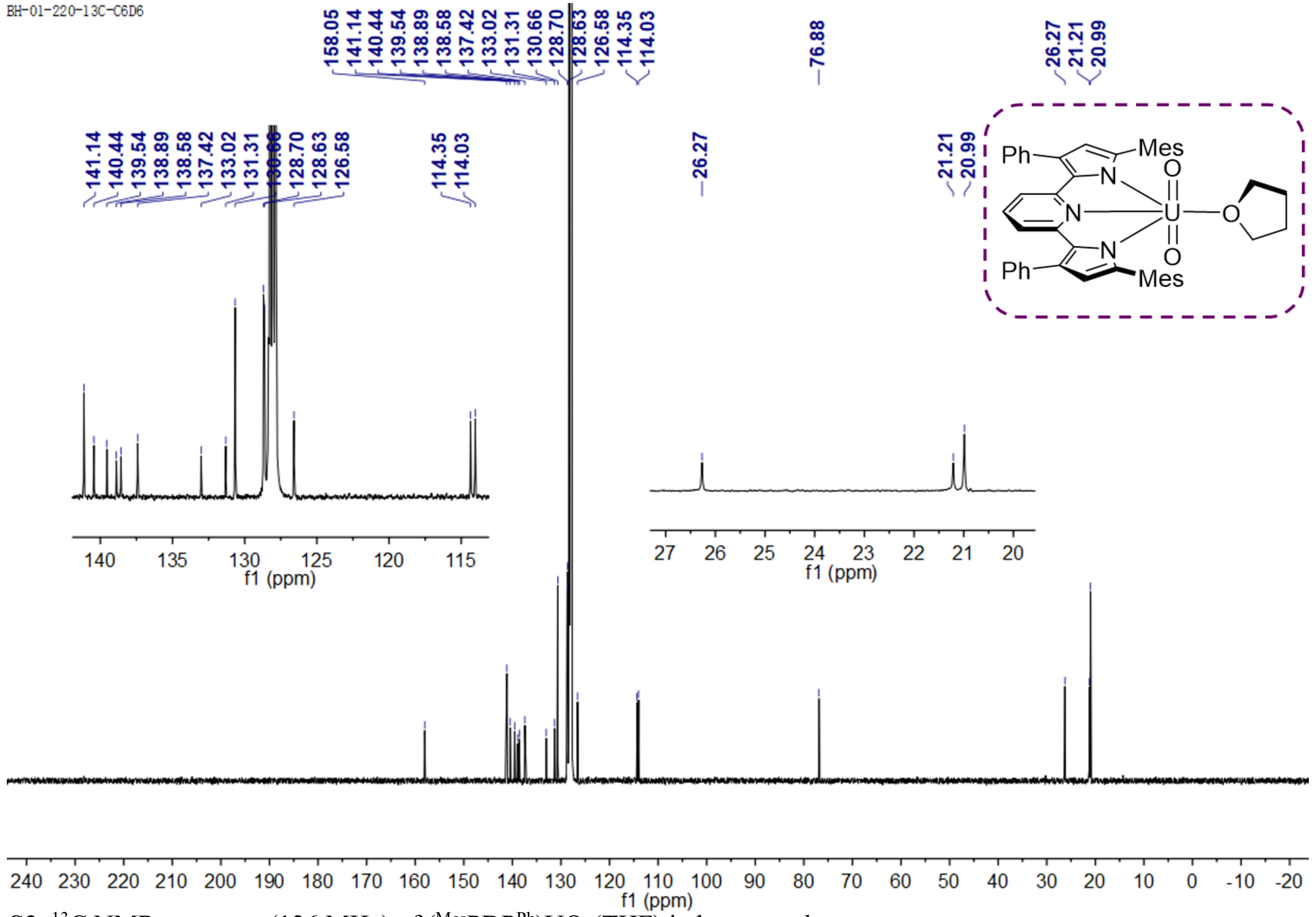


**Figure S3.** $^{13}C$ NMR spectrum (126 MHz) of ($^{Mes}PDP^{Ph}$)$UO_2$(THF) in benzene-$d_6$.

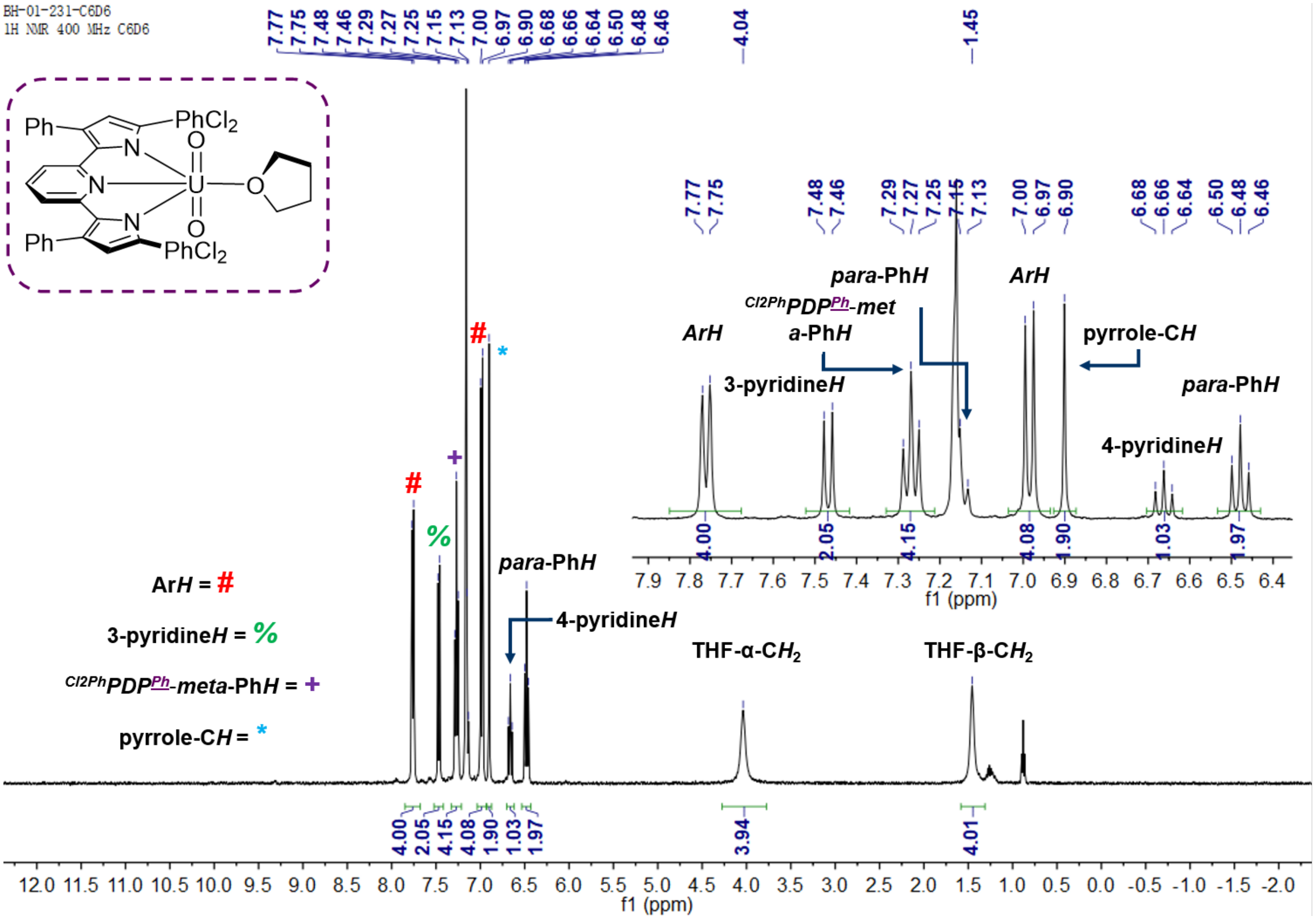


**Figure S4.** $^1$H NMR spectrum (400 MHz) of ($^{Cl2Ph}$PDP$^{Ph}$)$UO_2$(THF) in benzene-$d_6$. Note, a *para*-Ph*H* resonance overlaps with residual benzene. The unmarked resonances correspond to pentane in the sample.

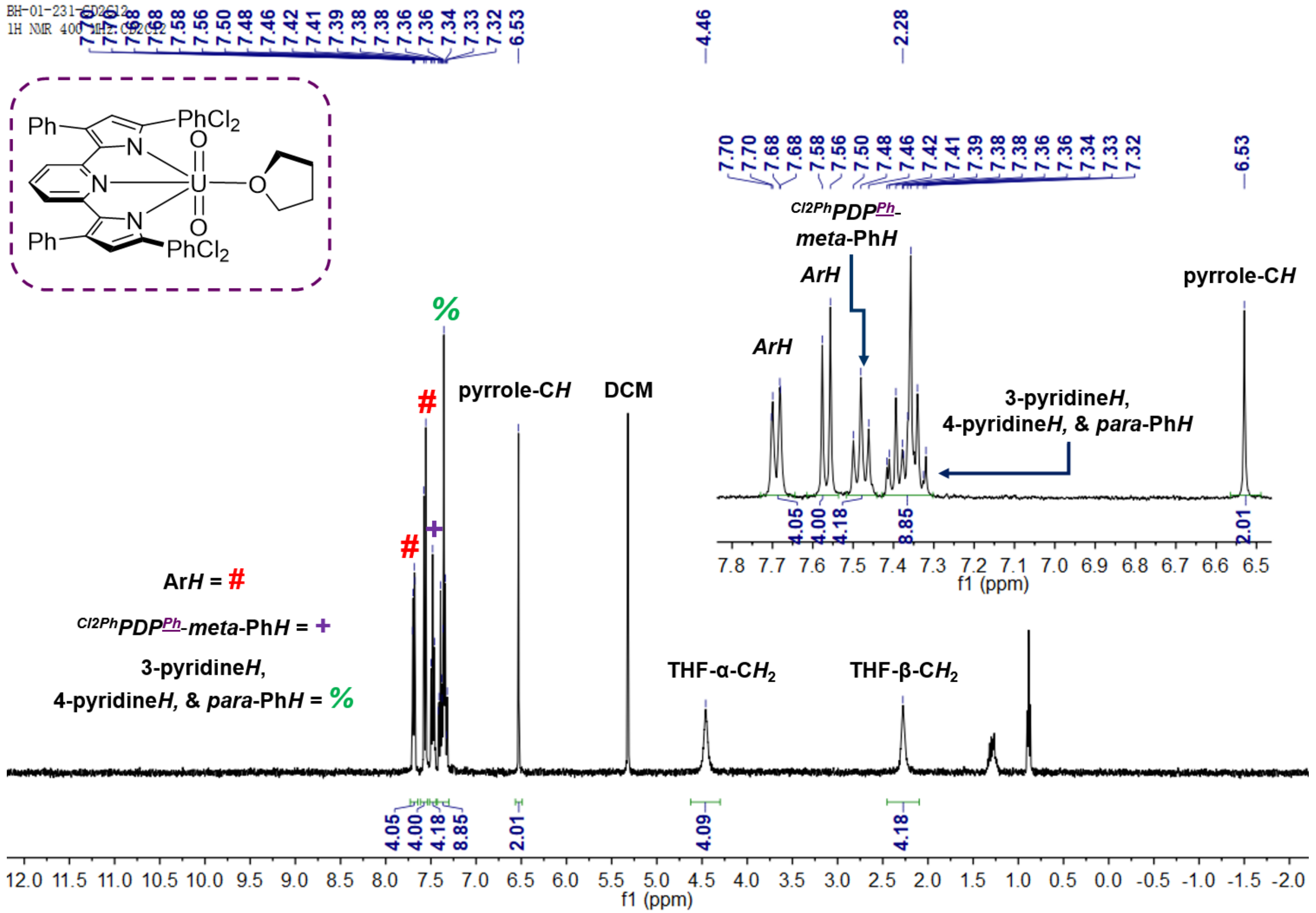


**Figure S5.** [1]H NMR spectrum (400 MHz) of ($^{Cl2Ph}$PDP$^{Ph}$)$UO_2$(THF) in dichloromethane-$d_2$. The unmarked resonances correspond to pentane in the sample.

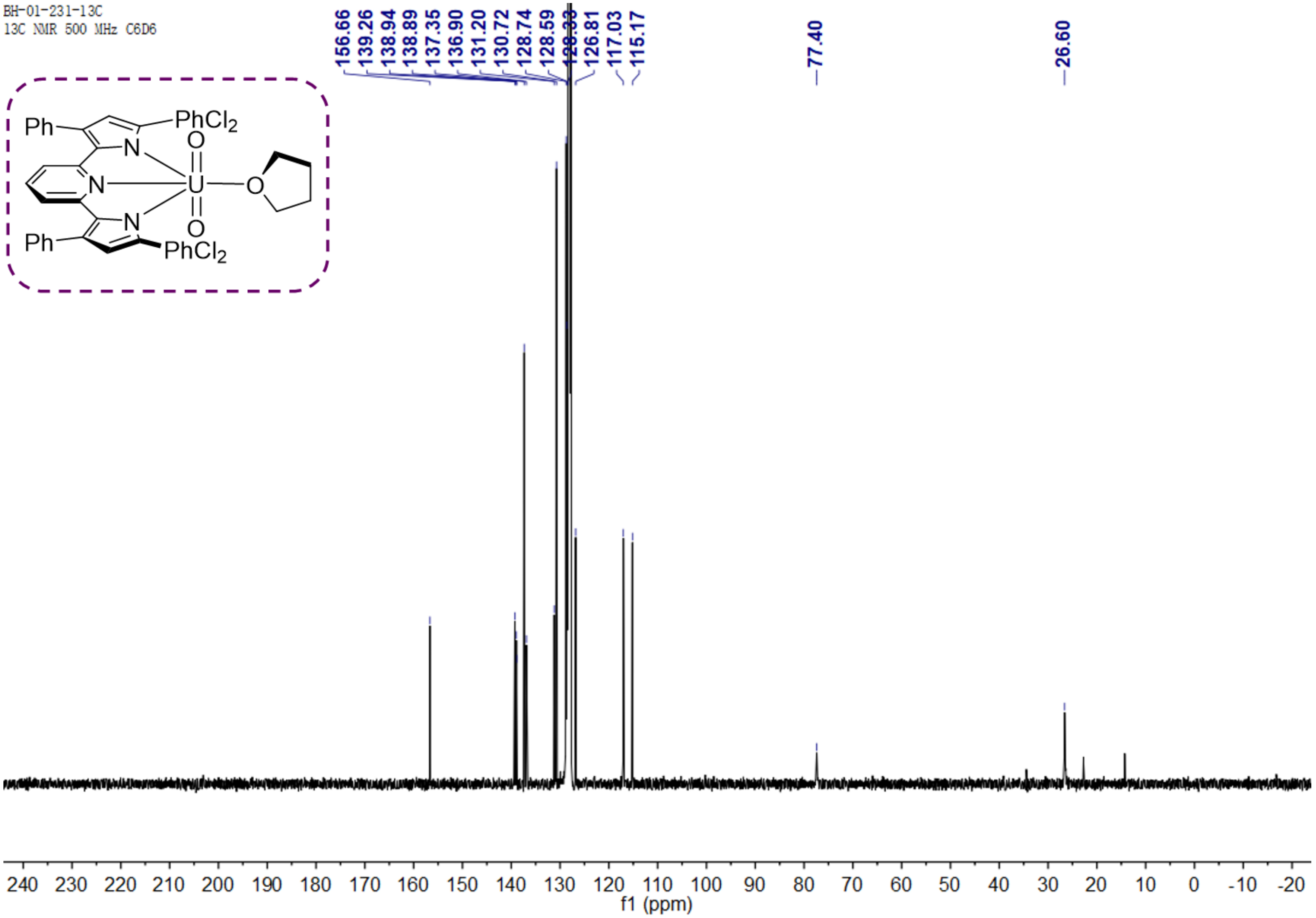


**Figure S6.** $^{13}$C NMR spectrum (126 MHz) of ($^{Cl2Ph}$PDP$^{Ph}$)$UO_2$(THF) in benzene-$d_6$. The unmarked resonances correspond to pentane.

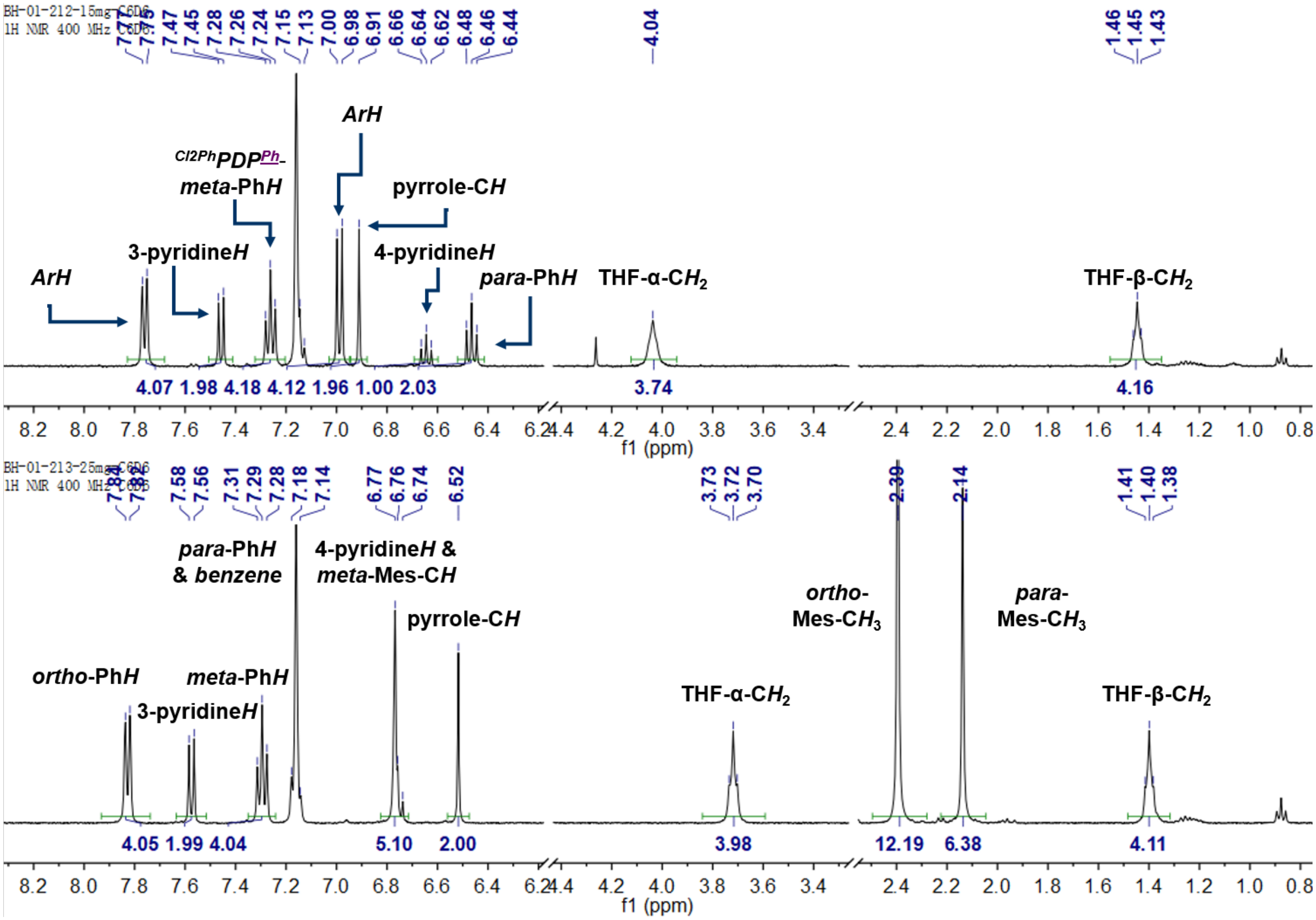


**Figure S7.** Comparison of the $^{1}$H NMR spectra (400 MHz) of $(^{Cl2Ph}PDP^{Ph})UO_2(THF)$ (top) and $(^{Mes}PDP^{Ph})UO_2(THF)$ (bottom) in benzene-$d_6$. The unmarked resonances correspond to dichloromethane and pentane solvent impurities in the samples.

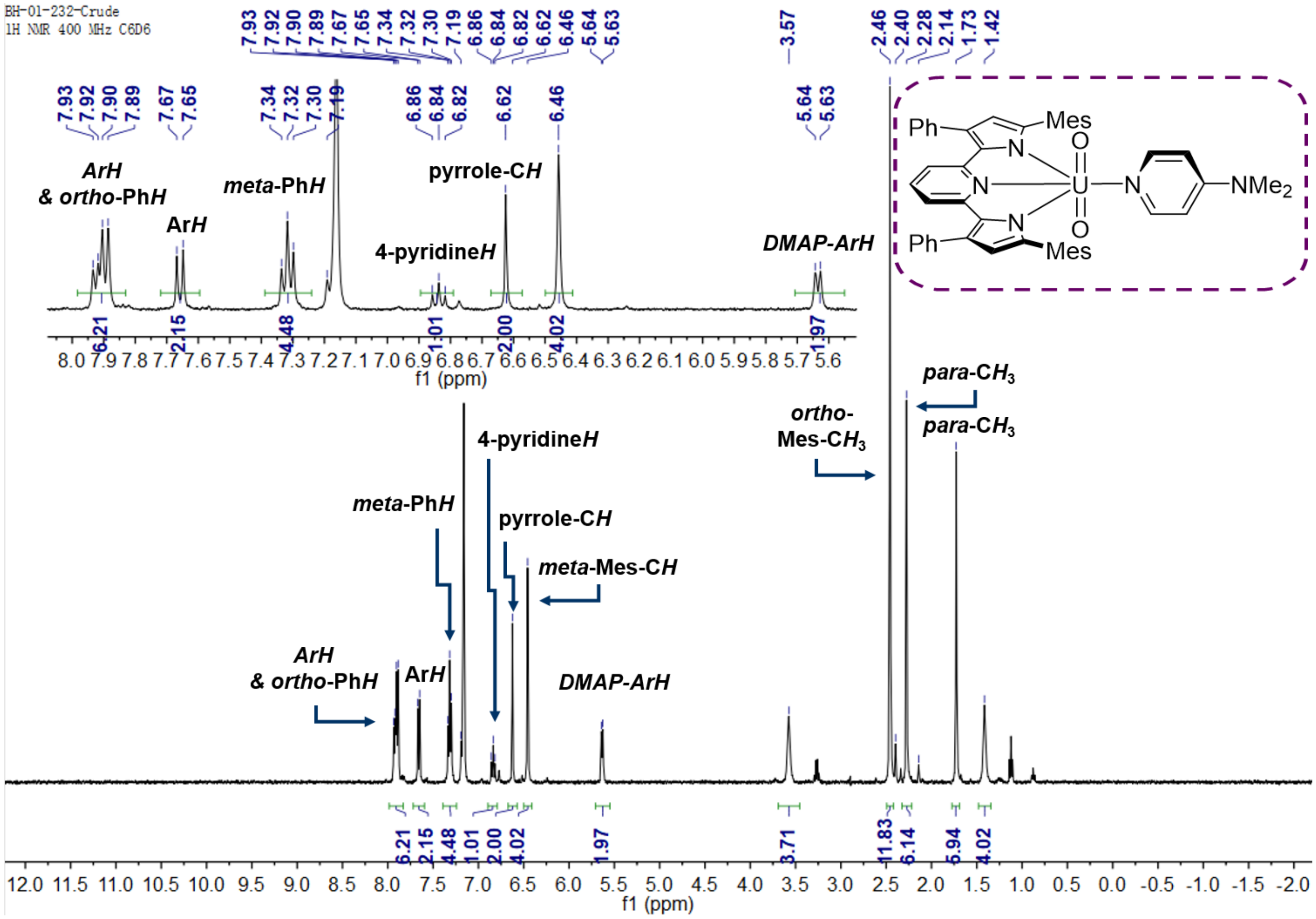


**Figure S8.** Crude $^{1}$H NMR spectra (400 MHz) ($^{Mes}$PDP$^{Ph}$)$UO_2$(DMAP) (DMAP = 4-dimethylaminopyridine) obtained from the reaction of ($^{Mes}$PDP$^{Ph}$)$UO_2$(THF) with DMAP in benzene-$d_6$. Note that one resonance (7.19 ppm) overlaps with the residual benzene signal. Singlet resonances corresponding to trace unreacted ($^{Mes}$PDP$^{Ph}$)$UO_2$(THF) are observed at 2.40 and 2.14 ppm. The resonances at 3.57 and 1.42 ppm correspond to free THF generated as a product of the reaction.[1] The unmarked resonances correspond to following diethyl ether and pentane solvent.

**IR Spectroscopy**

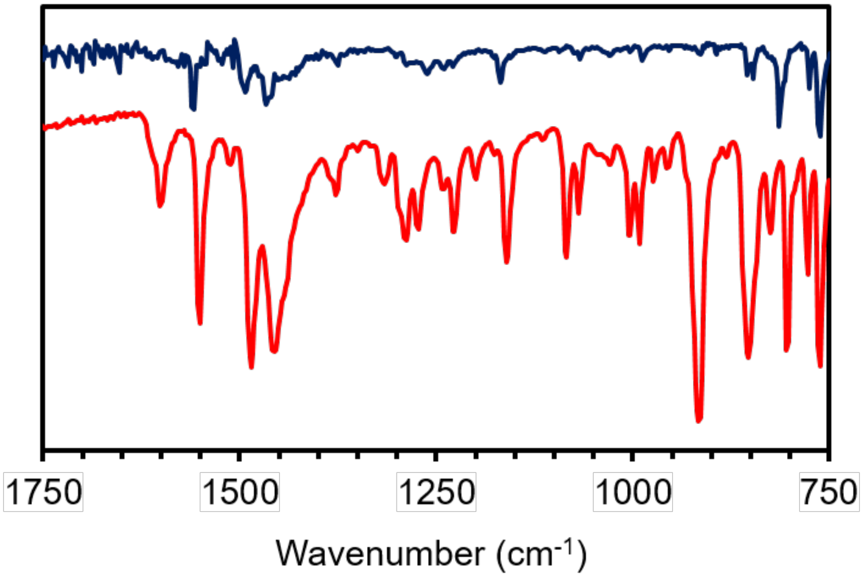


**Figure S9.** IR spectrum of $H_2$$^{Mes}$PDP$^{Ph}$ (dark blue) and ($^{Mes}$PDP$^{Ph}$)$UO_2$(THF) (red).

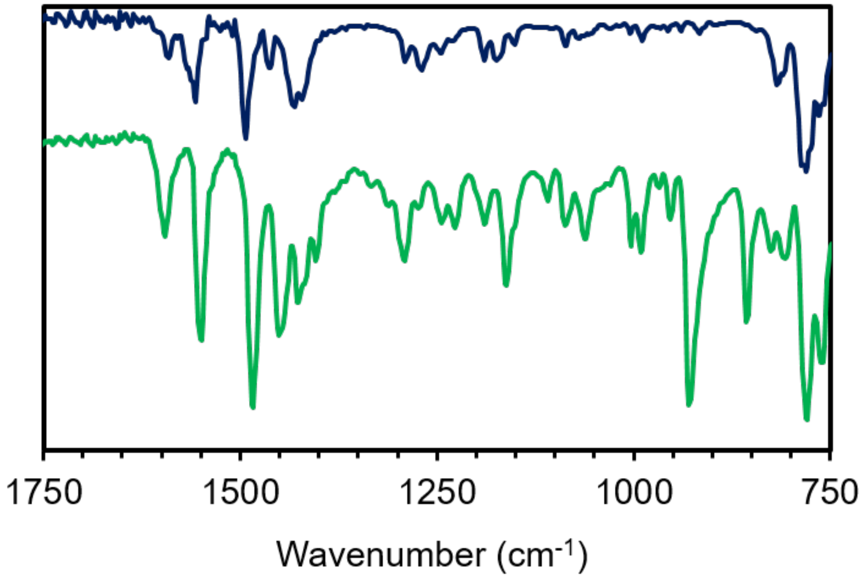


**Figure S10.** IR spectrum of $H_2$$^{Cl2Ph}$PDP$^{Ph}$ (dark blue) and ($^{Cl2Ph}$PDP$^{Ph}$)$UO_2$(THF) (green).

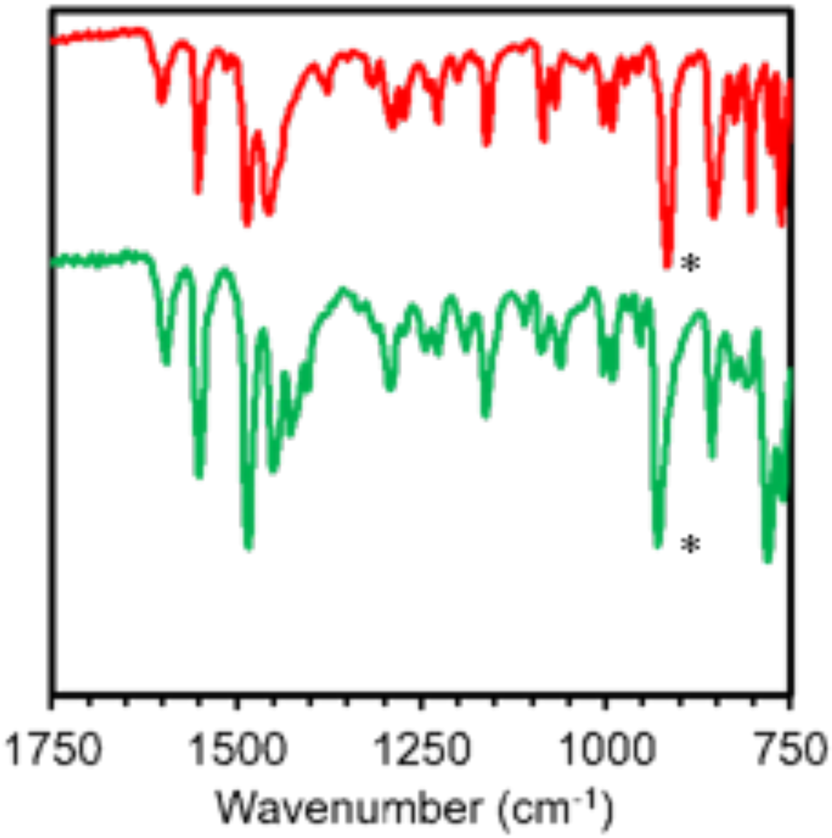


**Figure S11.** IR spectrum of ($^{Mes}$PDP$^{Ph}$)$UO_2$(THF) (red) and ($^{Cl2Ph}$PDP$^{Ph}$)$UO_2$(THF) (green).

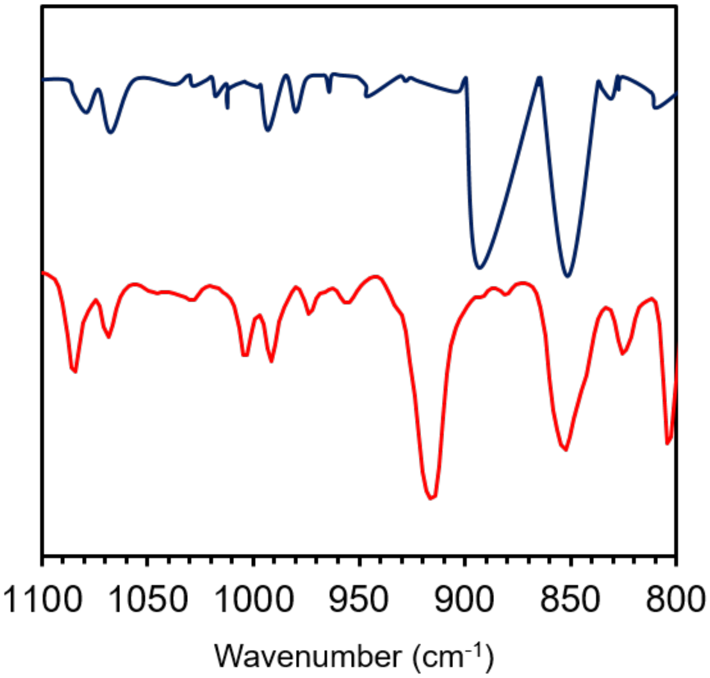


**Figure S12.** DFT calculated IR spectrum of ($^{Mes}$PDP$^{Ph}$)$UO_2$(THF) (dark blue) and experimental IR spectrum of ($^{Mes}$PDP$^{Ph}$)$UO_2$(THF) (red).

**Cyclic Voltammetry**

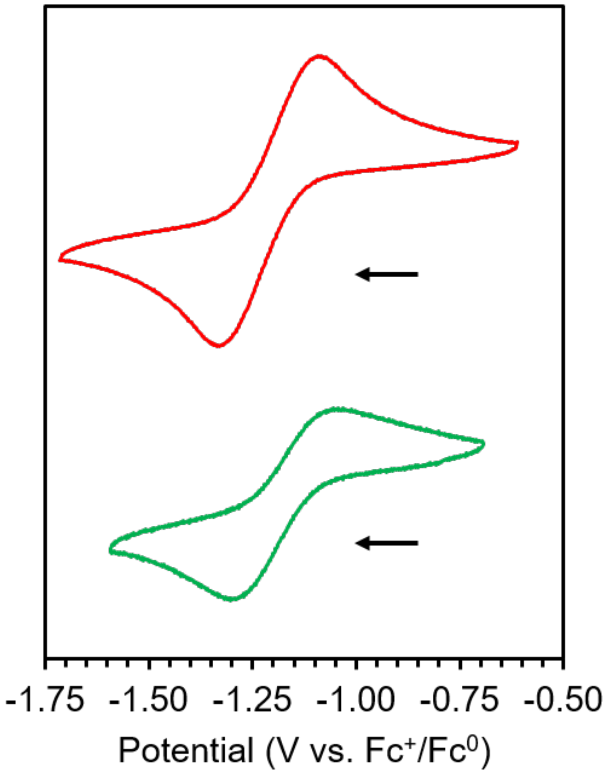


**Figure S13.** Cyclic voltammagrams of 1 mM solution of ($^{Mes}$PDP$^{Ph}$)$UO_2$(THF) (red) and ($^{Cl2Ph}$PDP$^{Ph}$)$UO_2$(THF) (green) with 100 mM [$N^nBu_4$][$PF_6$] supporting electrolyte in THF at scan rate of 200 $mVs^{-1}$. The black arrows indicate the direction of the scans.

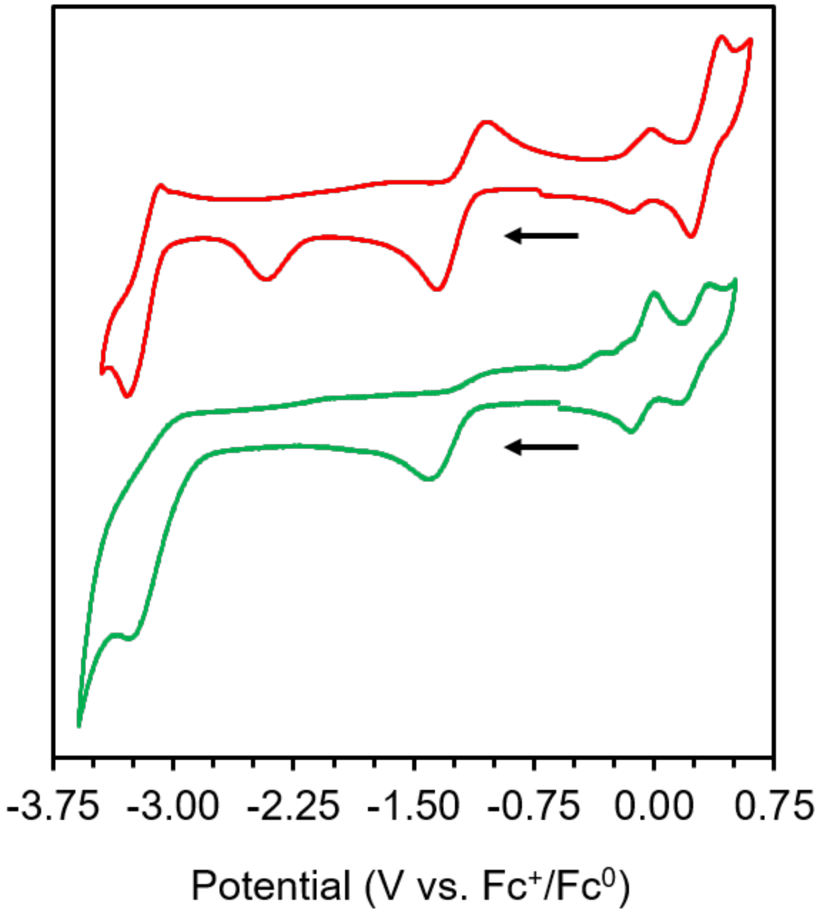


**Figure S14.** Cyclic voltammagrams of 1 mM solution of ($^{Mes}$PDP$^{Ph}$)$UO_2$(THF) (red) and ($^{Cl2Ph}$PDP$^{Ph}$)$UO_2$(THF) (green) with 100 mM [$N^nBu_4$][$PF_6$] supporting electrolyte in THF at 200 mVs$^{-1}$ scan rate. The black arrows indicates the direction of the scans.

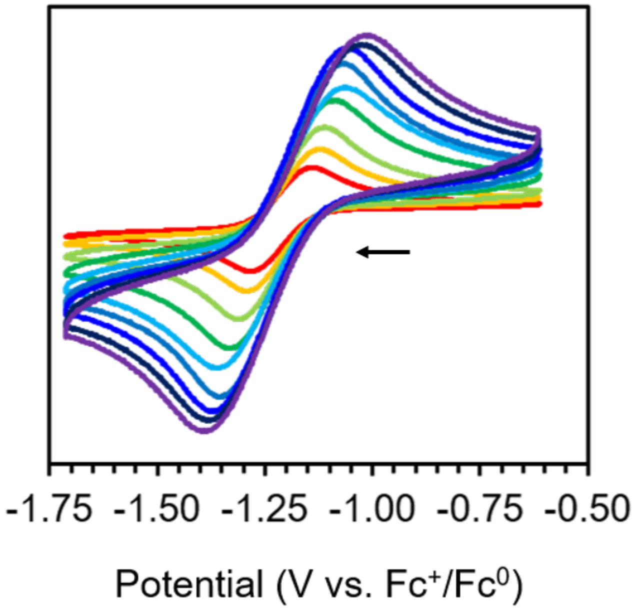


**Figure S15.** Cyclic voltammagrams of 1 mM solution of ($^{Mes}$PDP$^{Ph}$)$UO_2$(THF) with 100 mM [$N^nBu_4$][$PF_6$] supporting electrolyte in THF at varied scan rates (25, 50, 100, 200, 300, 400, 500, 600, and 700 $mVs^{-1}$). The black arrow indicates the direction of the scans.

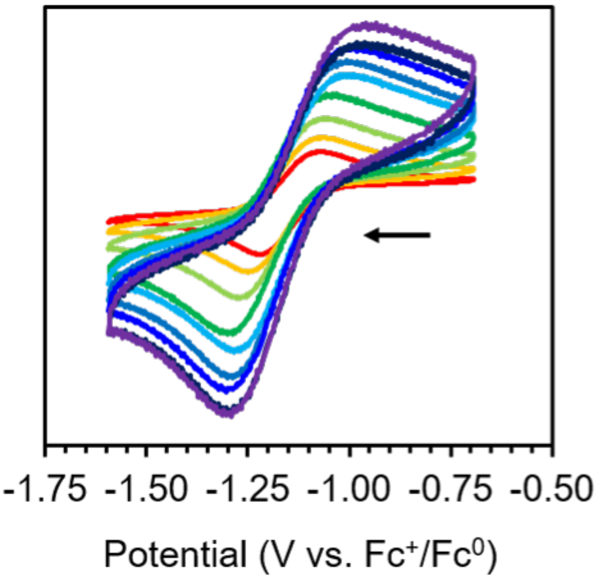


**Figure S16.** Cyclic voltammagrams of 1 mM solution of ($^{Cl2Ph}$PDP$^{Ph}$)$UO_2$(THF) with 100 mM [$N^nBu_4$][$PF_6$] in THF at varied scan rates (25, 50, 100, 200, 300, 400, 500, 600, and 700 $mVs^{-1}$). The black arrow indicates the direction of the scan.

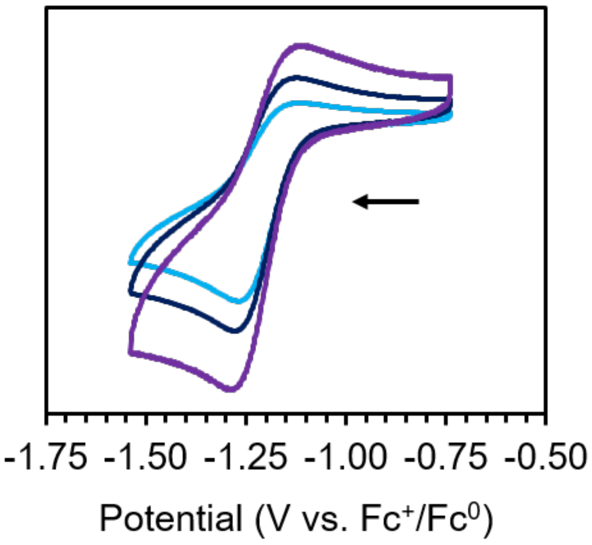


**Figure S17.** Cyclic voltammagrams of 1 mM solution of ($^{Cl2Ph}$PDP$^{Ph}$)$UO_2$(THF) with 100 mM [$N^nBu_4$][$PF_6$] in DCM at varied scan rates of 50 (light blue), 100 (dark blue) and 200 (purple) $mVs^{-1}$. The black arrow indicates the direction of the scans.

**UV-Vis Spectroscopy**

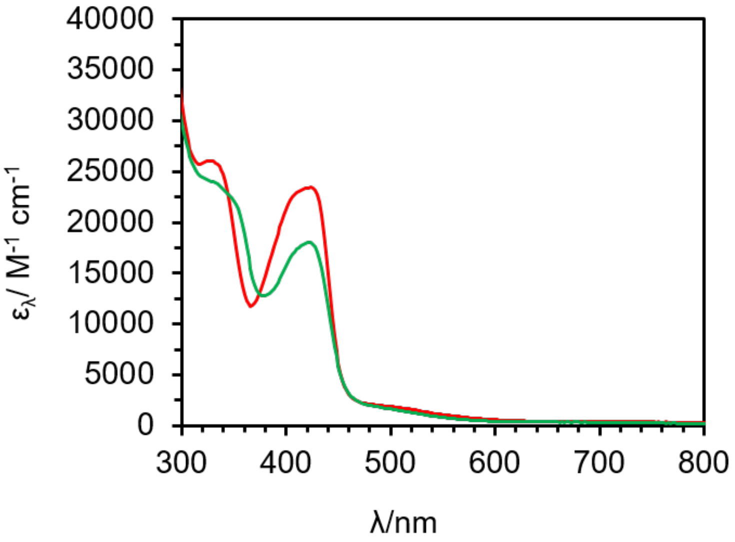


**Figure S18.** Electronic absorption spectrum of ($^{Mes}PDP^{Ph}$)$UO_2$(THF) (red) and ($^{Cl2Ph}PDP^{Ph}$)$UO_2$(THF) (green) in THF solution.

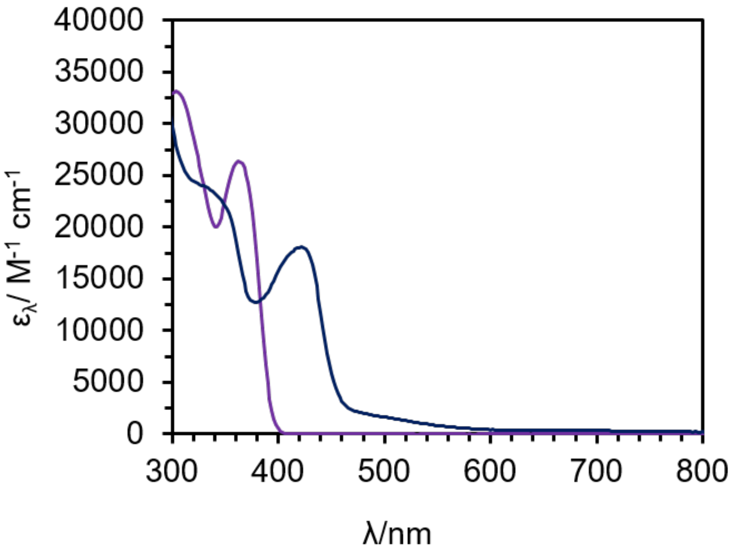


**Figure S19.** Electronic absorption spectrum of $H_2{}^{Cl2Ph}PDP^{Ph}$ (purple) and ($^{Cl2Ph}PDP^{Ph}$)$UO_2$(THF) (blue) in THF solution.

**References**

1.) Fulmer, G. R.; Miller, A. J. M.; Sherden, N. H.; Gottlieb, H. E.; Nudelman, A.; Stoltz, B. M.; Bercaw, J. E.; Goldberg, K. I. NMR Chemical Shifts of Trace Impurities: Common Laboratory Solvents, Organics, and Gases in Deuterated Solvents Relevant to the Organometallic Chemist. *Organometallics* **2010**, *29*, 2176–2179.

## X-ray Crystallography

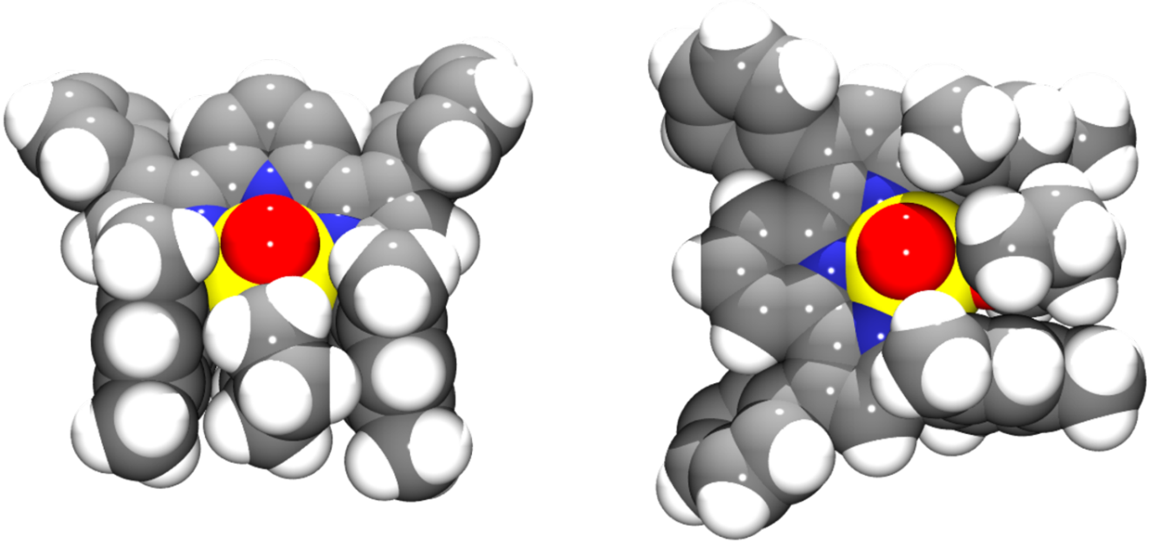

**Figure S20.** Space-filling diagrams of the X-ray crystal structure of ($^{Mes}$PDP$^{Ph}$)$UO_2$(THF).

REFERENCE NUMBER: matbh01

# CRYSTAL STRUCTURE REPORT

$C_{47} H_{45} N_3 O_3 U$

or

($^{Mes}PDP^{Ph}$)$UO_2$(thf)

Report prepared for:

Dr. B. Hakey, Prof. E. Matson

February 12, 2021

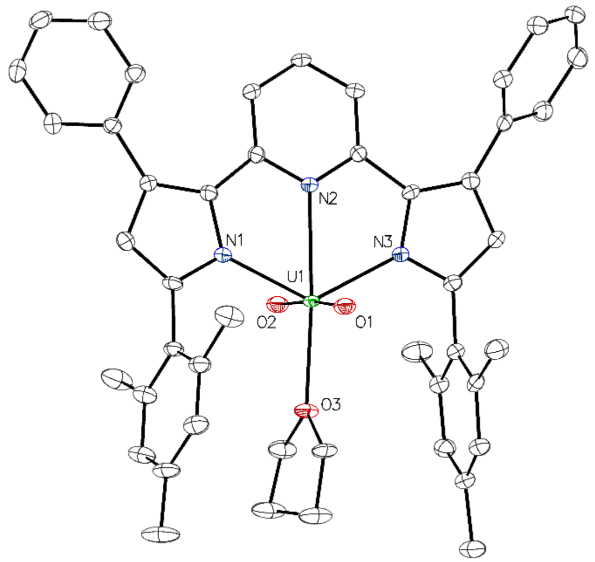



William W. Brennessel

X-ray Crystallographic Facility

Department of Chemistry, University of Rochester

120 Trustee Road

Rochester, NY 14627

## Data collection

A crystal ($0.12 \times 0.12 \times 0.12$ $mm^3$) was placed onto a thin glass optical fiber or a nylon loop and mounted on a Rigaku XtaLAB Synergy-S Dualflex diffractometer equipped with a HyPix-6000HE HPC area detector for data collection at 100.00(10) K. A preliminary set of cell constants and an orientation matrix were calculated from a small sampling of reflections.[1] A short pre-experiment was run, from which an optimal data collection strategy was determined. The full data collection was carried out using a PhotonJet (Cu) X-ray source with frame times of 0.24 and 0.96 seconds and a detector distance of 31.2 mm. Series of frames were collected in 0.50° steps in $\omega$ at different $2\theta$, $\kappa$, and $\phi$ settings. After the intensity data were corrected for absorption, the final cell constants were calculated from the xyz centroids of 43115 strong reflections from the actual data collection after integration.[1] See Table 1 for additional crystal and refinement information.

## Structure solution and refinement

The structure was solved using SHELXT[2] and refined using SHELXL.[3] The space group *I*2/*a* was determined based on systematic absences and intensity statistics. Most or all non-hydrogen atoms were assigned from the solution. Full-matrix least squares / difference Fourier cycles were performed which located any remaining non-hydrogen atoms. All non-hydrogen atoms were refined with anisotropic displacement parameters. All hydrogen atoms were placed in ideal positions and refined as riding atoms with relative isotropic displacement parameters. The final full matrix least squares refinement converged to $R1 = 0.0242$ ($F^2$, $I > 2\sigma(I)$) and $wR2 = 0.0656$ ($F^2$, all data).

## Structure description

The structure is the one suggested. The asymmetric unit contains one uranium complex in a general position.

Structure manipulation and figure generation were performed using Olex2.[4] Unless noted otherwise all structural diagrams containing anisotropic displacement ellipsoids are drawn at the 50 % probability level.

Data collection, structure solution, and structure refinement were conducted at the X-ray Crystallographic Facility, B04 Hutchison Hall, Department of Chemistry, University of Rochester. The instrument was purchased with funding from NSF MRI program grant CHE-1725028. All publications arising from this report MUST either 1) include William W. Brennessel as a coauthor or 2) acknowledge William W. Brennessel and the X-ray Crystallographic Facility of the Department of Chemistry at the University of Rochester.

[1] *CrysAlisPro*, version 171.41.97a; Rigaku Corporation: Oxford, UK, 2021.

[2] Sheldrick, G. M. *SHELXT*, version 2018/2; *Acta. Crystallogr.* **2015**, *A71*, 3-8.

[3] Sheldrick, G. M. *SHELXL*, version 2018/3; *Acta. Crystallogr.* **2015**, *C71*, 3-8.

[4] Dolomanov, O. V.; Bourhis, L. J.; Gildea, R. J.; Howard, J. A. K.; Puschmann, H. *Olex2*, version 1.3-ac4; *J. Appl. Cryst.* **2009**, *42*, 339-341.

Some equations of interest:

$$R_{int} = \Sigma |F_o^2 - < F_o^2 >| / \Sigma |F_o^2|$$

$$R1 = \Sigma ||F_o| - |F_c|| / \Sigma |F_o|$$

$$wR2 = [\Sigma[w(F_o^2 - F_c^2)^2] / \Sigma [w(F_o^2)^2]]^{1/2}$$

where $w = 1 / [\sigma^2 (F_o^2) + (aP)^2 + bP]$ and

$$P = 1/3 \max (0, F_o^2) + 2/3 F_c^2$$

$$GOF = S = [\Sigma[w(F_o^2 - F_c^2)^2] / (m-n)]^{1/2}$$

where $m$ = number of reflections and $n$ = number of parameters

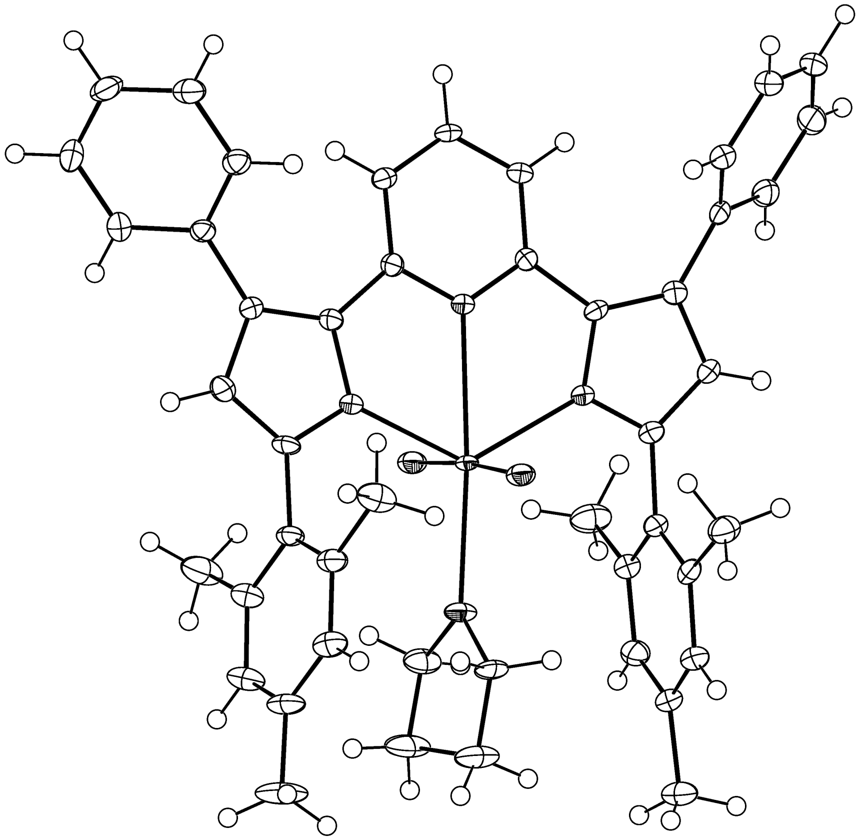

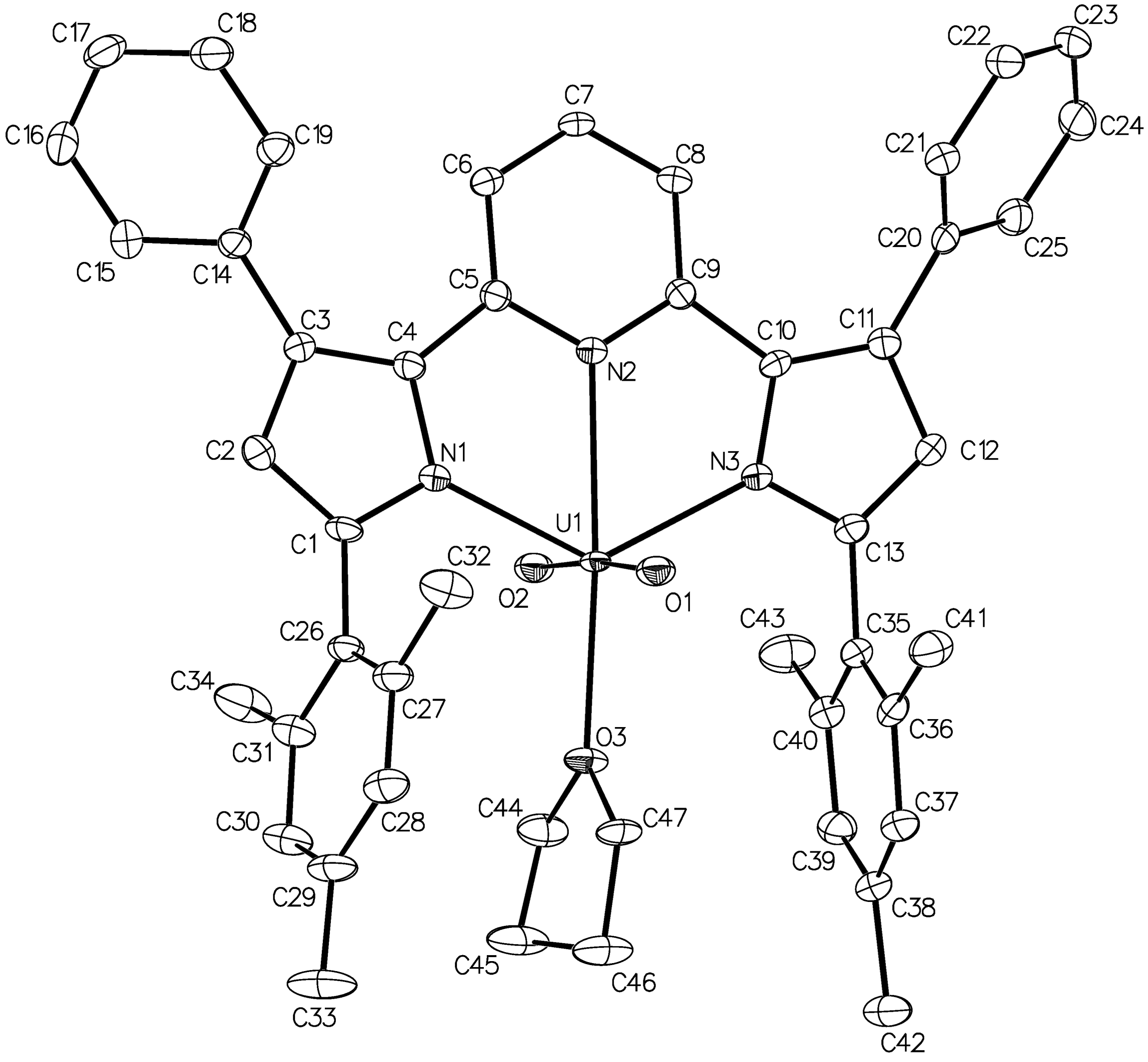
C17
C18
C23
C22
C7
C16
C19
C24
C21
C6
C8
C20
C25
C15
C14
C5
C9
C3
C4
C10
C11
N2
C2
N1
N3
C12
U1
C1
C13
C32
O2
O1
C43
C35
C41
C26
C34
C27
C40
C36
C31
O3
C37
C30
C28
C44
C47
C39
C29
C38
C45
C46
C33
C42

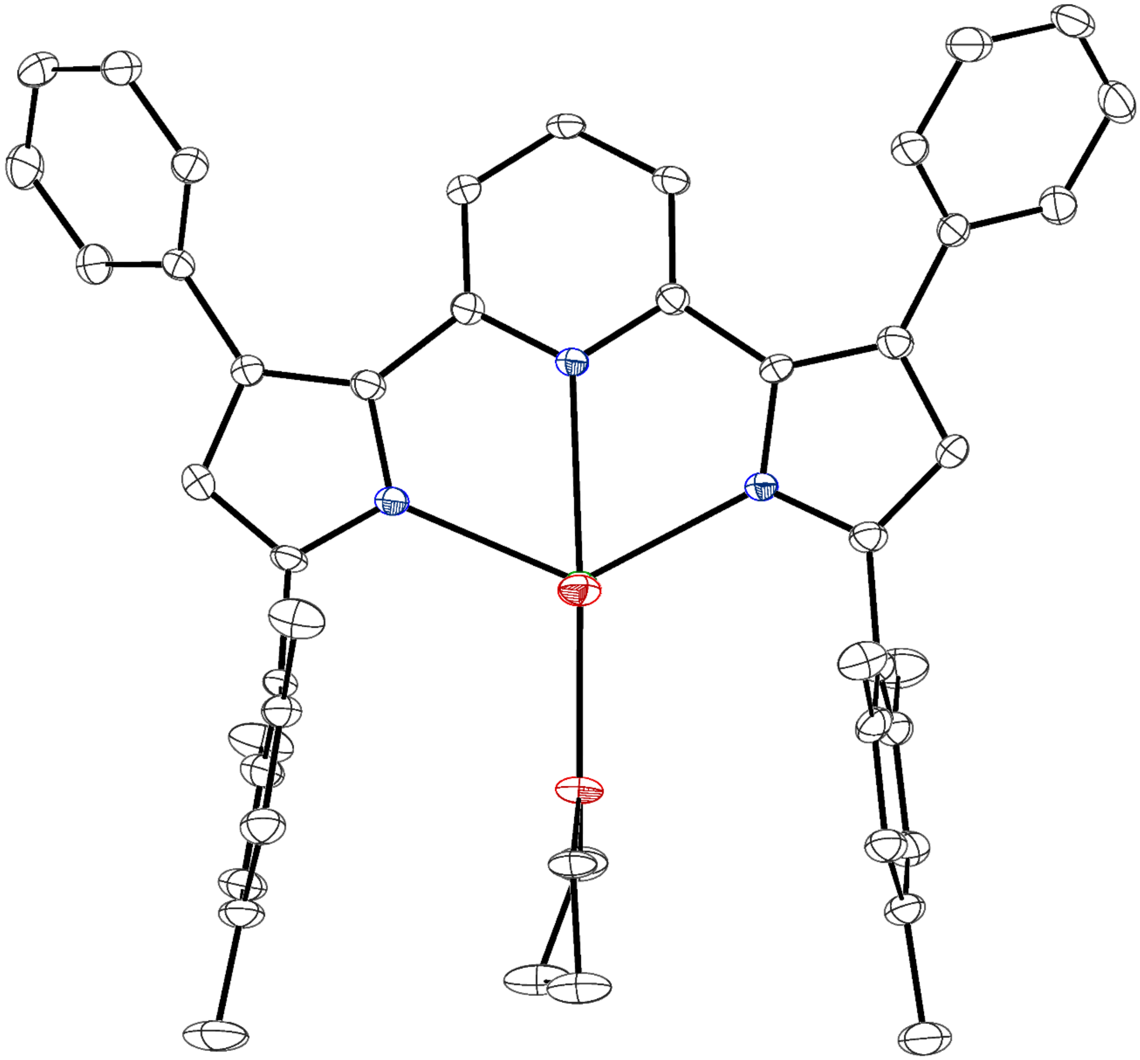

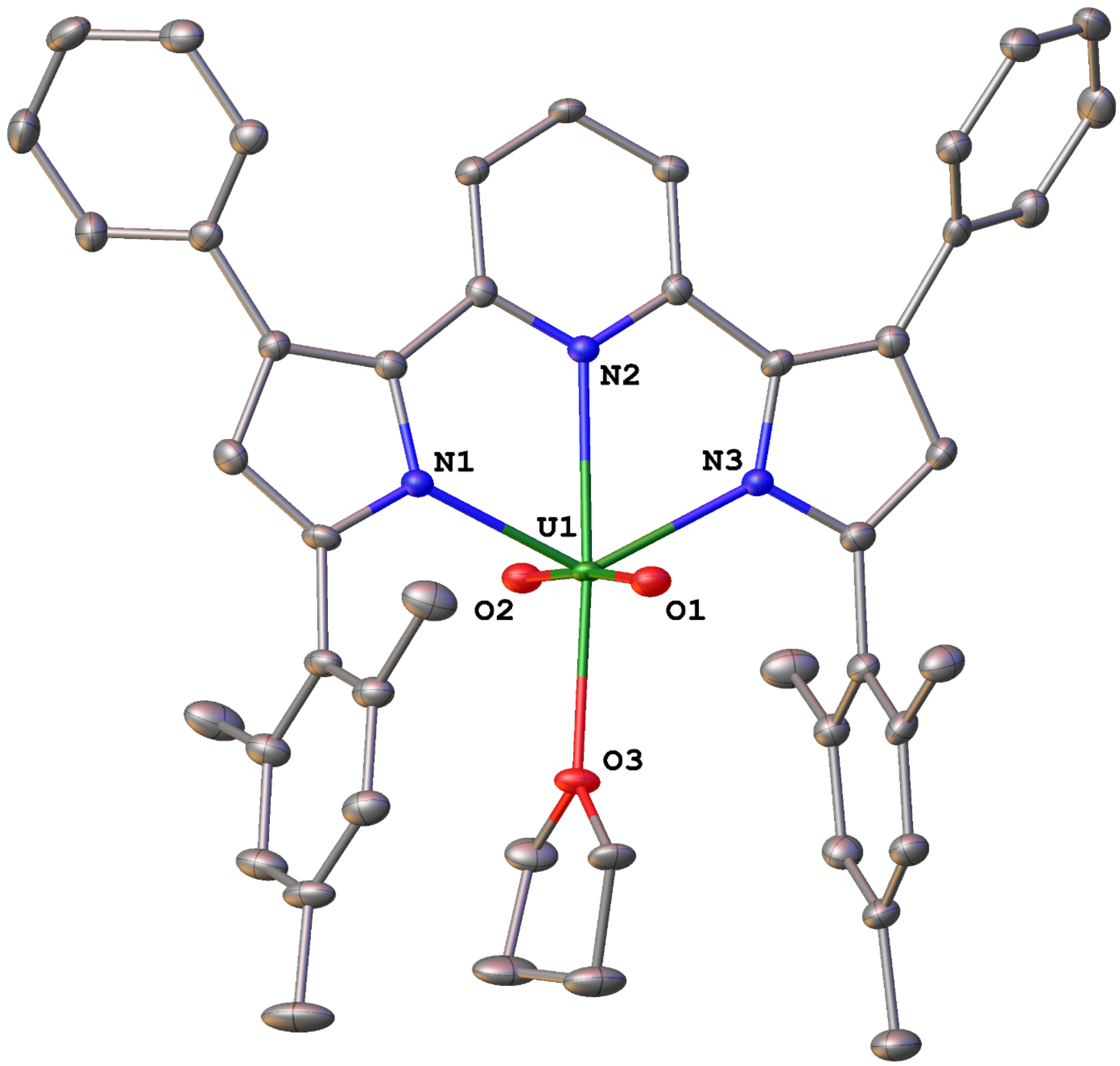
N2
N1
N3
U1
O2
O1
O3

Table 1. Crystal data and structure refinement for matbh01.

| | | |
|---|---|---|
| Identification code | matbh01 | |
| Empirical formula | C47 H45 N3 O3 U | |
| Formula weight | 937.89 | |
| Temperature | 100.00(10) K | |
| Wavelength | 1.54184 Å | |
| Crystal system | monoclinic | |
| Space group | *I*2/*a* | |
| Unit cell dimensions | *a* = 27.4429(3) Å | α = 90° |
| | *b* = 14.65870(10) Å | β = 116.991(2)° |
| | *c* = 22.6538(3) Å | γ = 90° |
| Volume | 8120.5(2) $Å^3$ | |
| *Z* | 8 | |
| Density (calculated) | 1.534 $Mg/m^3$ | |
| Absorption coefficient | 11.594 $mm^{-1}$ | |
| *F*(000) | 3712 | |
| Crystal color, morphology | orange-brown, needle | |
| Crystal size | 0.12 x 0.12 x 0.12 $mm^3$ | |
| Theta range for data collection | 3.515 to 77.817° | |
| Index ranges | $-34 \le h \le 34$, $-18 \le k \le 12$, $-28 \le l \le 28$ | |
| Reflections collected | 52491 | |
| Independent reflections | 8572 [*R*(int) = 0.0423] | |
| Observed reflections | 8429 | |
| Completeness to theta = 74.504° | 99.8% | |
| Absorption correction | Multi-scan | |
| Max. and min. transmission | 1.00000 and 0.57095 | |
| Refinement method | Full-matrix least-squares on $F^2$ | |
| Data / restraints / parameters | 8572 / 0 / 493 | |
| Goodness-of-fit on $F^2$ | 1.078 | |
| Final *R* indices [*I*>2sigma(*I*)] | *R*1 = 0.0242, *wR*2 = 0.0653 | |
| *R* indices (all data) | *R*1 = 0.0246, *wR*2 = 0.0656 | |
| Largest diff. peak and hole | 0.713 and -1.747 e.$Å^{-3}$ | |

Table 2. Atomic coordinates (x $10^4$) and equivalent isotropic displacement parameters ($Å^2$x $10^3$ ) for matbh01. $U_{eq}$ is defined as one third of the trace of the orthogonalized $U_{ij}$ tensor.

| | x | y | z | $U_{eq}$ |
|---|---|---|---|---|
| U1 | 5201(1) | 7578(1) | 6566(1) | 12(1) |
| O1 | 5500(1) | 7966(1) | 6064(1) | 20(1) |
| O2 | 4889(1) | 7312(1) | 7081(1) | 20(1) |
| O3 | 5065(1) | 9140(1) | 6758(1) | 20(1) |
| N1 | 4457(1) | 6952(2) | 5636(1) | 15(1) |
| N2 | 5278(1) | 5890(2) | 6379(1) | 14(1) |
| N3 | 6026(1) | 6877(2) | 7308(1) | 15(1) |
| C1 | 3993(1) | 7382(2) | 5213(2) | 16(1) |
| C2 | 3608(1) | 6743(2) | 4814(1) | 20(1) |
| C3 | 3856(1) | 5874(2) | 4986(1) | 16(1) |
| C4 | 4383(1) | 6026(2) | 5504(1) | 15(1) |
| C5 | 4834(1) | 5439(2) | 5916(1) | 14(1) |
| C6 | 4842(1) | 4490(2) | 5873(1) | 17(1) |
| C7 | 5304(1) | 4012(2) | 6295(1) | 18(1) |
| C8 | 5757(1) | 4473(2) | 6755(1) | 17(1) |
| C9 | 5736(1) | 5420(2) | 6790(1) | 15(1) |
| C10 | 6168(1) | 5990(2) | 7265(1) | 16(1) |
| C11 | 6706(1) | 5830(2) | 7746(1) | 18(1) |
| C12 | 6885(1) | 6661(2) | 8095(1) | 19(1) |
| C13 | 6464(1) | 7286(2) | 7809(2) | 18(1) |
| C14 | 3586(1) | 5021(2) | 4650(1) | 17(1) |
| C15 | 3056(1) | 4827(2) | 4553(2) | 25(1) |
| C16 | 2786(1) | 4047(2) | 4211(2) | 30(1) |
| C17 | 3038(1) | 3459(2) | 3961(2) | 29(1) |
| C18 | 3564(1) | 3637(2) | 4054(1) | 23(1) |
| C19 | 3832(1) | 4418(2) | 4388(1) | 19(1) |
| C20 | 7055(1) | 5022(2) | 7855(1) | 18(1) |
| C21 | 7080(1) | 4569(2) | 7325(2) | 21(1) |
| C22 | 7432(1) | 3829(2) | 7439(2) | 26(1) |
| C23 | 7766(1) | 3538(2) | 8085(2) | 29(1) |
| C24 | 7747(1) | 3988(2) | 8612(2) | 28(1) |

| | | | | |
|---|---|---|---|---|
| C25 | 7396(1) | 4722(2) | 8498(2) | 22(1) |
| C26 | 3949(1) | 8393(2) | 5168(1) | 17(1) |
| C27 | 4174(1) | 8856(2) | 4809(1) | 21(1) |
| C28 | 4094(1) | 9794(2) | 4710(2) | 25(1) |
| C29 | 3789(1) | 10281(2) | 4955(2) | 26(1) |
| C30 | 3578(1) | 9815(2) | 5321(2) | 27(1) |
| C31 | 3653(1) | 8878(2) | 5437(2) | 23(1) |
| C32 | 4479(1) | 8351(2) | 4495(2) | 30(1) |
| C33 | 3688(2) | 11286(2) | 4812(2) | 41(1) |
| C34 | 3409(2) | 8397(2) | 5827(2) | 35(1) |
| C35 | 6433(1) | 8269(2) | 7958(1) | 17(1) |
| C36 | 6591(1) | 8937(2) | 7637(2) | 19(1) |
| C37 | 6572(1) | 9851(2) | 7795(2) | 22(1) |
| C38 | 6410(1) | 10118(2) | 8272(2) | 22(1) |
| C39 | 6251(1) | 9441(2) | 8578(2) | 22(1) |
| C40 | 6253(1) | 8524(2) | 8427(1) | 19(1) |
| C41 | 6811(1) | 8690(2) | 7155(2) | 25(1) |
| C42 | 6422(1) | 11105(2) | 8463(2) | 31(1) |
| C43 | 6079(2) | 7823(2) | 8777(2) | 31(1) |
| C44 | 4816(1) | 9477(2) | 7170(2) | 26(1) |
| C45 | 4647(2) | 10436(2) | 6941(2) | 36(1) |
| C46 | 5064(2) | 10762(2) | 6733(2) | 35(1) |
| C47 | 5199(1) | 9921(2) | 6449(2) | 21(1) |

Table 3. Bond lengths [Å] and angles [°] for matbh01.

| | | | |
|---|---|---|---|
| U(1)-O(1) | 1.773(2) | C(13)-C(35) | 1.490(4) |
| U(1)-O(2) | 1.774(2) | C(14)-C(15) | 1.396(4) |
| U(1)-O(3) | 2.3917(19) | C(14)-C(19) | 1.397(4) |
| U(1)-N(1) | 2.354(2) | C(15)-H(15) | 0.9500 |
| U(1)-N(2) | 2.535(2) | C(15)-C(16) | 1.392(4) |
| U(1)-N(3) | 2.356(2) | C(16)-H(16) | 0.9500 |
| O(3)-C(44) | 1.472(3) | C(16)-C(17) | 1.377(5) |
| O(3)-C(47) | 1.472(3) | C(17)-H(17) | 0.9500 |
| N(1)-C(1) | 1.354(4) | C(17)-C(18) | 1.384(5) |
| N(1)-C(4) | 1.385(3) | C(18)-H(18) | 0.9500 |
| N(2)-C(5) | 1.365(3) | C(18)-C(19) | 1.386(4) |
| N(2)-C(9) | 1.364(3) | C(19)-H(19) | 0.9500 |
| N(3)-C(10) | 1.372(3) | C(20)-C(21) | 1.401(4) |
| N(3)-C(13) | 1.362(4) | C(20)-C(25) | 1.397(4) |
| C(1)-C(2) | 1.394(4) | C(21)-H(21) | 0.9500 |
| C(1)-C(26) | 1.487(4) | C(21)-C(22) | 1.394(4) |
| C(2)-H(2) | 0.9500 | C(22)-H(22) | 0.9500 |
| C(2)-C(3) | 1.413(4) | C(22)-C(23) | 1.394(5) |
| C(3)-C(4) | 1.405(4) | C(23)-H(23) | 0.9500 |
| C(3)-C(14) | 1.477(4) | C(23)-C(24) | 1.385(5) |
| C(4)-C(5) | 1.448(4) | C(24)-H(24) | 0.9500 |
| C(5)-C(6) | 1.395(4) | C(24)-C(25) | 1.387(4) |
| C(6)-H(6) | 0.9500 | C(25)-H(25) | 0.9500 |
| C(6)-C(7) | 1.383(4) | C(26)-C(27) | 1.399(4) |
| C(7)-H(7) | 0.9500 | C(26)-C(31) | 1.412(4) |
| C(7)-C(8) | 1.382(4) | C(27)-C(28) | 1.395(4) |
| C(8)-H(8) | 0.9500 | C(27)-C(32) | 1.514(4) |
| C(8)-C(9) | 1.393(4) | C(28)-H(28) | 0.9500 |
| C(9)-C(10) | 1.450(4) | C(28)-C(29) | 1.392(5) |
| C(10)-C(11) | 1.402(4) | C(29)-C(30) | 1.387(5) |
| C(11)-C(12) | 1.414(4) | C(29)-C(33) | 1.506(4) |
| C(11)-C(20) | 1.472(4) | C(30)-H(30) | 0.9500 |
| C(12)-H(12) | 0.9500 | C(30)-C(31) | 1.396(4) |
| C(12)-C(13) | 1.385(4) | C(31)-C(34) | 1.503(4) |

| | | | |
|---|---|---|---|
| C(32)-H(32A) | 0.9800 | C(46)-H(46B) | 0.9900 |
| C(32)-H(32B) | 0.9800 | C(46)-C(47) | 1.514(4) |
| C(32)-H(32C) | 0.9800 | C(47)-H(47A) | 0.9900 |
| C(33)-H(33A) | 0.9800 | C(47)-H(47B) | 0.9900 |
| C(33)-H(33B) | 0.9800 | O(1)-U(1)-O(2) | 173.94(9) |
| C(33)-H(33C) | 0.9800 | O(1)-U(1)-O(3) | 87.94(8) |
| C(34)-H(34A) | 0.9800 | O(1)-U(1)-N(1) | 90.98(8) |
| C(34)-H(34B) | 0.9800 | O(1)-U(1)-N(2) | 96.16(8) |
| C(34)-H(34C) | 0.9800 | O(1)-U(1)-N(3) | 91.28(9) |
| C(35)-C(36) | 1.401(4) | O(2)-U(1)-O(3) | 86.01(8) |
| C(35)-C(40) | 1.410(4) | O(2)-U(1)-N(1) | 91.24(9) |
| C(36)-C(37) | 1.394(4) | O(2)-U(1)-N(2) | 89.89(8) |
| C(36)-C(41) | 1.511(4) | O(2)-U(1)-N(3) | 91.70(9) |
| C(37)-H(37) | 0.9500 | O(3)-U(1)-N(2) | 175.38(7) |
| C(37)-C(38) | 1.399(4) | N(1)-U(1)-O(3) | 113.11(7) |
| C(38)-C(39) | 1.389(4) | N(1)-U(1)-N(2) | 64.79(7) |
| C(38)-C(42) | 1.506(4) | N(1)-U(1)-N(3) | 129.05(8) |
| C(39)-H(39) | 0.9500 | N(3)-U(1)-O(3) | 117.83(8) |
| C(39)-C(40) | 1.388(4) | N(3)-U(1)-N(2) | 64.36(7) |
| C(40)-C(43) | 1.502(4) | C(44)-O(3)-U(1) | 126.29(17) |
| C(41)-H(41A) | 0.9800 | C(44)-O(3)-C(47) | 109.2(2) |
| C(41)-H(41B) | 0.9800 | C(47)-O(3)-U(1) | 124.44(16) |
| C(41)-H(41C) | 0.9800 | C(1)-N(1)-U(1) | 127.39(19) |
| C(42)-H(42A) | 0.9800 | C(1)-N(1)-C(4) | 107.7(2) |
| C(42)-H(42B) | 0.9800 | C(4)-N(1)-U(1) | 123.97(17) |
| C(42)-H(42C) | 0.9800 | C(5)-N(2)-U(1) | 119.22(17) |
| C(43)-H(43A) | 0.9800 | C(9)-N(2)-U(1) | 120.02(17) |
| C(43)-H(43B) | 0.9800 | C(9)-N(2)-C(5) | 120.3(2) |
| C(43)-H(43C) | 0.9800 | C(10)-N(3)-U(1) | 125.06(17) |
| C(44)-H(44A) | 0.9900 | C(13)-N(3)-U(1) | 126.93(19) |
| C(44)-H(44B) | 0.9900 | C(13)-N(3)-C(10) | 107.7(2) |
| C(44)-C(45) | 1.496(5) | N(1)-C(1)-C(2) | 109.8(2) |
| C(45)-H(45A) | 0.9900 | N(1)-C(1)-C(26) | 122.2(3) |
| C(45)-H(45B) | 0.9900 | C(2)-C(1)-C(26) | 127.7(3) |
| C(45)-C(46) | 1.498(5) | C(1)-C(2)-H(2) | 126.3 |
| C(46)-H(46A) | 0.9900 | C(1)-C(2)-C(3) | 107.4(2) |

| | | | |
|---|---|---|---|
| C(3)-C(2)-H(2) | 126.3 | C(19)-C(14)-C(3) | 121.9(2) |
| C(2)-C(3)-C(14) | 123.6(2) | C(14)-C(15)-H(15) | 119.7 |
| C(4)-C(3)-C(2) | 105.8(2) | C(16)-C(15)-C(14) | 120.6(3) |
| C(4)-C(3)-C(14) | 130.6(2) | C(16)-C(15)-H(15) | 119.7 |
| N(1)-C(4)-C(3) | 109.3(2) | C(15)-C(16)-H(16) | 119.9 |
| N(1)-C(4)-C(5) | 116.3(2) | C(17)-C(16)-C(15) | 120.2(3) |
| C(3)-C(4)-C(5) | 134.4(3) | C(17)-C(16)-H(16) | 119.9 |
| N(2)-C(5)-C(4) | 114.3(2) | C(16)-C(17)-H(17) | 119.9 |
| N(2)-C(5)-C(6) | 120.0(2) | C(16)-C(17)-C(18) | 120.1(3) |
| C(6)-C(5)-C(4) | 125.7(2) | C(18)-C(17)-H(17) | 119.9 |
| C(5)-C(6)-H(6) | 120.1 | C(17)-C(18)-H(18) | 120.1 |
| C(7)-C(6)-C(5) | 119.7(2) | C(17)-C(18)-C(19) | 119.8(3) |
| C(7)-C(6)-H(6) | 120.1 | C(19)-C(18)-H(18) | 120.1 |
| C(6)-C(7)-H(7) | 120.0 | C(14)-C(19)-H(19) | 119.5 |
| C(8)-C(7)-C(6) | 120.1(3) | C(18)-C(19)-C(14) | 121.0(3) |
| C(8)-C(7)-H(7) | 120.0 | C(18)-C(19)-H(19) | 119.5 |
| C(7)-C(8)-H(8) | 120.5 | C(21)-C(20)-C(11) | 121.2(3) |
| C(7)-C(8)-C(9) | 119.0(3) | C(25)-C(20)-C(11) | 120.4(3) |
| C(9)-C(8)-H(8) | 120.5 | C(25)-C(20)-C(21) | 118.3(3) |
| N(2)-C(9)-C(8) | 120.9(2) | C(20)-C(21)-H(21) | 119.8 |
| N(2)-C(9)-C(10) | 113.8(2) | C(22)-C(21)-C(20) | 120.5(3) |
| C(8)-C(9)-C(10) | 125.2(2) | C(22)-C(21)-H(21) | 119.8 |
| N(3)-C(10)-C(9) | 115.9(2) | C(21)-C(22)-H(22) | 119.9 |
| N(3)-C(10)-C(11) | 109.7(2) | C(23)-C(22)-C(21) | 120.2(3) |
| C(11)-C(10)-C(9) | 134.2(3) | C(23)-C(22)-H(22) | 119.9 |
| C(10)-C(11)-C(12) | 105.4(2) | C(22)-C(23)-H(23) | 120.2 |
| C(10)-C(11)-C(20) | 130.0(3) | C(24)-C(23)-C(22) | 119.6(3) |
| C(12)-C(11)-C(20) | 124.4(2) | C(24)-C(23)-H(23) | 120.2 |
| C(11)-C(12)-H(12) | 126.1 | C(23)-C(24)-H(24) | 119.9 |
| C(13)-C(12)-C(11) | 107.8(2) | C(23)-C(24)-C(25) | 120.2(3) |
| C(13)-C(12)-H(12) | 126.1 | C(25)-C(24)-H(24) | 119.9 |
| N(3)-C(13)-C(12) | 109.4(3) | C(20)-C(25)-H(25) | 119.4 |
| N(3)-C(13)-C(35) | 119.7(3) | C(24)-C(25)-C(20) | 121.2(3) |
| C(12)-C(13)-C(35) | 130.9(3) | C(24)-C(25)-H(25) | 119.4 |
| C(15)-C(14)-C(3) | 119.8(3) | C(27)-C(26)-C(1) | 118.7(3) |
| C(15)-C(14)-C(19) | 118.2(3) | C(27)-C(26)-C(31) | 120.0(3) |

| | | | |
|---|---|---|---|
| C(31)-C(26)-C(1) | 121.1(3) | C(40)-C(35)-C(13) | 120.2(3) |
| C(26)-C(27)-C(32) | 121.3(3) | C(35)-C(36)-C(41) | 121.8(3) |
| C(28)-C(27)-C(26) | 119.4(3) | C(37)-C(36)-C(35) | 118.9(3) |
| C(28)-C(27)-C(32) | 119.2(3) | C(37)-C(36)-C(41) | 119.2(3) |
| C(27)-C(28)-H(28) | 119.3 | C(36)-C(37)-H(37) | 119.0 |
| C(29)-C(28)-C(27) | 121.4(3) | C(36)-C(37)-C(38) | 121.9(3) |
| C(29)-C(28)-H(28) | 119.3 | C(38)-C(37)-H(37) | 119.0 |
| C(28)-C(29)-C(33) | 120.1(3) | C(37)-C(38)-C(42) | 121.2(3) |
| C(30)-C(29)-C(28) | 118.4(3) | C(39)-C(38)-C(37) | 117.9(3) |
| C(30)-C(29)-C(33) | 121.6(3) | C(39)-C(38)-C(42) | 120.9(3) |
| C(29)-C(30)-H(30) | 118.9 | C(38)-C(39)-H(39) | 118.9 |
| C(29)-C(30)-C(31) | 122.1(3) | C(40)-C(39)-C(38) | 122.2(3) |
| C(31)-C(30)-H(30) | 118.9 | C(40)-C(39)-H(39) | 118.9 |
| C(26)-C(31)-C(34) | 121.0(3) | C(35)-C(40)-C(43) | 121.3(3) |
| C(30)-C(31)-C(26) | 118.5(3) | C(39)-C(40)-C(35) | 118.9(3) |
| C(30)-C(31)-C(34) | 120.4(3) | C(39)-C(40)-C(43) | 119.7(3) |
| C(27)-C(32)-H(32A) | 109.5 | C(36)-C(41)-H(41A) | 109.5 |
| C(27)-C(32)-H(32B) | 109.5 | C(36)-C(41)-H(41B) | 109.5 |
| C(27)-C(32)-H(32C) | 109.5 | C(36)-C(41)-H(41C) | 109.5 |
| H(32A)-C(32)-H(32B) | 109.5 | H(41A)-C(41)-H(41B) | 109.5 |
| H(32A)-C(32)-H(32C) | 109.5 | H(41A)-C(41)-H(41C) | 109.5 |
| H(32B)-C(32)-H(32C) | 109.5 | H(41B)-C(41)-H(41C) | 109.5 |
| C(29)-C(33)-H(33A) | 109.5 | C(38)-C(42)-H(42A) | 109.5 |
| C(29)-C(33)-H(33B) | 109.5 | C(38)-C(42)-H(42B) | 109.5 |
| C(29)-C(33)-H(33C) | 109.5 | C(38)-C(42)-H(42C) | 109.5 |
| H(33A)-C(33)-H(33B) | 109.5 | H(42A)-C(42)-H(42B) | 109.5 |
| H(33A)-C(33)-H(33C) | 109.5 | H(42A)-C(42)-H(42C) | 109.5 |
| H(33B)-C(33)-H(33C) | 109.5 | H(42B)-C(42)-H(42C) | 109.5 |
| C(31)-C(34)-H(34A) | 109.5 | C(40)-C(43)-H(43A) | 109.5 |
| C(31)-C(34)-H(34B) | 109.5 | C(40)-C(43)-H(43B) | 109.5 |
| C(31)-C(34)-H(34C) | 109.5 | C(40)-C(43)-H(43C) | 109.5 |
| H(34A)-C(34)-H(34B) | 109.5 | H(43A)-C(43)-H(43B) | 109.5 |
| H(34A)-C(34)-H(34C) | 109.5 | H(43A)-C(43)-H(43C) | 109.5 |
| H(34B)-C(34)-H(34C) | 109.5 | H(43B)-C(43)-H(43C) | 109.5 |
| C(36)-C(35)-C(13) | 119.6(3) | O(3)-C(44)-H(44A) | 110.8 |
| C(36)-C(35)-C(40) | 120.2(3) | O(3)-C(44)-H(44B) | 110.8 |

| | | | |
|---|---|---|---|
| O(3)-C(44)-C(45) | 104.8(2) | C(45)-C(46)-H(46B) | 110.9 |
| H(44A)-C(44)-H(44B) | 108.9 | C(45)-C(46)-C(47) | 104.1(3) |
| C(45)-C(44)-H(44A) | 110.8 | H(46A)-C(46)-H(46B) | 108.9 |
| C(45)-C(44)-H(44B) | 110.8 | C(47)-C(46)-H(46A) | 110.9 |
| C(44)-C(45)-H(45A) | 110.9 | C(47)-C(46)-H(46B) | 110.9 |
| C(44)-C(45)-H(45B) | 110.9 | O(3)-C(47)-C(46) | 105.5(2) |
| C(44)-C(45)-C(46) | 104.4(3) | O(3)-C(47)-H(47A) | 110.6 |
| H(45A)-C(45)-H(45B) | 108.9 | O(3)-C(47)-H(47B) | 110.6 |
| C(46)-C(45)-H(45A) | 110.9 | C(46)-C(47)-H(47A) | 110.6 |
| C(46)-C(45)-H(45B) | 110.9 | C(46)-C(47)-H(47B) | 110.6 |
| C(45)-C(46)-H(46A) | 110.9 | H(47A)-C(47)-H(47B) | 108.8 |

Table 4. Anisotropic displacement parameters ($Å^2 x 10^3$) for matbh01. The anisotropic displacement factor exponent takes the form: $-2\pi^2[ h^2 a^{*2}U_{11} + ... + 2 h k a^* b^* U_{12} ]$

| | $U_{11}$ | $U_{22}$ | $U_{33}$ | $U_{23}$ | $U_{13}$ | $U_{12}$ |
|---|---|---|---|---|---|---|
| U1 | 14(1) | 6(1) | 17(1) | 0(1) | 7(1) | 0(1) |
| O1 | 23(1) | 12(1) | 26(1) | 0(1) | 13(1) | 0(1) |
| O2 | 23(1) | 12(1) | 25(1) | 0(1) | 12(1) | 1(1) |
| O3 | 29(1) | 10(1) | 26(1) | 1(1) | 17(1) | 2(1) |
| N1 | 15(1) | 11(1) | 19(1) | -1(1) | 7(1) | 1(1) |
| N2 | 14(1) | 11(1) | 17(1) | -1(1) | 6(1) | 1(1) |
| N3 | 14(1) | 10(1) | 18(1) | -1(1) | 6(1) | 0(1) |
| C1 | 21(2) | 9(1) | 18(1) | 3(1) | 8(1) | 4(1) |
| C2 | 16(1) | 18(1) | 20(1) | 2(1) | 4(1) | 3(1) |
| C3 | 15(1) | 13(1) | 18(1) | -1(1) | 6(1) | 0(1) |
| C4 | 16(1) | 12(1) | 19(1) | 1(1) | 8(1) | 1(1) |
| C5 | 14(1) | 14(1) | 16(1) | 0(1) | 8(1) | 0(1) |
| C6 | 17(1) | 12(1) | 19(1) | -2(1) | 5(1) | -1(1) |
| C7 | 20(1) | 8(1) | 25(1) | -1(1) | 9(1) | 0(1) |
| C8 | 18(1) | 11(1) | 22(1) | 1(1) | 8(1) | 2(1) |
| C9 | 14(1) | 13(1) | 18(1) | 1(1) | 7(1) | 0(1) |
| C10 | 16(1) | 10(1) | 19(1) | -2(1) | 6(1) | -2(1) |
| C11 | 17(1) | 14(1) | 21(1) | 1(1) | 8(1) | 0(1) |
| C12 | 14(1) | 15(1) | 22(1) | -4(1) | 4(1) | -3(1) |
| C13 | 18(1) | 12(1) | 23(1) | -1(1) | 10(1) | -2(1) |
| C14 | 17(1) | 12(1) | 17(1) | 3(1) | 4(1) | 1(1) |
| C15 | 16(1) | 24(2) | 31(2) | -4(1) | 7(1) | 0(1) |
| C16 | 15(1) | 26(2) | 42(2) | -1(1) | 6(1) | -4(1) |
| C17 | 29(2) | 14(1) | 31(2) | -3(1) | 3(1) | -5(1) |
| C18 | 28(2) | 17(1) | 21(1) | -1(1) | 8(1) | 2(1) |
| C19 | 21(1) | 16(1) | 18(1) | 1(1) | 7(1) | 0(1) |
| C20 | 12(1) | 13(1) | 24(1) | 0(1) | 6(1) | -3(1) |
| C21 | 18(1) | 15(1) | 26(1) | -1(1) | 7(1) | -1(1) |
| C22 | 22(1) | 17(2) | 41(2) | -5(1) | 16(1) | -1(1) |
| C23 | 19(1) | 13(1) | 49(2) | 2(1) | 10(1) | 3(1) |
| C24 | 21(1) | 21(2) | 32(2) | 9(1) | 3(1) | 2(1) |

| | | | | | | |
|---|---|---|---|---|---|---|
| C25 | 20(1) | 18(2) | 25(1) | 3(1) | 6(1) | -1(1) |
| C26 | 20(1) | 10(1) | 17(1) | 1(1) | 5(1) | 3(1) |
| C27 | 25(1) | 14(1) | 21(1) | 0(1) | 9(1) | 2(1) |
| C28 | 32(2) | 16(2) | 24(1) | 1(1) | 12(1) | 0(1) |
| C29 | 39(2) | 11(1) | 22(1) | 1(1) | 10(1) | 4(1) |
| C30 | 37(2) | 16(2) | 27(2) | 1(1) | 14(1) | 8(1) |
| C31 | 27(2) | 16(1) | 25(1) | 2(1) | 12(1) | 5(1) |
| C32 | 41(2) | 23(2) | 34(2) | 4(1) | 25(2) | 6(1) |
| C33 | 73(3) | 13(2) | 38(2) | 3(1) | 28(2) | 9(2) |
| C34 | 48(2) | 23(2) | 51(2) | 6(2) | 36(2) | 10(2) |
| C35 | 16(1) | 13(1) | 19(1) | -3(1) | 5(1) | -1(1) |
| C36 | 16(1) | 17(1) | 23(1) | -5(1) | 8(1) | -5(1) |
| C37 | 22(1) | 14(1) | 28(1) | -1(1) | 12(1) | -2(1) |
| C38 | 20(1) | 14(1) | 30(2) | -5(1) | 10(1) | -3(1) |
| C39 | 23(1) | 19(2) | 26(1) | -4(1) | 14(1) | 0(1) |
| C40 | 18(1) | 15(1) | 23(1) | -1(1) | 9(1) | -1(1) |
| C41 | 30(2) | 21(2) | 31(2) | -6(1) | 18(1) | -4(1) |
| C42 | 36(2) | 16(2) | 43(2) | -7(1) | 19(2) | 0(1) |
| C43 | 48(2) | 21(2) | 36(2) | 0(1) | 28(2) | -2(2) |
| C44 | 39(2) | 19(2) | 30(2) | -3(1) | 25(1) | 2(1) |
| C45 | 58(2) | 17(2) | 50(2) | -6(1) | 38(2) | 4(2) |
| C46 | 55(2) | 13(2) | 46(2) | -1(1) | 31(2) | 1(1) |
| C47 | 32(2) | 8(1) | 28(1) | 2(1) | 17(1) | -1(1) |

Table 5. Hydrogen coordinates (x $10^4$) and isotropic displacement parameters (Å$^2$ x $10^3$) for matbh01.

| | x | y | z | U(eq) |
|---|---|---|---|---|
| H2 | 3246 | 6868 | 4485 | 23 |
| H6 | 4531 | 4174 | 5556 | 21 |
| H7 | 5310 | 3365 | 6269 | 22 |
| H8 | 6077 | 4149 | 7042 | 21 |
| H12 | 7232 | 6771 | 8460 | 23 |
| H15 | 2879 | 5230 | 4723 | 30 |
| H16 | 2427 | 3919 | 4150 | 36 |
| H17 | 2851 | 2930 | 3725 | 34 |
| H18 | 3740 | 3225 | 3890 | 28 |
| H19 | 4189 | 4546 | 4440 | 23 |
| H21 | 6856 | 4767 | 6884 | 25 |
| H22 | 7443 | 3523 | 7077 | 32 |
| H23 | 8005 | 3034 | 8163 | 35 |
| H24 | 7975 | 3794 | 9053 | 34 |
| H25 | 7388 | 5027 | 8863 | 27 |
| H28 | 4250 | 10108 | 4470 | 30 |
| H30 | 3376 | 10144 | 5497 | 32 |
| H32A | 4528 | 7712 | 4638 | 45 |
| H32B | 4838 | 8635 | 4633 | 45 |
| H32C | 4269 | 8380 | 4012 | 45 |
| H33A | 3972 | 11636 | 5171 | 61 |
| H33B | 3329 | 11446 | 4777 | 61 |
| H33C | 3695 | 11428 | 4393 | 61 |
| H34A | 3084 | 8056 | 5524 | 53 |
| H34B | 3305 | 8847 | 6070 | 53 |
| H34C | 3679 | 7975 | 6142 | 53 |
| H37 | 6673 | 10306 | 7572 | 26 |
| H39 | 6137 | 9612 | 8901 | 26 |
| H41A | 7194 | 8503 | 7401 | 38 |
| H41B | 6785 | 9221 | 6880 | 38 |

| | | | | |
|---|---|---|---|---|
| H41C | 6595 | 8187 | 6872 | 38 |
| H42A | 6791 | 11265 | 8800 | 47 |
| H42B | 6161 | 11202 | 8643 | 47 |
| H42C | 6321 | 11491 | 8072 | 47 |
| H43A | 5692 | 7673 | 8504 | 47 |
| H43B | 6130 | 8067 | 9204 | 47 |
| H43C | 6301 | 7271 | 8851 | 47 |
| H44A | 5083 | 9464 | 7645 | 31 |
| H44B | 4494 | 9102 | 7103 | 31 |
| H45A | 4652 | 10817 | 7305 | 44 |
| H45B | 4275 | 10452 | 6564 | 44 |
| H46A | 5393 | 10999 | 7118 | 42 |
| H46B | 4911 | 11249 | 6395 | 42 |
| H47A | 4979 | 9904 | 5961 | 26 |
| H47B | 5592 | 9914 | 6557 | 26 |

Table 6. Torsion angles [°] for matbh01.

| | | | |
|---|---|---|---|
| U1-O3-C44-C45 | -160.5(2) | C1-C26-C31-C34 | 5.0(5) |
| U1-O3-C47-C46 | -178.1(2) | C2-C1-C26-C27 | -94.8(4) |
| U1-N1-C1-C2 | -167.76(19) | C2-C1-C26-C31 | 80.6(4) |
| U1-N1-C1-C26 | 17.5(4) | C2-C3-C4-N1 | -0.8(3) |
| U1-N1-C4-C3 | 169.26(17) | C2-C3-C4-C5 | 177.4(3) |
| U1-N1-C4-C5 | -9.3(3) | C2-C3-C14-C15 | -49.4(4) |
| U1-N2-C5-C4 | 9.3(3) | C2-C3-C14-C19 | 126.9(3) |
| U1-N2-C5-C6 | -170.48(19) | C3-C4-C5-N2 | -178.8(3) |
| U1-N2-C9-C8 | 170.9(2) | C3-C4-C5-C6 | 0.9(5) |
| U1-N2-C9-C10 | -6.4(3) | C3-C14-C15-C16 | 177.1(3) |
| U1-N3-C10-C9 | -10.3(3) | C3-C14-C19-C18 | -177.8(3) |
| U1-N3-C10-C11 | 174.10(18) | C4-N1-C1-C2 | 1.3(3) |
| U1-N3-C13-C12 | -174.98(18) | C4-N1-C1-C26 | -173.4(3) |
| U1-N3-C13-C35 | 4.1(4) | C4-C3-C14-C15 | 132.2(3) |
| O3-C44-C45-C46 | -31.7(4) | C4-C3-C14-C19 | -51.5(4) |
| N1-C1-C2-C3 | -1.9(3) | C4-C5-C6-C7 | 179.3(3) |
| N1-C1-C26-C27 | 78.9(4) | C5-N2-C9-C8 | -1.1(4) |
| N1-C1-C26-C31 | -105.7(4) | C5-N2-C9-C10 | -178.5(2) |
| N1-C4-C5-N2 | -0.7(3) | C5-C6-C7-C8 | -0.3(4) |
| N1-C4-C5-C6 | 179.0(3) | C6-C7-C8-C9 | 0.8(4) |
| N2-C5-C6-C7 | -0.9(4) | C7-C8-C9-N2 | -0.1(4) |
| N2-C9-C10-N3 | 10.2(3) | C7-C8-C9-C10 | 176.9(3) |
| N2-C9-C10-C11 | -175.5(3) | C8-C9-C10-N3 | -166.9(3) |
| N3-C10-C11-C12 | 1.1(3) | C8-C9-C10-C11 | 7.3(5) |
| N3-C10-C11-C20 | -173.7(3) | C9-N2-C5-C4 | -178.6(2) |
| N3-C13-C35-C36 | -89.6(3) | C9-N2-C5-C6 | 1.6(4) |
| N3-C13-C35-C40 | 91.4(3) | C9-C10-C11-C12 | -173.4(3) |
| C1-N1-C4-C3 | -0.3(3) | C9-C10-C11-C20 | 11.8(5) |
| C1-N1-C4-C5 | -178.9(2) | C10-N3-C13-C12 | -0.8(3) |
| C1-C2-C3-C4 | 1.6(3) | C10-N3-C13-C35 | 178.3(3) |
| C1-C2-C3-C14 | -177.1(3) | C10-C11-C12-C13 | -1.6(3) |
| C1-C26-C27-C28 | 174.1(3) | C10-C11-C20-C21 | 43.7(4) |
| C1-C26-C27-C32 | -2.6(4) | C10-C11-C20-C25 | -140.3(3) |
| C1-C26-C31-C30 | -173.5(3) | C11-C12-C13-N3 | 1.5(3) |

| | | | |
|---|---|---|---|
| C11-C12-C13-C35 | -177.5(3) | C29-C30-C31-C34 | -179.0(3) |
| C11-C20-C21-C22 | 177.1(3) | C31-C26-C27-C28 | -1.3(4) |
| C11-C20-C25-C24 | -176.9(3) | C31-C26-C27-C32 | -178.0(3) |
| C12-C11-C20-C21 | -130.2(3) | C32-C27-C28-C29 | 176.3(3) |
| C12-C11-C20-C25 | 45.8(4) | C33-C29-C30-C31 | 177.7(3) |
| C12-C13-C35-C36 | 89.3(4) | C35-C36-C37-C38 | 1.1(4) |
| C12-C13-C35-C40 | -89.7(4) | C36-C35-C40-C39 | -1.8(4) |
| C13-N3-C10-C9 | 175.4(2) | C36-C35-C40-C43 | 179.8(3) |
| C13-N3-C10-C11 | -0.2(3) | C36-C37-C38-C39 | -1.6(4) |
| C13-C35-C36-C37 | -178.4(3) | C36-C37-C38-C42 | 176.6(3) |
| C13-C35-C36-C41 | -1.9(4) | C37-C38-C39-C40 | 0.3(4) |
| C13-C35-C40-C39 | 177.2(3) | C38-C39-C40-C35 | 1.3(4) |
| C13-C35-C40-C43 | -1.3(4) | C38-C39-C40-C43 | 179.8(3) |
| C14-C3-C4-N1 | 177.8(3) | C40-C35-C36-C37 | 0.6(4) |
| C14-C3-C4-C5 | -4.0(5) | C40-C35-C36-C41 | 177.1(3) |
| C14-C15-C16-C17 | -0.2(5) | C41-C36-C37-C38 | -175.5(3) |
| C15-C14-C19-C18 | -1.4(4) | C42-C38-C39-C40 | -177.8(3) |
| C15-C16-C17-C18 | 0.5(5) | C44-O3-C47-C46 | 4.5(3) |
| C16-C17-C18-C19 | -1.3(5) | C44-C45-C46-C47 | 34.4(4) |
| C17-C18-C19-C14 | 1.7(4) | C45-C46-C47-O3 | -24.0(4) |
| C19-C14-C15-C16 | 0.7(5) | C47-O3-C44-C45 | 16.8(3) |
| C20-C11-C12-C13 | 173.6(3) | | |
| C20-C21-C22-C23 | -0.6(4) | | |
| C21-C20-C25-C24 | -0.8(4) | | |
| C21-C22-C23-C24 | 0.0(5) | | |
| C22-C23-C24-C25 | 0.2(5) | | |
| C23-C24-C25-C20 | 0.2(5) | | |
| C25-C20-C21-C22 | 1.0(4) | | |
| C26-C1-C2-C3 | 172.5(3) | | |
| C26-C27-C28-C29 | -0.6(5) | | |
| C27-C26-C31-C30 | 1.8(4) | | |
| C27-C26-C31-C34 | -179.7(3) | | |
| C27-C28-C29-C30 | 1.8(5) | | |
| C27-C28-C29-C33 | -177.1(3) | | |
| C28-C29-C30-C31 | -1.2(5) | | |
| C29-C30-C31-C26 | -0.6(5) | | |

REFERENCE NUMBER: matbh07mo

CRYSTAL STRUCTURE REPORT

$C_{48} H_{37} Cl_4 N_3 O_3 U$

or

($^{Cl2Ph}PDP^{Ph}$)$UO_2$(thf) · $C_7H_8$

Report prepared for:

Dr. B. Hakey, Prof. E. Matson

March 04, 2022

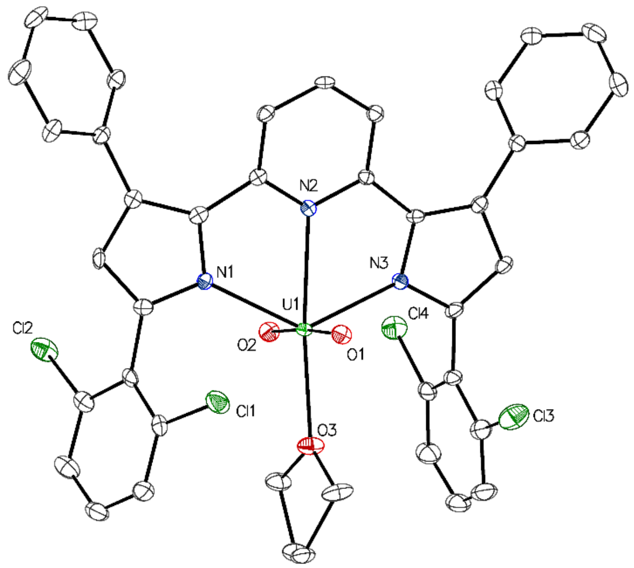



William W. Brennessel

X-ray Crystallographic Facility

Department of Chemistry, University of Rochester

120 Trustee Road

Rochester, NY 14627

## Data collection

A crystal (0.228 x 0.082 x 0.028 $mm^3$) was placed onto a thin glass optical fiber or a nylon loop and mounted on a Rigaku XtaLAB Synergy-S Dualflex diffractometer equipped with a HyPix-6000HE HPC area detector for data collection at 99.97(14) K. A preliminary set of cell constants and an orientation matrix were calculated from a small sampling of reflections.[1] A short pre-experiment was run, from which an optimal data collection strategy was determined. The full data collection was carried out using a PhotonJet (Mo) X-ray source with a frame time of 24.50 seconds and a detector distance of 34.0 mm. Series of frames were collected in 0.50º steps in $\omega$ at different $2\theta$, $\kappa$, and $\phi$ settings. After the intensity data were corrected for absorption, the final cell constants were calculated from the xyz centroids of 19389 strong reflections from the actual data collection after integration.[1] See Table 1 for additional crystal and refinement information.

## Structure solution and refinement

The structure was solved using SHELXT[2] and refined using SHELXL.[3] The space group $P2_1/n$ was determined based on systematic absences. Most or all non-hydrogen atoms were assigned from the solution. Full-matrix least squares / difference Fourier cycles were performed which located any remaining non-hydrogen atoms. All non-hydrogen atoms were refined with anisotropic displacement parameters. All hydrogen atoms were placed in ideal positions and refined as riding atoms with relative isotropic displacement parameters. The final full matrix least squares refinement converged to $R1 = 0.0467$ ($F^2$, $I > 2\sigma(I)$) and $wR2 = 0.0711$ ($F^2$, all data).

## Structure description

The structure is the one suggested. The asymmetric unit contains one uranium complex and one toluene solvent molecule of crystallization, both in general positions.

Structure manipulation and figure generation were performed using Olex2.[4] Unless noted otherwise all structural diagrams containing anisotropic displacement ellipsoids are drawn at the 50 % probability level.

Data collection, structure solution, and structure refinement were conducted at the X-ray Crystallographic Facility, B04 Hutchison Hall, Department of Chemistry, University of Rochester. The instrument was purchased with funding from NSF MRI program grant CHE-1725028. All publications arising from this report MUST either 1) include William W. Brennessel as a coauthor or 2) acknowledge William W. Brennessel and the X-ray Crystallographic Facility of the Department of Chemistry at the University of Rochester.

[1] *CrysAlisPro*, version 171.41.120a; Rigaku Corporation: Oxford, UK, 2021.

[2] Sheldrick, G. M. *SHELXT*, version 2018/2; *Acta. Crystallogr.* **2015**, *A71*, 3-8.

[3] Sheldrick, G. M. *SHELXL*, version 2018/3; *Acta. Crystallogr.* **2015**, *C71*, 3-8.

[4] Dolomanov, O. V.; Bourhis, L. J.; Gildea, R. J.; Howard, J. A. K.; Puschmann, H. *Olex2*, version 1.5; *J. Appl. Cryst.* **2009**, *42*, 339-341.

Some equations of interest:

$$R_{int} = \Sigma \, |F_o^2 - \langle F_o^2 \rangle| \, / \, \Sigma |F_o^2|$$

$$R1 = \Sigma ||F_o| - |F_c|| \, / \, \Sigma |F_o|$$

$$wR2 = [\Sigma[w(F_o^2 - F_c^2)^2] \, / \, \Sigma \, [w(F_o^2)^2]]^{1/2}$$

$$\text{where } w = 1 \, / \, [\sigma^2 (F_o^2) + (aP)^2 + bP] \text{ and}$$

$$P = 1/3 \max (0, F_o^2) + 2/3 \, F_c^2$$

$$\text{GOF} = S = [\Sigma[w(F_o^2 - F_c^2)^2] \, / \, (m-n)]^{1/2}$$

where $m$ = number of reflections and $n$ = number of parameters

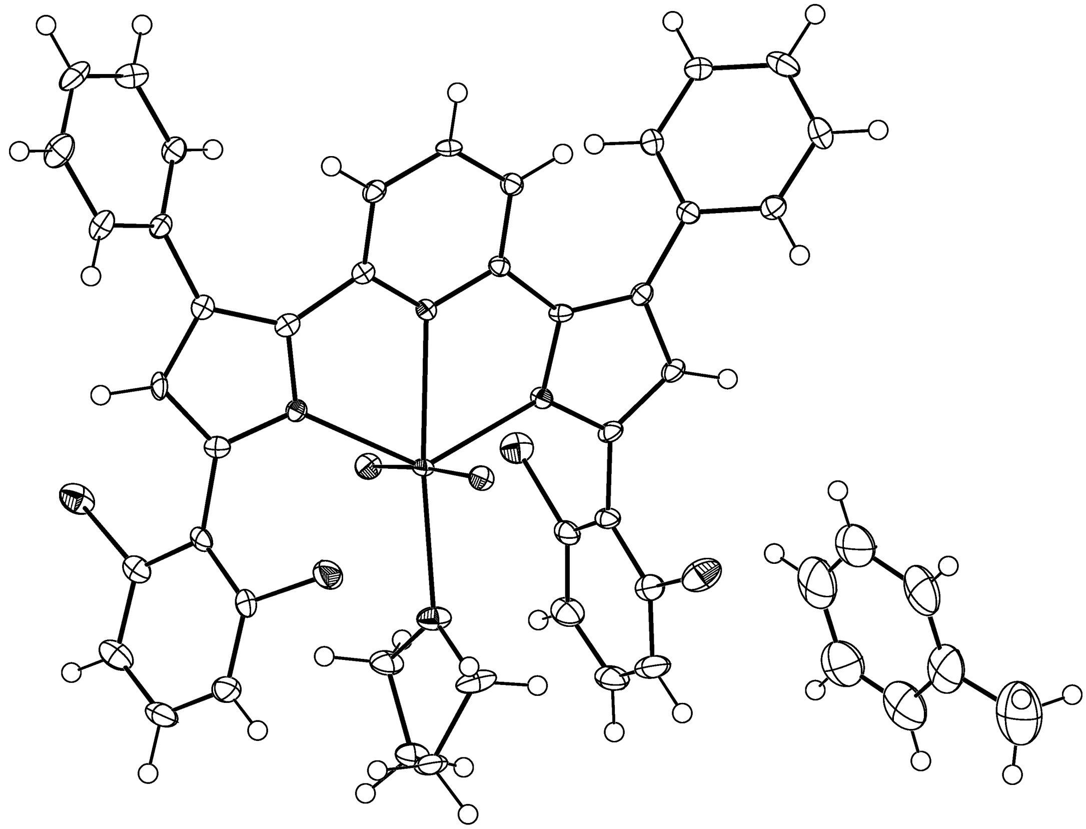

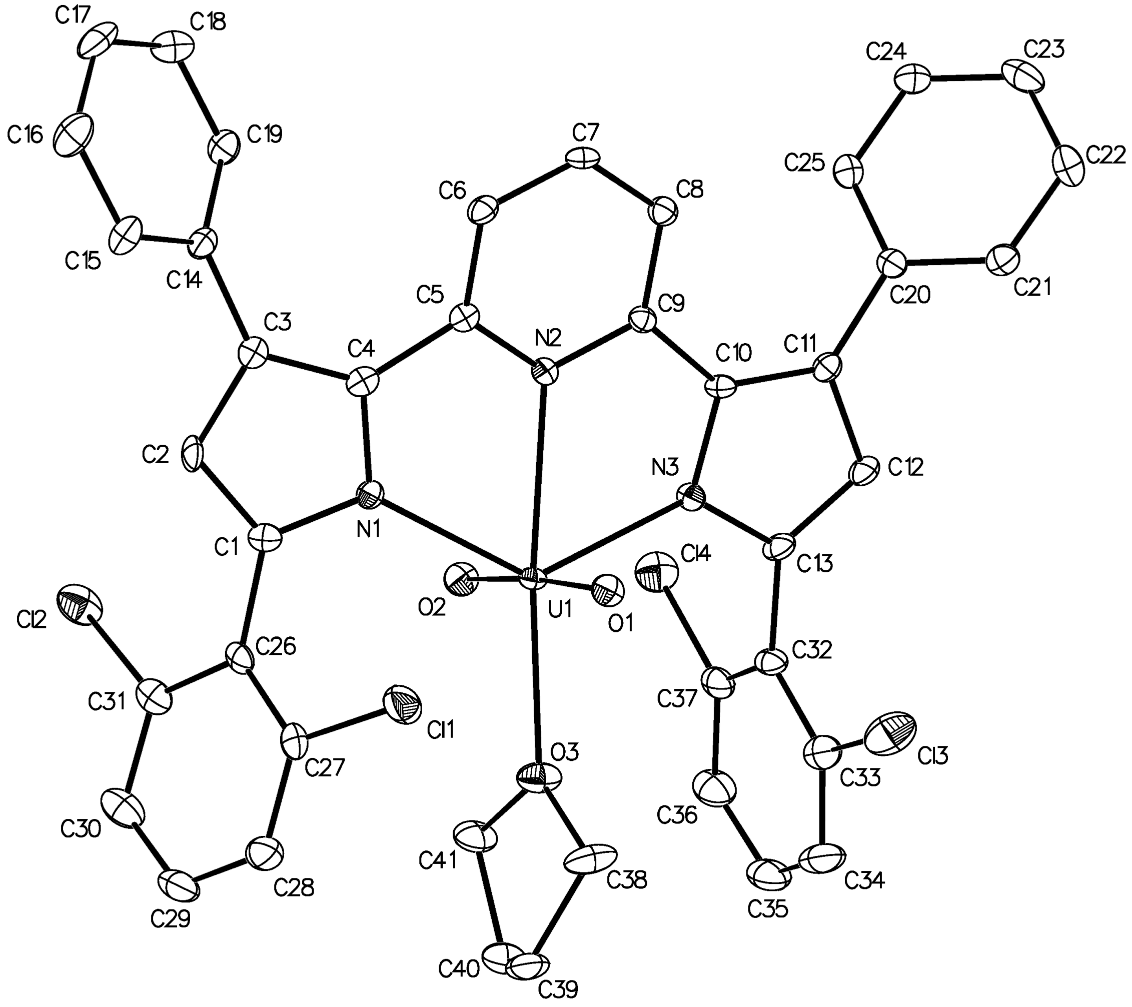
C17
C18
C16
C19
C15
C14
C3
C4
C5
C6
C7
C8
C9
N2
C10
C11
C20
C21
C22
C23
C24
C25
C2
C1
N1
N3
C12
C13
Cl4
O2
U1
O1
Cl2
C26
C31
C27
Cl1
C32
C37
C33
Cl3
O3
C30
C28
C29
C41
C38
C40
C39
C36
C35
C34

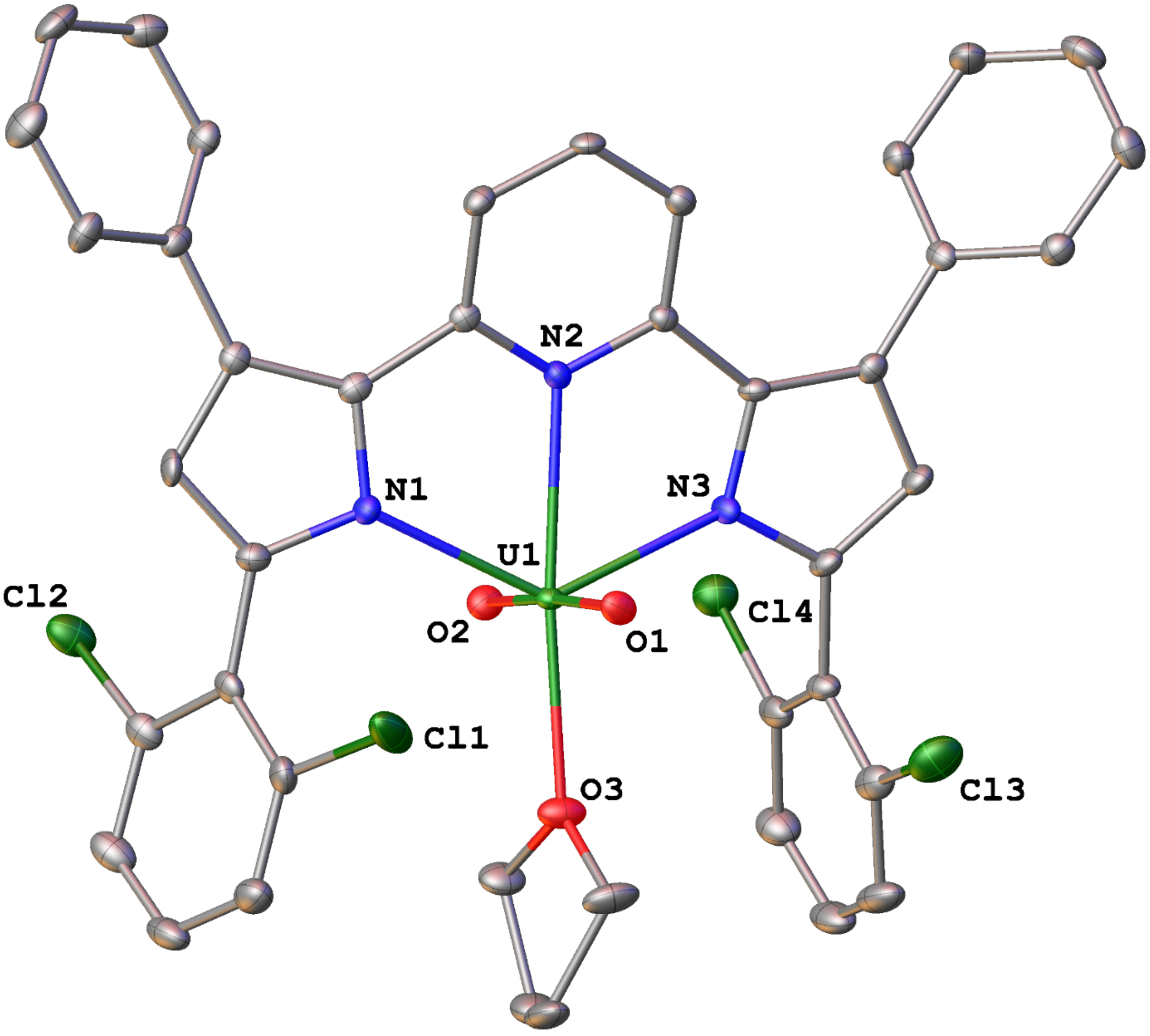
N2
N1
N3
U1
Cl2
Cl4
O2
O1
Cl1
O3
Cl3

Table 1. Crystal data and structure refinement for matbh07mo.

| | | |
|---|---|---|
| Identification code | matbh07mo | |
| Empirical formula | C48 H37 Cl4 N3 O3 U | |
| Formula weight | 1083.63 | |
| Temperature | 99.97(14) K | |
| Wavelength | 0.71073 Å | |
| Crystal system | monoclinic | |
| Space group | $P2_1/n$ | |
| Unit cell dimensions | *a* = 15.7732(6) Å | $\alpha$ = 90° |
| | *b* = 12.7863(4) Å | $\beta$ = 96.753(4)° |
| | *c* = 20.9071(10) Å | $\gamma$ = 90° |
| Volume | 4187.3(3) $Å^3$ | |
| *Z* | 4 | |
| Density (calculated) | 1.719 $Mg/m^3$ | |
| Absorption coefficient | 4.179 $mm^{-1}$ | |
| *F*(000) | 2120 | |
| Crystal color, morphology | red, plate | |
| Crystal size | 0.228 x 0.082 x 0.028 $mm^3$ | |
| Theta range for data collection | 2.211 to 33.110° | |
| Index ranges | $-20 \leq h \leq 23$, $-19 \leq k \leq 19$, $-29 \leq l \leq 29$ | |
| Reflections collected | 62800 | |
| Independent reflections | 13698 [*R*(int) = 0.0598] | |
| Observed reflections | 9708 | |
| Completeness to theta = 29.575° | 99.9% | |
| Absorption correction | Multi-scan | |
| Max. and min. transmission | 1.00000 and 0.31093 | |
| Refinement method | Full-matrix least-squares on $F^2$ | |
| Data / restraints / parameters | 13698 / 0 / 533 | |
| Goodness-of-fit on $F^2$ | 1.020 | |
| Final *R* indices [*I*>2sigma(*I*)] | *R*1 = 0.0467, *wR*2 = 0.0644 | |
| *R* indices (all data) | *R*1 = 0.0822, *wR*2 = 0.0711 | |
| Largest diff. peak and hole | 1.958 and -1.304 e.$Å^{-3}$ | |

Table 2. Atomic coordinates (x $10^4$) and equivalent isotropic displacement parameters ($Å^2$x $10^3$ ) for matbh07mo. $U_{eq}$ is defined as one third of the trace of the orthogonalized $U_{ij}$ tensor.

| | x | y | z | $U_{eq}$ |
|---|---|---|---|---|
| U1 | 5513(1) | 2463(1) | 6306(1) | 11(1) |
| Cl1 | 6786(1) | 793(1) | 5495(1) | 33(1) |
| Cl2 | 8589(1) | 1774(1) | 7806(1) | 26(1) |
| Cl3 | 2713(1) | 1485(1) | 5537(1) | 32(1) |
| Cl4 | 3850(1) | 3214(1) | 7845(1) | 24(1) |
| O1 | 5231(2) | 2254(2) | 5471(1) | 16(1) |
| O2 | 5743(2) | 2563(2) | 7153(1) | 16(1) |
| O3 | 5149(2) | 676(2) | 6515(1) | 21(1) |
| N1 | 6967(2) | 2913(2) | 6253(2) | 13(1) |
| N2 | 5750(2) | 4347(2) | 6052(2) | 12(1) |
| N3 | 4292(2) | 3534(2) | 6320(2) | 12(1) |
| C1 | 7703(2) | 2367(2) | 6454(2) | 14(1) |
| C2 | 8404(2) | 3003(3) | 6433(2) | 15(1) |
| C3 | 8106(2) | 4000(3) | 6216(2) | 12(1) |
| C4 | 7215(2) | 3913(3) | 6113(2) | 12(1) |
| C5 | 6533(2) | 4651(2) | 5911(2) | 10(1) |
| C6 | 6630(2) | 5601(2) | 5595(2) | 13(1) |
| C7 | 5928(2) | 6237(2) | 5450(2) | 13(1) |
| C8 | 5137(2) | 5943(2) | 5615(2) | 13(1) |
| C9 | 5058(2) | 4982(2) | 5917(2) | 11(1) |
| C10 | 4264(2) | 4561(2) | 6095(2) | 12(1) |
| C11 | 3425(2) | 4933(2) | 6054(2) | 12(1) |
| C12 | 2926(2) | 4092(3) | 6238(2) | 15(1) |
| C13 | 3474(2) | 3267(2) | 6405(2) | 15(1) |
| C14 | 8675(2) | 4903(3) | 6147(2) | 14(1) |
| C15 | 9435(2) | 4744(3) | 5872(2) | 18(1) |
| C16 | 9999(3) | 5566(3) | 5814(2) | 24(1) |
| C17 | 9816(3) | 6556(3) | 6025(2) | 24(1) |
| C18 | 9075(3) | 6726(3) | 6304(2) | 21(1) |
| C19 | 8513(2) | 5906(3) | 6366(2) | 16(1) |
| C20 | 3067(2) | 5988(3) | 5909(2) | 13(1) |

| | | | | |
|---|---|---|---|---|
| C21 | 2342(2) | 6107(3) | 5463(2) | 16(1) |
| C22 | 1948(2) | 7082(3) | 5363(2) | 20(1) |
| C23 | 2272(3) | 7934(3) | 5717(2) | 24(1) |
| C24 | 2994(3) | 7823(3) | 6161(2) | 22(1) |
| C25 | 3385(2) | 6860(3) | 6251(2) | 17(1) |
| C26 | 7696(2) | 1251(3) | 6648(2) | 14(1) |
| C27 | 7324(2) | 458(3) | 6242(2) | 19(1) |
| C28 | 7381(2) | -600(3) | 6399(2) | 24(1) |
| C29 | 7806(3) | -900(3) | 6980(2) | 24(1) |
| C30 | 8177(3) | -160(3) | 7403(2) | 23(1) |
| C31 | 8114(2) | 893(3) | 7234(2) | 17(1) |
| C32 | 3280(2) | 2270(2) | 6718(2) | 14(1) |
| C33 | 2900(2) | 1421(3) | 6373(2) | 21(1) |
| C34 | 2656(3) | 522(3) | 6681(2) | 30(1) |
| C35 | 2803(3) | 458(3) | 7339(2) | 29(1) |
| C36 | 3193(3) | 1269(3) | 7708(2) | 25(1) |
| C37 | 3416(2) | 2164(3) | 7386(2) | 20(1) |
| C38 | 4834(3) | -125(3) | 6042(2) | 27(1) |
| C39 | 5096(3) | -1152(3) | 6372(2) | 30(1) |
| C40 | 5003(3) | -914(3) | 7077(2) | 29(1) |
| C41 | 5341(3) | 188(3) | 7156(2) | 23(1) |
| C42 | -827(4) | 355(6) | 5434(4) | 73(2) |
| C43 | -196(3) | 870(4) | 5911(3) | 47(1) |
| C44 | -55(4) | 479(5) | 6532(3) | 51(2) |
| C45 | 466(4) | 978(4) | 6983(3) | 52(2) |
| C46 | 882(4) | 1894(5) | 6850(4) | 56(2) |
| C47 | 775(3) | 2285(4) | 6238(4) | 63(2) |
| C48 | 216(3) | 1746(4) | 5749(3) | 48(1) |

Table 3. Bond lengths [Å] and angles [°] for matbh07mo.

| | | | |
|---|---|---|---|
| U(1)-O(1) | 1.771(3) | C(11)-C(12) | 1.413(5) |
| U(1)-O(2) | 1.771(2) | C(11)-C(20) | 1.480(4) |
| U(1)-O(3) | 2.408(2) | C(12)-H(12) | 0.9500 |
| U(1)-N(1) | 2.381(3) | C(12)-C(13) | 1.383(5) |
| U(1)-N(2) | 2.504(3) | C(13)-C(32) | 1.482(4) |
| U(1)-N(3) | 2.367(3) | C(14)-C(15) | 1.403(5) |
| Cl(1)-C(27) | 1.741(4) | C(14)-C(19) | 1.396(5) |
| Cl(2)-C(31) | 1.746(4) | C(15)-H(15) | 0.9500 |
| Cl(3)-C(33) | 1.740(5) | C(15)-C(16) | 1.392(5) |
| Cl(4)-C(37) | 1.744(4) | C(16)-H(16) | 0.9500 |
| O(3)-C(38) | 1.470(5) | C(16)-C(17) | 1.382(5) |
| O(3)-C(41) | 1.477(5) | C(17)-H(17) | 0.9500 |
| N(1)-C(1) | 1.377(4) | C(17)-C(18) | 1.384(5) |
| N(1)-C(4) | 1.379(4) | C(18)-H(18) | 0.9500 |
| N(2)-C(5) | 1.361(4) | C(18)-C(19) | 1.390(5) |
| N(2)-C(9) | 1.362(4) | C(19)-H(19) | 0.9500 |
| N(3)-C(10) | 1.394(4) | C(20)-C(21) | 1.397(5) |
| N(3)-C(13) | 1.365(4) | C(20)-C(25) | 1.387(5) |
| C(1)-C(2) | 1.378(5) | C(21)-H(21) | 0.9500 |
| C(1)-C(26) | 1.484(5) | C(21)-C(22) | 1.397(5) |
| C(2)-H(2) | 0.9500 | C(22)-H(22) | 0.9500 |
| C(2)-C(3) | 1.415(5) | C(22)-C(23) | 1.381(6) |
| C(3)-C(4) | 1.400(5) | C(23)-H(23) | 0.9500 |
| C(3)-C(14) | 1.480(5) | C(23)-C(24) | 1.388(6) |
| C(4)-C(5) | 1.456(5) | C(24)-H(24) | 0.9500 |
| C(5)-C(6) | 1.398(4) | C(24)-C(25) | 1.380(5) |
| C(6)-H(6) | 0.9500 | C(25)-H(25) | 0.9500 |
| C(6)-C(7) | 1.380(5) | C(26)-C(27) | 1.406(5) |
| C(7)-H(7) | 0.9500 | C(26)-C(31) | 1.398(5) |
| C(7)-C(8) | 1.386(5) | C(27)-C(28) | 1.392(5) |
| C(8)-H(8) | 0.9500 | C(28)-H(28) | 0.9500 |
| C(8)-C(9) | 1.393(4) | C(28)-C(29) | 1.371(6) |
| C(9)-C(10) | 1.451(5) | C(29)-H(29) | 0.9500 |
| C(10)-C(11) | 1.399(5) | C(29)-C(30) | 1.378(6) |

| | | | |
|---|---|---|---|
| C(30)-H(30) | 0.9500 | C(48)-H(48) | 0.9500 |
| C(30)-C(31) | 1.394(5) | O(1)-U(1)-O(3) | 89.91(10) |
| C(32)-C(33) | 1.399(5) | O(1)-U(1)-N(1) | 96.99(11) |
| C(32)-C(37) | 1.393(6) | O(1)-U(1)-N(2) | 87.84(10) |
| C(33)-C(34) | 1.394(5) | O(1)-U(1)-N(3) | 89.42(11) |
| C(34)-H(34) | 0.9500 | O(2)-U(1)-O(1) | 174.67(11) |
| C(34)-C(35) | 1.371(7) | O(2)-U(1)-O(3) | 84.95(10) |
| C(35)-H(35) | 0.9500 | O(2)-U(1)-N(1) | 86.75(11) |
| C(35)-C(36) | 1.392(6) | O(2)-U(1)-N(2) | 97.17(11) |
| C(36)-H(36) | 0.9500 | O(2)-U(1)-N(3) | 91.00(11) |
| C(36)-C(37) | 1.394(5) | O(3)-U(1)-N(2) | 174.76(10) |
| C(38)-H(38A) | 0.9900 | N(1)-U(1)-O(3) | 119.30(9) |
| C(38)-H(38B) | 0.9900 | N(1)-U(1)-N(2) | 65.71(9) |
| C(38)-C(39) | 1.518(6) | N(3)-U(1)-O(3) | 109.59(9) |
| C(39)-H(39A) | 0.9900 | N(3)-U(1)-N(1) | 130.61(9) |
| C(39)-H(39B) | 0.9900 | N(3)-U(1)-N(2) | 65.67(9) |
| C(39)-C(40) | 1.527(6) | C(38)-O(3)-U(1) | 127.4(2) |
| C(40)-H(40A) | 0.9900 | C(38)-O(3)-C(41) | 109.5(3) |
| C(40)-H(40B) | 0.9900 | C(41)-O(3)-U(1) | 122.5(2) |
| C(40)-C(41) | 1.509(5) | C(1)-N(1)-U(1) | 130.1(2) |
| C(41)-H(41A) | 0.9900 | C(1)-N(1)-C(4) | 106.6(3) |
| C(41)-H(41B) | 0.9900 | C(4)-N(1)-U(1) | 122.2(2) |
| C(42)-H(42A) | 0.9800 | C(5)-N(2)-U(1) | 118.9(2) |
| C(42)-H(42B) | 0.9800 | C(5)-N(2)-C(9) | 120.6(3) |
| C(42)-H(42C) | 0.9800 | C(9)-N(2)-U(1) | 118.8(2) |
| C(42)-C(43) | 1.478(9) | C(10)-N(3)-U(1) | 122.3(2) |
| C(43)-C(44) | 1.386(8) | C(13)-N(3)-U(1) | 129.8(2) |
| C(43)-C(48) | 1.357(8) | C(13)-N(3)-C(10) | 106.7(3) |
| C(44)-H(44) | 0.9500 | N(1)-C(1)-C(2) | 109.9(3) |
| C(44)-C(45) | 1.337(8) | N(1)-C(1)-C(26) | 122.6(3) |
| C(45)-H(45) | 0.9500 | C(2)-C(1)-C(26) | 127.4(3) |
| C(45)-C(46) | 1.387(8) | C(1)-C(2)-H(2) | 126.1 |
| C(46)-H(46) | 0.9500 | C(1)-C(2)-C(3) | 107.7(3) |
| C(46)-C(47) | 1.365(9) | C(3)-C(2)-H(2) | 126.1 |
| C(47)-H(47) | 0.9500 | C(2)-C(3)-C(14) | 123.5(3) |
| C(47)-C(48) | 1.444(9) | C(4)-C(3)-C(2) | 105.6(3) |

| | | | |
|---|---|---|---|
| C(4)-C(3)-C(14) | 131.0(3) | C(16)-C(15)-H(15) | 119.6 |
| N(1)-C(4)-C(3) | 110.2(3) | C(15)-C(16)-H(16) | 119.9 |
| N(1)-C(4)-C(5) | 116.4(3) | C(17)-C(16)-C(15) | 120.2(4) |
| C(3)-C(4)-C(5) | 133.4(3) | C(17)-C(16)-H(16) | 119.9 |
| N(2)-C(5)-C(4) | 114.2(3) | C(16)-C(17)-H(17) | 120.1 |
| N(2)-C(5)-C(6) | 120.3(3) | C(16)-C(17)-C(18) | 119.8(3) |
| C(6)-C(5)-C(4) | 125.5(3) | C(18)-C(17)-H(17) | 120.1 |
| C(5)-C(6)-H(6) | 120.4 | C(17)-C(18)-H(18) | 119.9 |
| C(7)-C(6)-C(5) | 119.1(3) | C(17)-C(18)-C(19) | 120.1(3) |
| C(7)-C(6)-H(6) | 120.4 | C(19)-C(18)-H(18) | 119.9 |
| C(6)-C(7)-H(7) | 119.7 | C(14)-C(19)-H(19) | 119.4 |
| C(6)-C(7)-C(8) | 120.5(3) | C(18)-C(19)-C(14) | 121.3(3) |
| C(8)-C(7)-H(7) | 119.7 | C(18)-C(19)-H(19) | 119.4 |
| C(7)-C(8)-H(8) | 120.5 | C(21)-C(20)-C(11) | 119.9(3) |
| C(7)-C(8)-C(9) | 118.9(3) | C(25)-C(20)-C(11) | 121.4(3) |
| C(9)-C(8)-H(8) | 120.5 | C(25)-C(20)-C(21) | 118.4(3) |
| N(2)-C(9)-C(8) | 120.5(3) | C(20)-C(21)-H(21) | 119.6 |
| N(2)-C(9)-C(10) | 114.8(3) | C(20)-C(21)-C(22) | 120.8(3) |
| C(8)-C(9)-C(10) | 124.6(3) | C(22)-C(21)-H(21) | 119.6 |
| N(3)-C(10)-C(9) | 116.3(3) | C(21)-C(22)-H(22) | 120.2 |
| N(3)-C(10)-C(11) | 109.4(3) | C(23)-C(22)-C(21) | 119.6(4) |
| C(11)-C(10)-C(9) | 134.2(3) | C(23)-C(22)-H(22) | 120.2 |
| C(10)-C(11)-C(12) | 106.1(3) | C(22)-C(23)-H(23) | 120.0 |
| C(10)-C(11)-C(20) | 131.2(3) | C(22)-C(23)-C(24) | 120.0(3) |
| C(12)-C(11)-C(20) | 122.5(3) | C(24)-C(23)-H(23) | 120.0 |
| C(11)-C(12)-H(12) | 126.4 | C(23)-C(24)-H(24) | 119.9 |
| C(13)-C(12)-C(11) | 107.3(3) | C(25)-C(24)-C(23) | 120.1(4) |
| C(13)-C(12)-H(12) | 126.4 | C(25)-C(24)-H(24) | 119.9 |
| N(3)-C(13)-C(12) | 110.4(3) | C(20)-C(25)-H(25) | 119.4 |
| N(3)-C(13)-C(32) | 121.3(3) | C(24)-C(25)-C(20) | 121.1(4) |
| C(12)-C(13)-C(32) | 127.8(3) | C(24)-C(25)-H(25) | 119.4 |
| C(15)-C(14)-C(3) | 119.0(3) | C(27)-C(26)-C(1) | 123.0(3) |
| C(19)-C(14)-C(3) | 123.2(3) | C(31)-C(26)-C(1) | 122.4(3) |
| C(19)-C(14)-C(15) | 117.7(3) | C(31)-C(26)-C(27) | 114.4(3) |
| C(14)-C(15)-H(15) | 119.6 | C(26)-C(27)-Cl(1) | 119.3(3) |
| C(16)-C(15)-C(14) | 120.9(3) | C(28)-C(27)-Cl(1) | 117.5(3) |

| | |
|---|---|
| C(28)-C(27)-C(26) | 123.2(4) |
| C(27)-C(28)-H(28) | 120.2 |
| C(29)-C(28)-C(27) | 119.5(4) |
| C(29)-C(28)-H(28) | 120.2 |
| C(28)-C(29)-H(29) | 119.9 |
| C(28)-C(29)-C(30) | 120.2(4) |
| C(30)-C(29)-H(29) | 119.9 |
| C(29)-C(30)-H(30) | 120.4 |
| C(29)-C(30)-C(31) | 119.2(4) |
| C(31)-C(30)-H(30) | 120.4 |
| C(26)-C(31)-Cl(2) | 120.6(3) |
| C(30)-C(31)-Cl(2) | 115.9(3) |
| C(30)-C(31)-C(26) | 123.4(4) |
| C(33)-C(32)-C(13) | 122.5(4) |
| C(37)-C(32)-C(13) | 120.7(3) |
| C(37)-C(32)-C(33) | 116.6(3) |
| C(32)-C(33)-Cl(3) | 119.6(3) |
| C(34)-C(33)-Cl(3) | 118.6(3) |
| C(34)-C(33)-C(32) | 121.8(4) |
| C(33)-C(34)-H(34) | 120.3 |
| C(35)-C(34)-C(33) | 119.3(4) |
| C(35)-C(34)-H(34) | 120.3 |
| C(34)-C(35)-H(35) | 119.2 |
| C(34)-C(35)-C(36) | 121.5(4) |
| C(36)-C(35)-H(35) | 119.2 |
| C(35)-C(36)-H(36) | 121.2 |
| C(35)-C(36)-C(37) | 117.7(4) |
| C(37)-C(36)-H(36) | 121.2 |
| C(32)-C(37)-Cl(4) | 118.8(3) |
| C(32)-C(37)-C(36) | 123.1(4) |
| C(36)-C(37)-Cl(4) | 118.1(3) |
| O(3)-C(38)-H(38A) | 110.9 |
| O(3)-C(38)-H(38B) | 110.9 |
| O(3)-C(38)-C(39) | 104.1(4) |
| H(38A)-C(38)-H(38B) | 109.0 |
| C(39)-C(38)-H(38A) | 110.9 |
| C(39)-C(38)-H(38B) | 110.9 |
| C(38)-C(39)-H(39A) | 111.3 |
| C(38)-C(39)-H(39B) | 111.3 |
| C(38)-C(39)-C(40) | 102.4(3) |
| H(39A)-C(39)-H(39B) | 109.2 |
| C(40)-C(39)-H(39A) | 111.3 |
| C(40)-C(39)-H(39B) | 111.3 |
| C(39)-C(40)-H(40A) | 111.2 |
| C(39)-C(40)-H(40B) | 111.2 |
| H(40A)-C(40)-H(40B) | 109.1 |
| C(41)-C(40)-C(39) | 102.7(3) |
| C(41)-C(40)-H(40A) | 111.2 |
| C(41)-C(40)-H(40B) | 111.2 |
| O(3)-C(41)-C(40) | 105.2(3) |
| O(3)-C(41)-H(41A) | 110.7 |
| O(3)-C(41)-H(41B) | 110.7 |
| C(40)-C(41)-H(41A) | 110.7 |
| C(40)-C(41)-H(41B) | 110.7 |
| H(41A)-C(41)-H(41B) | 108.8 |
| H(42A)-C(42)-H(42B) | 109.5 |
| H(42A)-C(42)-H(42C) | 109.5 |
| H(42B)-C(42)-H(42C) | 109.5 |
| C(43)-C(42)-H(42A) | 109.5 |
| C(43)-C(42)-H(42B) | 109.5 |
| C(43)-C(42)-H(42C) | 109.5 |
| C(44)-C(43)-C(42) | 119.5(6) |
| C(48)-C(43)-C(42) | 120.3(6) |
| C(48)-C(43)-C(44) | 120.2(6) |
| C(43)-C(44)-H(44) | 119.8 |
| C(45)-C(44)-C(43) | 120.4(6) |
| C(45)-C(44)-H(44) | 119.8 |
| C(44)-C(45)-H(45) | 119.1 |
| C(44)-C(45)-C(46) | 121.9(7) |
| C(46)-C(45)-H(45) | 119.1 |
| C(45)-C(46)-H(46) | 120.4 |
| C(47)-C(46)-C(45) | 119.2(6) |

| | | | |
|---|---|---|---|
| C(47)-C(46)-H(46) | 120.4 | C(43)-C(48)-C(47) | 119.2(6) |
| C(46)-C(47)-H(47) | 120.4 | C(43)-C(48)-H(48) | 120.4 |
| C(46)-C(47)-C(48) | 119.1(6) | C(47)-C(48)-H(48) | 120.4 |
| C(48)-C(47)-H(47) | 120.4 | | |

Table 4. Anisotropic displacement parameters ($Å^2$x $10^3$) for matbh07mo. The anisotropic displacement factor exponent takes the form: $-2\pi^2[ h^2 a^{*2}U_{11} + ... + 2 h k a^* b^* U_{12} ]$

| | $U_{11}$ | $U_{22}$ | $U_{33}$ | $U_{23}$ | $U_{13}$ | $U_{12}$ |
|---|---|---|---|---|---|---|
| U1 | 12(1) | 8(1) | 15(1) | 2(1) | 1(1) | 0(1) |
| Cl1 | 36(1) | 18(1) | 39(1) | -6(1) | -18(1) | 5(1) |
| Cl2 | 37(1) | 23(1) | 16(1) | 1(1) | 0(1) | 5(1) |
| Cl3 | 37(1) | 29(1) | 30(1) | -11(1) | 7(1) | -8(1) |
| Cl4 | 28(1) | 24(1) | 21(1) | 1(1) | 4(1) | -1(1) |
| O1 | 20(1) | 14(1) | 11(1) | -2(1) | -4(1) | 0(1) |
| O2 | 18(1) | 17(1) | 13(1) | 2(1) | 0(1) | -2(1) |
| O3 | 33(2) | 14(1) | 15(2) | 1(1) | 3(1) | -4(1) |
| N1 | 9(1) | 13(1) | 16(2) | 1(1) | 3(1) | -1(1) |
| N2 | 11(1) | 11(1) | 13(2) | 0(1) | 0(1) | 1(1) |
| N3 | 10(1) | 12(1) | 15(2) | 2(1) | 6(1) | 2(1) |
| C1 | 17(2) | 12(2) | 15(2) | 1(1) | 2(1) | 0(1) |
| C2 | 6(2) | 20(2) | 18(2) | 2(1) | 2(1) | 1(1) |
| C3 | 13(2) | 15(2) | 8(2) | -1(1) | 2(1) | -1(1) |
| C4 | 15(2) | 14(2) | 8(2) | 0(1) | 3(1) | -2(1) |
| C5 | 12(2) | 13(1) | 8(2) | -1(1) | 3(1) | -1(1) |
| C6 | 13(2) | 14(2) | 13(2) | 2(1) | 3(1) | -4(1) |
| C7 | 16(2) | 8(1) | 14(2) | 2(1) | 3(1) | -2(1) |
| C8 | 13(2) | 13(2) | 12(2) | 0(1) | 1(1) | 1(1) |
| C9 | 12(2) | 11(1) | 10(2) | 0(1) | 1(1) | 1(1) |
| C10 | 14(2) | 8(1) | 13(2) | 1(1) | 4(1) | -1(1) |
| C11 | 12(2) | 13(1) | 12(2) | -1(1) | 1(1) | -2(1) |
| C12 | 11(2) | 15(2) | 20(2) | 1(1) | 4(1) | -3(1) |
| C13 | 12(2) | 12(2) | 21(2) | -1(1) | 6(1) | -3(1) |
| C14 | 14(2) | 16(2) | 12(2) | 1(1) | 0(1) | -3(1) |
| C15 | 14(2) | 23(2) | 18(2) | -5(2) | 2(2) | -5(1) |
| C16 | 18(2) | 30(2) | 27(2) | -4(2) | 9(2) | -7(2) |
| C17 | 21(2) | 25(2) | 24(2) | -1(2) | 1(2) | -14(2) |
| C18 | 27(2) | 16(2) | 21(2) | -1(2) | 5(2) | -4(2) |
| C19 | 13(2) | 18(2) | 16(2) | -1(1) | 4(1) | -1(1) |
| C20 | 11(2) | 14(2) | 14(2) | 2(1) | 6(1) | 1(1) |

| | | | | | | |
|---|---|---|---|---|---|---|
| C21 | 16(2) | 15(2) | 18(2) | -2(1) | 4(2) | -2(1) |
| C22 | 18(2) | 24(2) | 16(2) | 2(2) | -1(2) | 6(1) |
| C23 | 27(2) | 15(2) | 29(3) | 4(2) | 8(2) | 7(2) |
| C24 | 20(2) | 12(2) | 33(3) | -3(2) | -1(2) | -1(1) |
| C25 | 13(2) | 16(2) | 21(2) | 0(1) | 0(2) | 1(1) |
| C26 | 11(2) | 15(2) | 16(2) | 1(1) | 5(1) | 5(1) |
| C27 | 11(2) | 19(2) | 26(2) | 0(2) | -1(2) | 3(1) |
| C28 | 18(2) | 17(2) | 37(3) | -1(2) | 6(2) | 1(1) |
| C29 | 23(2) | 16(2) | 36(3) | 9(2) | 11(2) | 5(2) |
| C30 | 28(2) | 22(2) | 21(2) | 7(2) | 11(2) | 8(2) |
| C31 | 18(2) | 18(2) | 15(2) | 1(1) | 7(2) | 4(1) |
| C32 | 13(2) | 11(2) | 20(2) | 3(1) | 7(1) | -1(1) |
| C33 | 17(2) | 17(2) | 30(2) | -3(2) | 10(2) | 0(1) |
| C34 | 24(2) | 12(2) | 54(3) | -4(2) | 14(2) | -3(2) |
| C35 | 24(2) | 17(2) | 48(3) | 12(2) | 13(2) | 1(2) |
| C36 | 25(2) | 23(2) | 28(3) | 8(2) | 10(2) | 2(2) |
| C37 | 14(2) | 14(2) | 32(3) | 4(1) | 8(2) | 3(1) |
| C38 | 40(3) | 16(2) | 24(3) | -4(2) | 6(2) | -11(2) |
| C39 | 22(2) | 10(2) | 57(3) | -2(2) | 8(2) | -3(1) |
| C40 | 29(2) | 18(2) | 39(3) | 10(2) | 2(2) | -2(2) |
| C41 | 31(2) | 17(2) | 21(2) | 7(2) | 3(2) | -2(2) |
| C42 | 50(4) | 97(5) | 73(5) | -14(4) | 14(4) | 18(4) |
| C43 | 28(3) | 58(3) | 58(4) | -10(3) | 16(3) | 9(2) |
| C44 | 42(3) | 56(3) | 60(4) | 2(3) | 25(3) | 19(3) |
| C45 | 42(3) | 49(3) | 70(5) | -6(3) | 18(3) | 12(3) |
| C46 | 36(3) | 61(4) | 72(5) | -15(3) | 9(3) | 18(3) |
| C47 | 28(3) | 45(3) | 119(6) | 11(3) | 27(3) | 10(2) |
| C48 | 30(3) | 61(3) | 56(4) | 11(3) | 15(3) | 18(3) |

Table 5. Hydrogen coordinates (x $10^4$) and isotropic displacement parameters (Å$^2$ x $10^3$) for matbh07mo.

| | x | y | z | U(eq) |
|---|---|---|---|---|
| H2 | 8984 | 2808 | 6544 | 18 |
| H6 | 7173 | 5804 | 5483 | 16 |
| H7 | 5987 | 6883 | 5235 | 15 |
| H8 | 4656 | 6389 | 5525 | 15 |
| H12 | 2325 | 4093 | 6245 | 18 |
| H15 | 9566 | 4067 | 5725 | 22 |
| H16 | 10512 | 5446 | 5628 | 29 |
| H17 | 10198 | 7118 | 5978 | 28 |
| H18 | 8951 | 7404 | 6454 | 25 |
| H19 | 8008 | 6030 | 6561 | 19 |
| H21 | 2113 | 5518 | 5225 | 19 |
| H22 | 1461 | 7158 | 5053 | 24 |
| H23 | 2002 | 8596 | 5657 | 28 |
| H24 | 3218 | 8410 | 6402 | 27 |
| H25 | 3882 | 6794 | 6553 | 20 |
| H28 | 7126 | -1110 | 6106 | 29 |
| H29 | 7846 | -1621 | 7090 | 29 |
| H30 | 8471 | -366 | 7806 | 27 |
| H34 | 2391 | -41 | 6437 | 36 |
| H35 | 2634 | -154 | 7548 | 35 |
| H36 | 3304 | 1214 | 8163 | 30 |
| H38A | 5100 | -45 | 5639 | 32 |
| H38B | 4206 | -83 | 5940 | 32 |
| H39A | 5693 | -1336 | 6316 | 36 |
| H39B | 4714 | -1729 | 6206 | 36 |
| H40A | 4398 | -952 | 7159 | 34 |
| H40B | 5345 | -1404 | 7369 | 34 |
| H41A | 5054 | 571 | 7482 | 28 |
| H41B | 5964 | 185 | 7293 | 28 |
| H42A | -861 | -390 | 5539 | 109 |

| | | | | |
|---|---|---|---|---|
| H42B | -650 | 432 | 5002 | 109 |
| H42C | -1387 | 680 | 5445 | 109 |
| H44 | -331 | -147 | 6639 | 61 |
| H45 | 554 | 697 | 7406 | 63 |
| H46 | 1238 | 2245 | 7180 | 68 |
| H47 | 1064 | 2904 | 6135 | 75 |
| H48 | 138 | 2001 | 5319 | 58 |

Table 6. Torsion angles [°] for matbh07mo.

| | | | |
|---|---|---|---|
| U1-O3-C38-C39 | 154.8(2) | C1-C26-C27-C28 | 174.3(3) |
| U1-O3-C41-C40 | 179.4(2) | C1-C26-C31-Cl2 | 5.9(5) |
| U1-N1-C1-C2 | -169.3(2) | C1-C26-C31-C30 | -174.6(3) |
| U1-N1-C1-C26 | 11.7(5) | C2-C1-C26-C27 | -122.1(4) |
| U1-N1-C4-C3 | 170.4(2) | C2-C1-C26-C31 | 53.2(5) |
| U1-N1-C4-C5 | -8.2(4) | C2-C3-C4-N1 | -0.4(4) |
| U1-N2-C5-C4 | -17.9(4) | C2-C3-C4-C5 | 177.8(4) |
| U1-N2-C5-C6 | 161.9(3) | C2-C3-C14-C15 | 43.0(5) |
| U1-N2-C9-C8 | -163.3(3) | C2-C3-C14-C19 | -134.3(4) |
| U1-N2-C9-C10 | 15.1(4) | C3-C4-C5-N2 | -161.3(4) |
| U1-N3-C10-C9 | -6.7(4) | C3-C4-C5-C6 | 19.0(6) |
| U1-N3-C10-C11 | 169.4(2) | C3-C14-C15-C16 | -178.3(4) |
| U1-N3-C13-C12 | -166.7(2) | C3-C14-C19-C18 | 178.5(4) |
| U1-N3-C13-C32 | 20.8(5) | C4-N1-C1-C2 | -0.9(4) |
| Cl1-C27-C28-C29 | 179.4(3) | C4-N1-C1-C26 | -179.8(3) |
| Cl3-C33-C34-C35 | 179.8(3) | C4-C3-C14-C15 | -138.9(4) |
| O3-C38-C39-C40 | 34.9(4) | C4-C3-C14-C19 | 43.8(6) |
| N1-C1-C2-C3 | 0.6(4) | C4-C5-C6-C7 | -178.1(3) |
| N1-C1-C26-C27 | 56.6(5) | C5-N2-C9-C8 | 1.8(5) |
| N1-C1-C26-C31 | -128.1(4) | C5-N2-C9-C10 | -179.8(3) |
| N1-C4-C5-N2 | 16.9(5) | C5-C6-C7-C8 | 0.2(5) |
| N1-C4-C5-C6 | -162.9(3) | C6-C7-C8-C9 | -1.5(5) |
| N2-C5-C6-C7 | 2.2(5) | C7-C8-C9-N2 | 0.5(5) |
| N2-C9-C10-N3 | -5.9(5) | C7-C8-C9-C10 | -177.7(3) |
| N2-C9-C10-C11 | 179.2(4) | C8-C9-C10-N3 | 172.4(3) |
| N3-C10-C11-C12 | -2.1(4) | C8-C9-C10-C11 | -2.4(7) |
| N3-C10-C11-C20 | 173.0(4) | C9-N2-C5-C4 | 177.0(3) |
| N3-C13-C32-C33 | -106.2(4) | C9-N2-C5-C6 | -3.2(5) |
| N3-C13-C32-C37 | 78.4(5) | C9-C10-C11-C12 | 172.9(4) |
| C1-N1-C4-C3 | 0.8(4) | C9-C10-C11-C20 | -12.0(7) |
| C1-N1-C4-C5 | -177.8(3) | C10-N3-C13-C12 | 0.2(4) |
| C1-C2-C3-C4 | -0.1(4) | C10-N3-C13-C32 | -172.3(3) |
| C1-C2-C3-C14 | 178.4(3) | C10-C11-C12-C13 | 2.2(4) |
| C1-C26-C27-Cl1 | -4.1(5) | C10-C11-C20-C21 | 132.8(4) |

| | |
|---|---|
| C10-C11-C20-C25 | -53.3(6) |
| C11-C12-C13-N3 | -1.6(4) |
| C11-C12-C13-C32 | 170.3(4) |
| C11-C20-C21-C22 | 174.1(3) |
| C11-C20-C25-C24 | -173.2(3) |
| C12-C11-C20-C21 | -52.7(5) |
| C12-C11-C20-C25 | 121.1(4) |
| C12-C13-C32-C33 | 82.7(5) |
| C12-C13-C32-C37 | -92.7(5) |
| C13-N3-C10-C9 | -174.8(3) |
| C13-N3-C10-C11 | 1.2(4) |
| C13-C32-C33-Cl3 | 4.7(5) |
| C13-C32-C33-C34 | -174.5(3) |
| C13-C32-C37-Cl4 | -2.0(5) |
| C13-C32-C37-C36 | 175.7(3) |
| C14-C3-C4-N1 | -178.8(3) |
| C14-C3-C4-C5 | -0.5(7) |
| C14-C15-C16-C17 | -0.2(6) |
| C15-C14-C19-C18 | 1.2(6) |
| C15-C16-C17-C18 | 1.0(7) |
| C16-C17-C18-C19 | -0.7(6) |
| C17-C18-C19-C14 | -0.5(6) |
| C19-C14-C15-C16 | -0.9(6) |
| C20-C11-C12-C13 | -173.4(3) |
| C20-C21-C22-C23 | -1.1(6) |
| C21-C20-C25-C24 | 0.7(5) |
| C21-C22-C23-C24 | 1.2(6) |
| C22-C23-C24-C25 | -0.4(6) |
| C23-C24-C25-C20 | -0.6(6) |
| C25-C20-C21-C22 | 0.1(5) |
| C26-C1-C2-C3 | 179.5(3) |
| C26-C27-C28-C29 | 0.9(6) |
| C27-C26-C31-Cl2 | -178.4(3) |
| C27-C26-C31-C30 | 1.1(5) |
| C27-C28-C29-C30 | -0.1(6) |
| C28-C29-C30-C31 | -0.1(6) |
| C29-C30-C31-Cl2 | 179.1(3) |
| C29-C30-C31-C26 | -0.4(6) |
| C31-C26-C27-Cl1 | -179.8(3) |
| C31-C26-C27-C28 | -1.4(5) |
| C32-C33-C34-C35 | -1.0(6) |
| C33-C32-C37-Cl4 | -177.7(3) |
| C33-C32-C37-C36 | 0.1(5) |
| C33-C34-C35-C36 | -0.3(6) |
| C34-C35-C36-C37 | 1.4(6) |
| C35-C36-C37-Cl4 | 176.5(3) |
| C35-C36-C37-C32 | -1.3(6) |
| C37-C32-C33-Cl3 | -179.7(3) |
| C37-C32-C33-C34 | 1.1(5) |
| C38-O3-C41-C40 | -8.5(4) |
| C38-C39-C40-C41 | -40.0(4) |
| C39-C40-C41-O3 | 30.0(4) |
| C41-O3-C38-C39 | -16.8(4) |
| C42-C43-C44-C45 | -175.1(5) |
| C42-C43-C48-C47 | 174.8(5) |
| C43-C44-C45-C46 | 0.0(8) |
| C44-C43-C48-C47 | -2.4(7) |
| C44-C45-C46-C47 | -1.7(8) |
| C45-C46-C47-C48 | 1.3(8) |
| C46-C47-C48-C43 | 0.7(7) |
| C48-C43-C44-C45 | 2.1(7) |
