# Supplementary material for: Synthesis and Characterization of Pyridine Dipyrrolide Uranyl Complexes
Source: Inorg Chem. 2022 Apr 14;61(16):6182–92. doi: 10.1021/acs.inorgchem.2c00348 (PMC9044449; doi:10.1021/acs.inorgchem.2c00348)
Supplement: Supplementary file 2 — ic2c00348_si_002.pdf [file ic2c00348_si_002.pdf]

Computational Supporting Information

Synthesis and Characterization of Pyridine Dipyrrolide Uranyl Complexes

Brett M. Hakey[†], Dylan C. Leary[‡], Lauren Lopez[†], William W. Brennessel[†], Carsten Milsmann[‡*], Ellen M. Matson[†*]

[†] Department of Chemistry, University of Rochester, Rochester, New York 14627, United States

[‡]C. Eugene Bennett Department of Chemistry, West Virginia University, Morgantown, West Virginia 26506, USA

*Corresponding author

Contents

1. Computational Details 3

1.1 Input Files, Optimized Coordinates, and Vibrational Frequencies 11

1.2 References..18

1. Computational Details

All calculations were performed using the ORCA quantum chemical program package v5.0.1.[1,2] Geometry optimizations used the PBE functional[3] and were accelerated using the resolution of identity (RI) approximation.[4,5] Scalar-relativistic effects were included *via* the zeroth-order regular approximation (ZORA)[6] using relativistically recontracted triple-ζ quality basis sets, ZORA-def2-TZVP,[7] on nitrogen and oxygen atoms and SARC-ZORA-TZVP[8] for uranium. All other atoms were handled with the recontracted split-valence ZORA-def2-SVP basis set.[7] Noncovalent interactions were considered *via* atom-pairwise dispersion corrections with Becke – Johnson (D3BJ) damping.[9,10] TD-DFT calculations used the B3LYP density functional[11] and were accelerated using the RIJCOSX approximation.[12,13] Relativistic effects were included using the Douglas-Kroll-Hess (DKH) Hamiltonian with DKH-specific basis sets analogous to those used in the geometry optimizations. The Tamm-Dancoff approximation was not used and the effects of spin-orbit coupling (SOC) were probed using a spin-orbit mean field (SOMF) approach.[14] All solvation effects were handled using the conductor-like polarizable continuum model (C-PCM) and a gaussian charge scheme.[15]

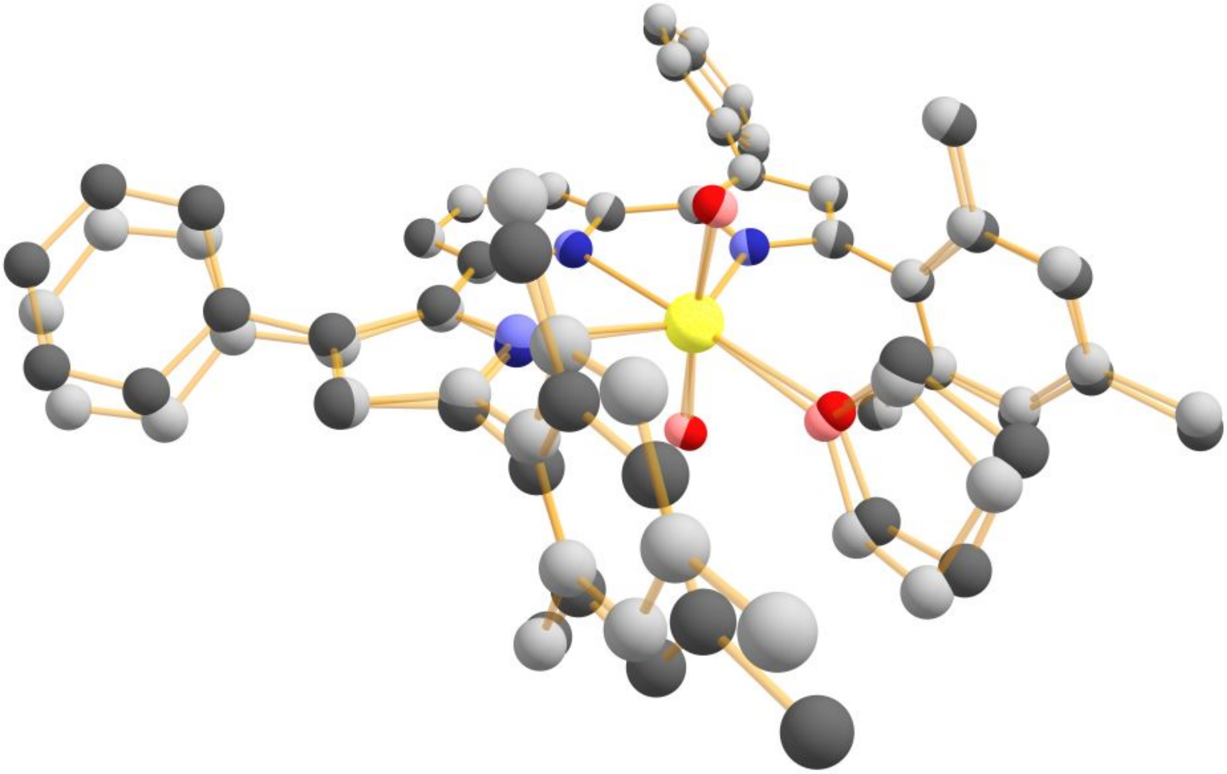

Figure S1. Overlay of the crystal structure (dark) and PBE-optimized structure (light). The similarity between the two structures is visually evident and quantitatively demonstrated with a relatively small root-mean-square deviation (RMSD) of 0.51 Å.

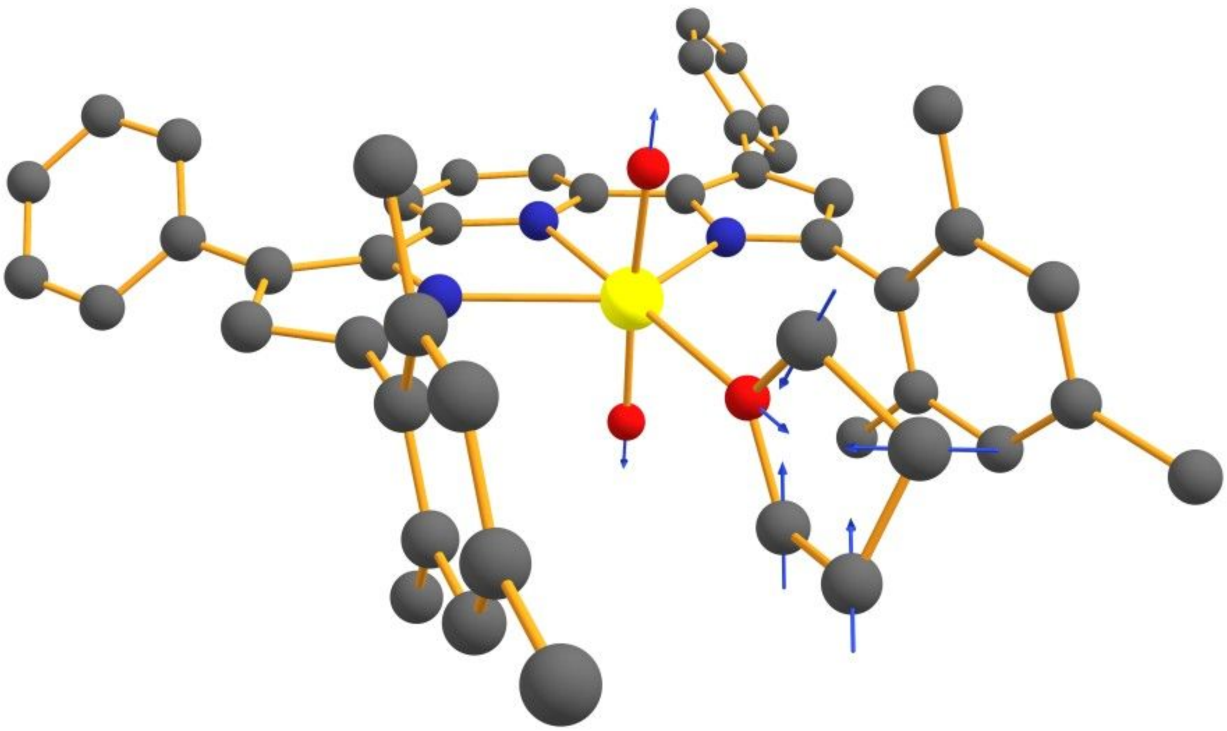

Figure S2. Vectorial representation of the symmetric uranyl stretch calculated at 852 cm^{-1}.

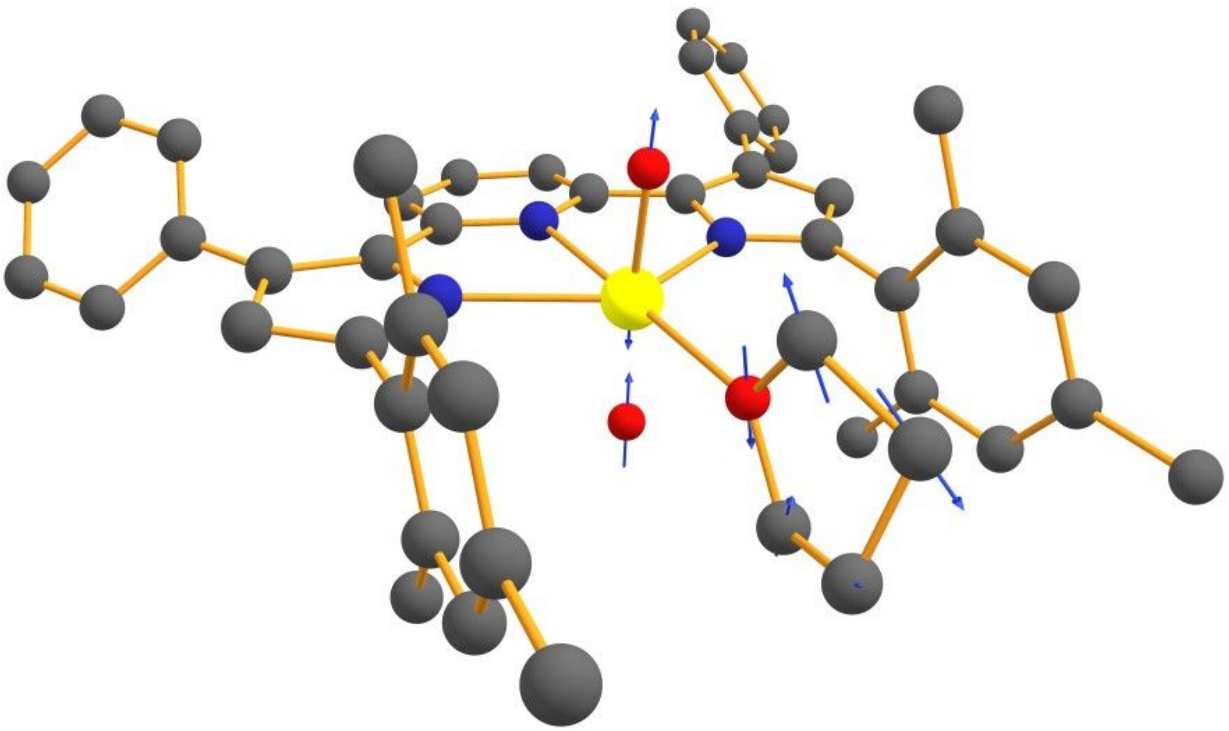

Figure S3. Vectorial representation of the asymmetric uranyl stretch calculated at 893 cm^{-1}. One of the uranyl bonds is omitted to see its associated atomic displacement vectors more clearly.

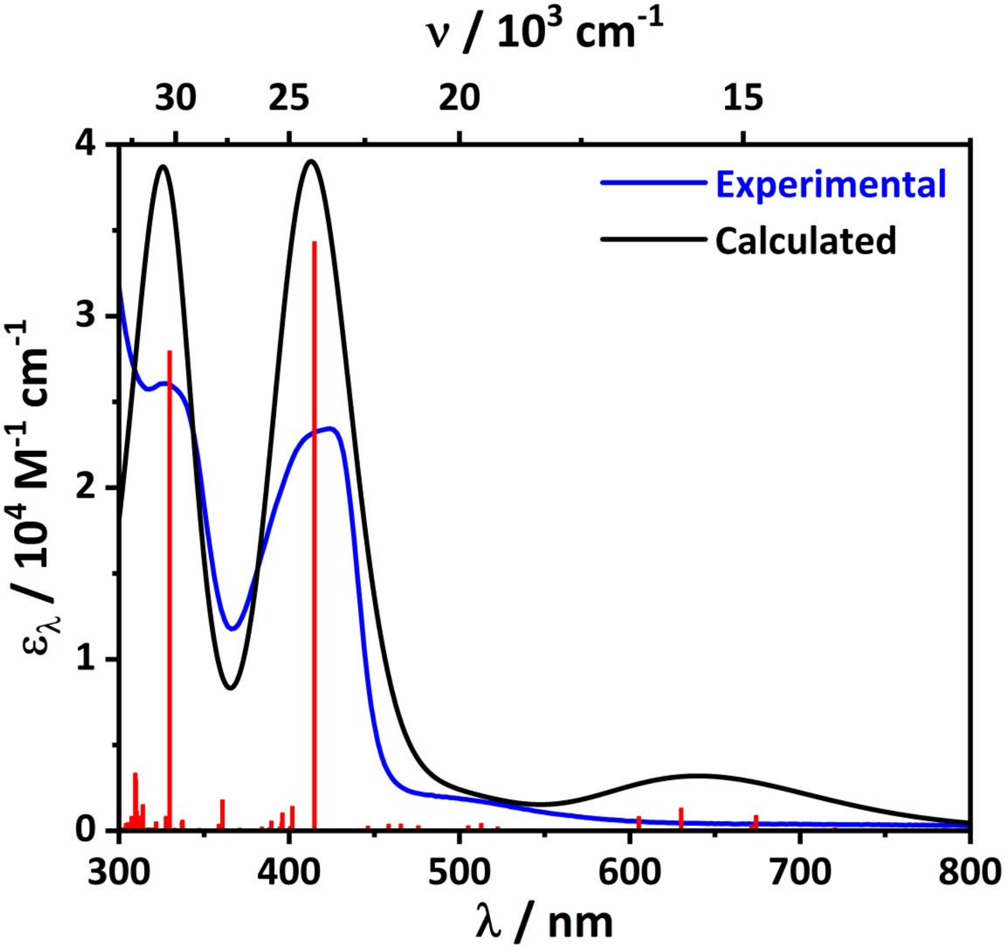

Figure S4. Comparison of experimental (blue) and SOC-TD-DFT calculated (black) absorption spectra. The effects of SOC are most prominent for the lowest energy transitions which are not shown here (λ > 1200 nm).

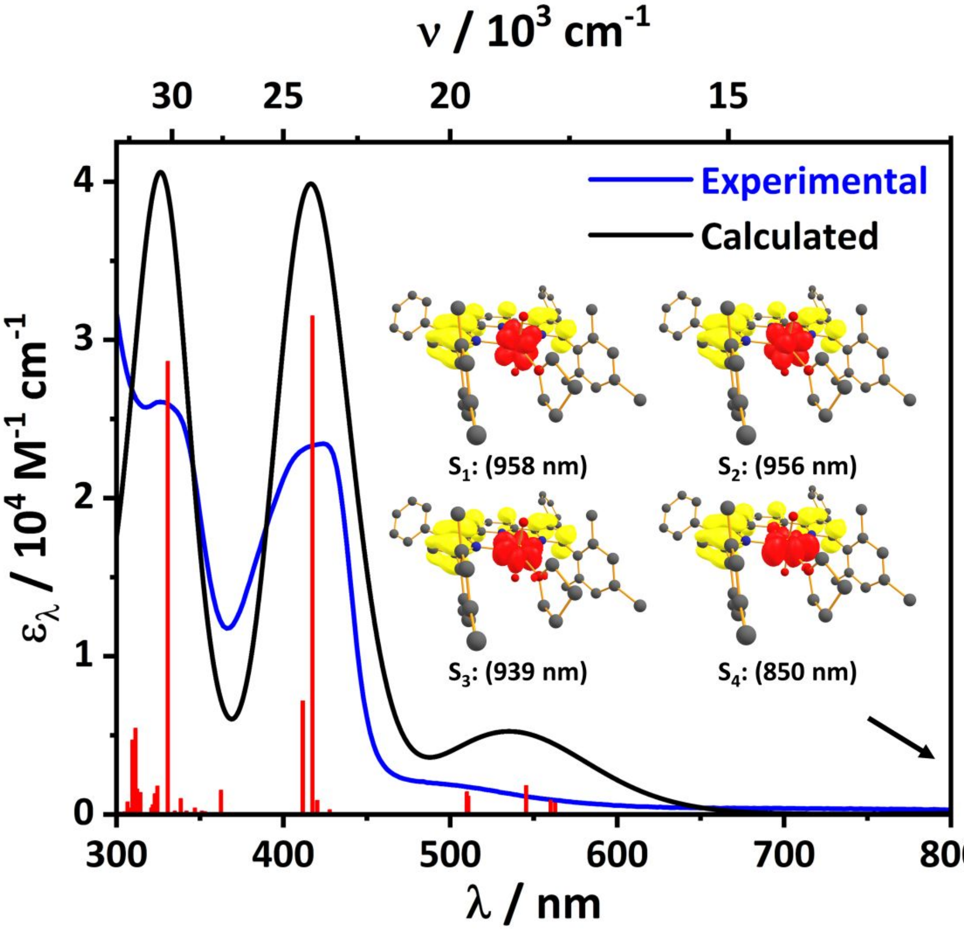

Figure S5. Comparison of experimental (blue) and TD-DFT calculated (black) absorption spectra. The lowest energy excited states (λ > 850 nm) are represented as unrelaxed difference densities (red = gain of electron density, yellow = loss of electron density).

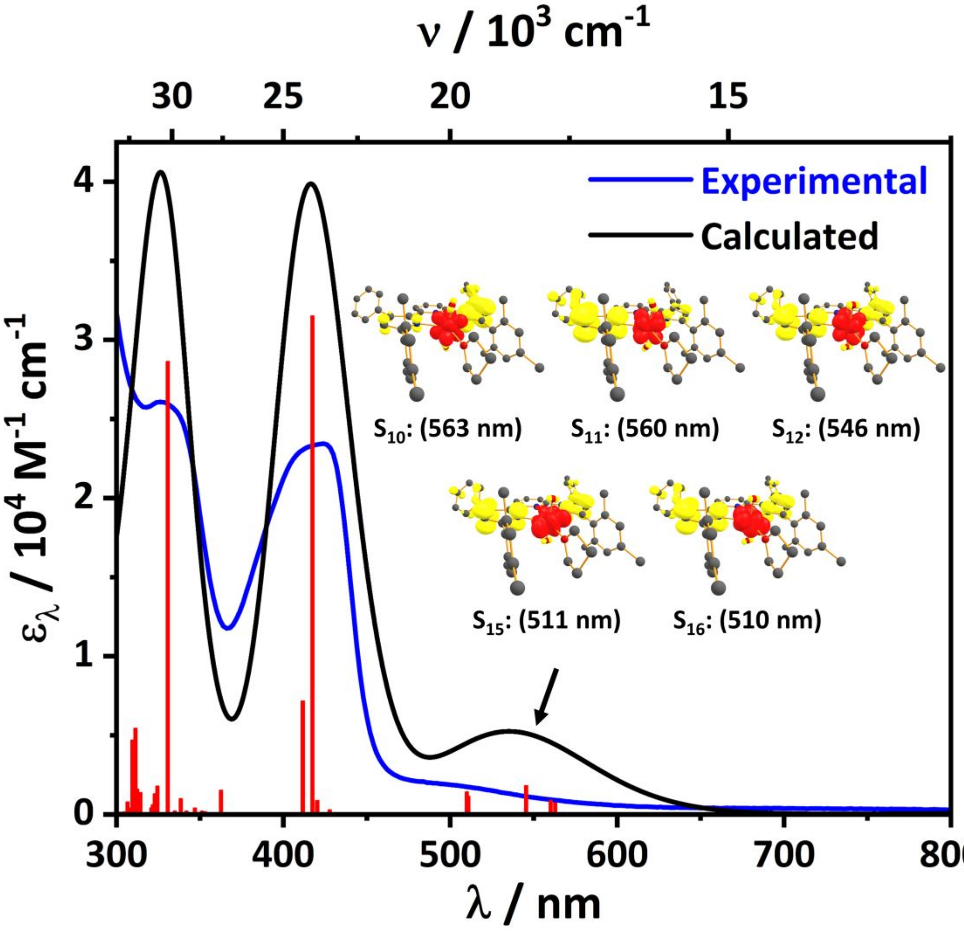

Figure S6. Comparison of experimental (blue) and TD-DFT calculated (black) absorption spectra. The transitions responsible for the calculated band centered at ca. 540 nm are shown as unrelaxed difference densities (red = gain of electron density, yellow = loss of electron density).

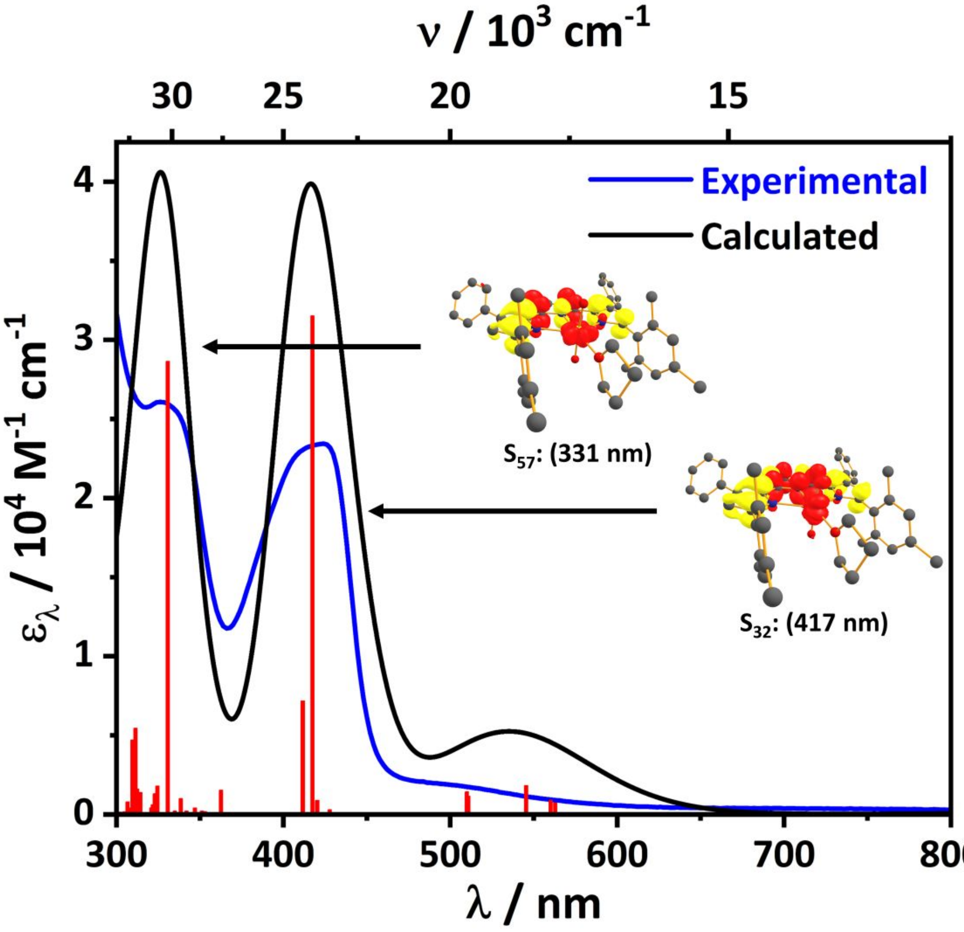

Figure S7. Comparison of experimental (blue) and TD-DFT calculated (black) absorption spectra. The transitions responsible for the bands centered at ca. 410 nm and ca. 330 nm are shown as unrelaxed difference densities (red = gain of electron density, yellow = loss of electron density).

1.1 Input Files, Optimized Coordinates, and Vibrational Frequencies

Geometry Optimization and Frequency Calculation

```
!rks pbe d3bj tightscf zora zora-def2-svp sarc/j normalprint tightopt numfreq
%pal nprocs 20 end
%maxcore 4000
%basis newgto U "sarc-zora-tzvp" end
        newgto O "zora-def2-tzvp" end
        newgto N "zora-def2-tzvp" end
 end
*xyz 0 1
```

TD-DFT Calculation

```
!rks b3lyp tightscf dkh dkh-def2-svp sarc/j normalprint cpcm(thf)
%pal nprocs 20 end
%maxcore 7000
%basis newgto U "sarc-dkh-tzvp" end
        newgto O "dkh-def2-tzvp" end
        newgto N "dkh-def2-tzvp" end
 end
%tddft nroots 100
        tda false
        dosoc true
end
%rel picturechange 2
      finitenuc true
end
*xyz 0 1
```

Optimized S_0 Coordinates

U	-0.08576684524397	-0.10555266713441	0.02410624604434
O	-0.14882829938628	-1.90281365719485	-0.02965854993452
O	-0.02762504283256	1.68367358512263	-0.15544244107975
O	-0.12908015487573	-0.19480152106261	-2.42743319036161
N	-2.22497740650785	0.00466763932157	1.04363792934394
N	-0.04696644788095	0.01549608510938	2.54623729726949
N	2.08250000553964	-0.14364286776361	0.98258328673133
C	-3.44440540088246	0.02197257460654	0.42977264111526
C	-4.44942563507716	0.15338173596238	1.40265224920910
H	-5.52812901199419	0.15813018951884	1.22426693346323
C	-3.81103509548007	0.19678661072375	2.67825211928162
C	-2.41894080184033	0.11740552036619	2.41417156094192
C	-1.22181218430016	0.20206087072837	3.22608564354746
C	-1.20670866919371	0.48495259460921	4.60950319136506
H	-2.14645562141843	0.67218298866957	5.13482727563727
C	0.02142868240211	0.54392092748268	5.27577297297720
H	0.05017799989299	0.78237786673217	6.34607997169033
C	1.21354914500952	0.31078030982942	4.58271229087680
H	2.18229192508781	0.36025573792182	5.08617318765374
C	1.15760543095161	0.04297352250759	3.19762607601311
C	2.31595513155052	-0.16225849561194	2.35082834380595
C	3.71136381012015	-0.28159342057143	2.57960582734031
C	4.31416383835439	-0.30594229663808	1.28542002950026
H	5.38147444617115	-0.41576198236631	1.07531715764774
C	3.28188513107394	-0.22806717043314	0.33577719299100
C	-4.53580053240755	0.25224390137994	3.96148021087889
C	-5.57115983073841	1.19615179995549	4.15572832532844
H	-5.78728174230102	1.91540792774187	3.35596223610458
C	-6.30036171162635	1.23277386525722	5.35302519658153
H	-7.09423332789259	1.97889319703990	5.48339822078016

C	-6.01273950416045	0.32465696830113	6.38493253969330
H	-6.58111180471441	0.35425066295662	7.32255702009392
C	-4.99483151681286	-0.62683974199038	6.20349106185278
H	-4.77211114444048	-1.35168606649117	6.99656283623384
C	-4.26924220730712	-0.66642542480929	5.00459608706982
H	-3.49373977707302	-1.42818506735299	4.85693910103631
C	4.45441571736200	-0.41338442452009	3.84567438233437
C	4.04929047226404	-1.32359077365802	4.85127214532245
H	3.15617314823106	-1.93765551417357	4.68268369764120
C	4.78277365834414	-1.46058349869566	6.03784977358358
H	4.45051675956910	-2.17464632796442	6.80184622413146
C	5.94671041142448	-0.70065817952658	6.24383225719915
H	6.52104357766100	-0.80933525236535	7.17194458714146
C	6.37176808508183	0.19419477045883	5.24830121411992
H	7.27974126458825	0.79178204172934	5.39797303152297
C	5.63477627027960	0.33540420775603	4.06374952904255
H	5.95851415517514	1.04734083273732	3.29434791606726
C	-3.56082342361531	-0.13312893492057	-1.04440712009042
C	-3.69570820661439	-1.43378425040080	-1.60521145980797
C	-3.86797089815352	-1.56120270927001	-2.99555190481322
H	-3.98765282077209	-2.56610754158506	-3.42474906860470
C	-3.90841302501330	-0.43954867455609	-3.84625097034738
C	-3.75290392544467	0.83486332850202	-3.27143798230561
H	-3.78367579694617	1.72285802539881	-3.91852491909808
C	-3.58102754018201	1.01096372559830	-1.88442324108563
C	-3.66287414079612	-2.65348646151082	-0.71723413649902
H	-4.41590696343842	-2.57680370259580	0.08709731293842
H	-2.68223952148672	-2.75442444070191	-0.21703662154186
H	-3.85461674611827	-3.57378645822409	-1.29358748014135
C	-4.12806884904092	-0.60894689265584	-5.33084103691559
H	-3.42390985363699	-1.34242747158990	-5.76576941567309
H	-4.00683007595630	0.34464972296647	-5.87248559340415
H	-5.14749170781694	-0.98307415040608	-5.54139215390985
C	-3.43770357280924	2.39400046189851	-1.29560899528106
H	-4.22025864018426	2.58347046667252	-0.53923885797665
H	-3.51052464721902	3.17060436971763	-2.07535399595905
H	-2.47027046215628	2.51325445641819	-0.77419026555495
C	3.34362017177373	-0.20579589068181	-1.14983118591193
C	3.31969531817857	-1.41968098955320	-1.88587445785031
C	3.42815572639863	-1.36729054543641	-3.28934309517988
H	3.42302707258525	-2.30913577778535	-3.85595782497650
C	3.56604323638727	-0.15001559059013	-3.98078733293527
C	3.57000146196565	1.04206647294266	-3.23141019353436
H	3.67648761928882	2.00456988062239	-3.75160543286569
C	3.45844895176660	1.03889076825976	-1.82883683858215
C	3.19554038729368	-2.74439238341994	-1.17231843197184
H	4.00563407055361	-2.87173382411742	-0.43216821455644
H	3.23663401411484	-3.58652396277481	-1.88333412428402
H	2.24763643897883	-2.81202122050150	-0.60752499514541
C	3.72949046403510	-0.11518527930888	-5.48193872907315
H	4.76804471880423	0.14445849316052	-5.76140179880351
H	3.07469428637748	0.64527314659888	-5.94560083486833
H	3.49953445897265	-1.09213196149943	-5.94063114391669
C	3.46199245416154	2.33389740231578	-1.05413077658298
H	2.48081770202875	2.51313717719392	-0.57639368167930
H	3.68881374667452	3.19143746844843	-1.70928254011596
H	4.20490438529189	2.30507351280855	-0.23773306170175
C	-0.05381518387078	0.97393812607382	-3.31421347761291
H	0.98306621055562	1.35101415613182	-3.26677117573165
H	-0.74834521334500	1.73237362691473	-2.91838964808242
C	-0.42525866016384	0.44957760105476	-4.69646250851482
H	0.05952354816520	1.03015316114254	-5.49963224684127
H	-1.51804432561015	0.50021325155995	-4.83755451869901
C	0.04516873596463	-1.01312096327391	-4.65215167780490
H	1.12899732163628	-1.07702313153491	-4.84673694442803
H	-0.47974926037563	-1.65333464057999	-5.38134774623698

C -0.25162761179878 -1.42852344971367 -3.21603052871756
H -1.28432835684249 -1.79663127175232 -3.08413033790493
H 0.46346757770794 -2.15649081566220 -2.80035942564948

S_0 Vibrational Frequencies

6: 9.52 cm**-1
7: 17.82 cm**-1
8: 22.06 cm**-1
9: 28.67 cm**-1
10: 31.36 cm**-1
11: 33.94 cm**-1
12: 35.67 cm**-1
13: 41.40 cm**-1
14: 47.92 cm**-1
15: 48.78 cm**-1
16: 53.26 cm**-1
17: 55.98 cm**-1
18: 59.69 cm**-1
19: 61.69 cm**-1
20: 62.67 cm**-1
21: 76.04 cm**-1
22: 82.05 cm**-1
23: 87.35 cm**-1
24: 95.34 cm**-1
25: 99.13 cm**-1
26: 102.88 cm**-1
27: 104.30 cm**-1
28: 106.17 cm**-1
29: 113.37 cm**-1
30: 114.73 cm**-1
31: 118.49 cm**-1
32: 123.45 cm**-1
33: 127.94 cm**-1
34: 133.04 cm**-1
35: 139.76 cm**-1
36: 146.45 cm**-1
37: 150.48 cm**-1
38: 158.51 cm**-1
39: 160.72 cm**-1
40: 173.28 cm**-1
41: 175.44 cm**-1
42: 182.39 cm**-1
43: 183.08 cm**-1
44: 192.42 cm**-1
45: 197.47 cm**-1
46: 202.74 cm**-1
47: 204.93 cm**-1
48: 215.03 cm**-1
49: 224.34 cm**-1
50: 232.87 cm**-1
51: 233.71 cm**-1
52: 234.65 cm**-1
53: 250.75 cm**-1
54: 255.08 cm**-1
55: 270.16 cm**-1
56: 272.29 cm**-1
57: 274.24 cm**-1
58: 279.86 cm**-1
59: 290.53 cm**-1
60: 306.88 cm**-1
61: 309.15 cm**-1
62: 312.85 cm**-1
63: 327.96 cm**-1
64: 339.30 cm**-1
65: 386.46 cm**-1
66: 399.39 cm**-1

67: 403.77 cm**-1
68: 404.54 cm**-1
69: 425.33 cm**-1
70: 432.36 cm**-1
71: 433.06 cm**-1
72: 440.52 cm**-1
73: 465.01 cm**-1
74: 489.06 cm**-1
75: 492.25 cm**-1
76: 503.90 cm**-1
77: 506.70 cm**-1
78: 512.18 cm**-1
79: 516.33 cm**-1
80: 541.26 cm**-1
81: 545.25 cm**-1
82: 551.70 cm**-1
83: 560.32 cm**-1
84: 564.38 cm**-1
85: 571.08 cm**-1
86: 571.66 cm**-1
87: 591.98 cm**-1
88: 596.21 cm**-1
89: 606.38 cm**-1
90: 607.64 cm**-1
91: 611.44 cm**-1
92: 618.78 cm**-1
93: 631.80 cm**-1
94: 654.97 cm**-1
95: 673.36 cm**-1
96: 684.46 cm**-1
97: 689.09 cm**-1
98: 690.78 cm**-1
99: 691.73 cm**-1
100: 693.35 cm**-1
101: 698.58 cm**-1
102: 712.59 cm**-1
103: 727.64 cm**-1
104: 736.11 cm**-1
105: 745.04 cm**-1
106: 748.22 cm**-1
107: 754.91 cm**-1
108: 755.54 cm**-1
109: 772.58 cm**-1
110: 790.03 cm**-1
111: 794.19 cm**-1
112: 809.40 cm**-1
113: 812.11 cm**-1
114: 825.56 cm**-1
115: 827.41 cm**-1
116: 827.85 cm**-1
117: 830.63 cm**-1
118: 835.46 cm**-1
119: 837.12 cm**-1
120: 851.58 cm**-1
121: 864.24 cm**-1
122: 865.04 cm**-1
123: 865.26 cm**-1
124: 893.09 cm**-1
125: 899.48 cm**-1
126: 900.73 cm**-1
127: 904.06 cm**-1
128: 921.50 cm**-1
129: 925.58 cm**-1
130: 927.39 cm**-1
131: 928.40 cm**-1
132: 930.72 cm**-1

133: 945.76 cm**-1
134: 946.59 cm**-1
135: 950.42 cm**-1
136: 951.63 cm**-1
137: 958.86 cm**-1
138: 962.93 cm**-1
139: 964.10 cm**-1
140: 965.63 cm**-1
141: 968.07 cm**-1
142: 975.07 cm**-1
143: 979.92 cm**-1
144: 984.73 cm**-1
145: 992.96 cm**-1
146: 996.47 cm**-1
147: 997.62 cm**-1
148: 1003.53 cm**-1
149: 1004.00 cm**-1
150: 1011.91 cm**-1
151: 1012.13 cm**-1
152: 1012.56 cm**-1
153: 1017.58 cm**-1
154: 1018.46 cm**-1
155: 1019.54 cm**-1
156: 1021.97 cm**-1
157: 1028.48 cm**-1
158: 1029.84 cm**-1
159: 1030.66 cm**-1
160: 1031.65 cm**-1
161: 1037.02 cm**-1
162: 1056.40 cm**-1
163: 1067.23 cm**-1
164: 1072.97 cm**-1
165: 1078.72 cm**-1
166: 1084.86 cm**-1
167: 1088.08 cm**-1
168: 1108.77 cm**-1
169: 1123.13 cm**-1
170: 1138.88 cm**-1
171: 1140.28 cm**-1
172: 1145.50 cm**-1
173: 1148.98 cm**-1
174: 1150.61 cm**-1
175: 1158.86 cm**-1
176: 1160.23 cm**-1
177: 1160.71 cm**-1
178: 1161.26 cm**-1
179: 1184.94 cm**-1
180: 1189.24 cm**-1
181: 1219.24 cm**-1
182: 1220.48 cm**-1
183: 1224.78 cm**-1
184: 1226.32 cm**-1
185: 1228.05 cm**-1
186: 1229.23 cm**-1
187: 1273.29 cm**-1
188: 1279.34 cm**-1
189: 1280.45 cm**-1
190: 1289.34 cm**-1
191: 1294.02 cm**-1
192: 1299.92 cm**-1
193: 1300.19 cm**-1
194: 1301.01 cm**-1
195: 1303.71 cm**-1
196: 1318.88 cm**-1
197: 1328.91 cm**-1
198: 1333.15 cm**-1

199: 1344.23 cm**-1
200: 1345.46 cm**-1
201: 1346.70 cm**-1
202: 1353.55 cm**-1
203: 1356.44 cm**-1
204: 1361.67 cm**-1
205: 1362.47 cm**-1
206: 1364.05 cm**-1
207: 1365.67 cm**-1
208: 1365.95 cm**-1
209: 1367.46 cm**-1
210: 1369.54 cm**-1
211: 1389.88 cm**-1
212: 1400.66 cm**-1
213: 1401.82 cm**-1
214: 1403.31 cm**-1
215: 1407.70 cm**-1
216: 1419.86 cm**-1
217: 1424.77 cm**-1
218: 1424.78 cm**-1
219: 1426.04 cm**-1
220: 1427.29 cm**-1
221: 1427.74 cm**-1
222: 1428.56 cm**-1
223: 1428.77 cm**-1
224: 1435.41 cm**-1
225: 1437.78 cm**-1
226: 1438.34 cm**-1
227: 1439.73 cm**-1
228: 1440.18 cm**-1
229: 1444.86 cm**-1
230: 1450.08 cm**-1
231: 1456.66 cm**-1
232: 1458.99 cm**-1
233: 1459.79 cm**-1
234: 1461.34 cm**-1
235: 1465.77 cm**-1
236: 1481.68 cm**-1
237: 1490.20 cm**-1
238: 1519.09 cm**-1
239: 1520.40 cm**-1
240: 1549.50 cm**-1
241: 1549.66 cm**-1
242: 1560.10 cm**-1
243: 1577.97 cm**-1
244: 1578.27 cm**-1
245: 1587.87 cm**-1
246: 1588.42 cm**-1
247: 1605.95 cm**-1
248: 1619.95 cm**-1
249: 1620.27 cm**-1
250: 1621.83 cm**-1
251: 1622.31 cm**-1
252: 2981.79 cm**-1
253: 2981.83 cm**-1
254: 2988.84 cm**-1
255: 2989.00 cm**-1
256: 2989.63 cm**-1
257: 2991.46 cm**-1
258: 3016.07 cm**-1
259: 3019.30 cm**-1
260: 3029.10 cm**-1
261: 3031.80 cm**-1
262: 3052.32 cm**-1
263: 3053.01 cm**-1
264: 3064.32 cm**-1

265:	3065.13 cm**-1
266:	3065.22 cm**-1
267:	3067.85 cm**-1
268:	3080.61 cm**-1
269:	3080.96 cm**-1
270:	3081.89 cm**-1
271:	3086.81 cm**-1
272:	3086.98 cm**-1
273:	3087.08 cm**-1
274:	3089.49 cm**-1
275:	3089.97 cm**-1
276:	3093.19 cm**-1
277:	3098.11 cm**-1
278:	3098.25 cm**-1
279:	3099.25 cm**-1
280:	3100.01 cm**-1
281:	3100.07 cm**-1
282:	3116.57 cm**-1
283:	3117.15 cm**-1
284:	3122.16 cm**-1
285:	3123.02 cm**-1
286:	3131.19 cm**-1
287:	3132.20 cm**-1
288:	3133.14 cm**-1
289:	3135.81 cm**-1
290:	3137.06 cm**-1
291:	3144.64 cm**-1
292:	3145.22 cm**-1
293:	3174.55 cm**-1
294:	3176.54 cm**-1
295:	3176.79 cm**-1
296:	3177.22 cm**-1

1.2 References

(1) Neese, F. “The ORCA Program System.” *Wiley Interdisciplinary Reviews: Computational Molecular Science* **2012**, *2*, 73–78.

(2) Neese, F. “Software Update: The ORCA Program System, Version 4.0.” *Wiley Interdisciplinary Reviews: Computational Molecular Science* **2018**, *8*.

(3) Perdew, J. P.; Burke, K.; Ernzerhof, M. “Generalized Gradient Approximation Made Simple.” *Physical Review Letters* **1996**, *77*, 3865–3868.

(4) Vahtras, O.; Almlöf, J.; Feyereisen, M. W. “Integral Approximations for LCAO-SCF Calculations.” *Chemical Physics Letters* **1993**, *213*, 514–518.

(5) Neese, F. “An Improvement of the Resolution of the Identity Approximation for the Formation of the Coulomb Matrix.” *Journal of Computational Chemistry* **2003**, *24*, 1740–1747.

(6) Van Wüllen, C. “Molecular Density Functional Calculations in the Regular Relativistic Approximation: Method, Application to Coinage Metal Diatomics, Hydrides, Fluorides and Chlorides, and Comparison with First-Order Relativistic Calculations.” *Journal of Chemical Physics* **1998**, *109*, 392–399.

(7) Weigend, F.; Ahlrichs, R. “Balanced Basis Sets of Split Valence, Triple Zeta Valence and Quadruple Zeta Valence Quality for H to Rn: Design and Assessment of Accuracy.” *Physical Chemistry Chemical Physics* **2005**, *7*, 3297–3305.

(8) Pantazis, D. A.; Neese, F. “All-Electron Scalar Relativistic Basis Sets for the Actinides.” *Journal of Chemical Theory and Computation* **2011**, *7*, 677–684.

(9) Grimme, S.; Antony, J.; Ehrlich, S.; Krieg, H. “A Consistent and Accurate Ab Initio Parametrization of Density Functional Dispersion Correction (DFT-D) for the 94 Elements H-Pu.” *Journal of Chemical Physics* **2010**, *132*, 180901.

(10) Grimme, S.; Ehrlich, S.; Goerigk, L. “Effect of the Damping Function in Dispersion Corrected Density Functional Theory.” *Journal of Computational Chemistry* **2011**, *32*, 1456–1465.

(11) Lee, C.; Yang, W.; Parr, R. G. “Development of the Colle-Salvetti Correlation-Energy Formula into a Functional of the Electron Density.” *Physical Review B* **1988**, *37*, 785–789.

(12) Neese, F.; Wennmohs, F.; Hansen, A.; Becker, U. “Efficient, Approximate and Parallel Hartree-Fock and Hybrid DFT Calculations. A ‘chain-of-Spheres’ Algorithm for the Hartree-Fock Exchange.” *Chemical Physics* **2009**, *356*, 98–109.

(13) Helmich-Paris, B.; de Souza, B.; Neese, F.; Izsák, R. “An Improved Chain of Spheres for Exchange Algorithm.” *Journal of Chemical Physics* **2021**, *155*, 104109.

(14) Neese, F. “Efficient and Accurate Approximations to the Molecular Spin-Orbit Coupling Operator and Their Use in Molecular g-Tensor Calculations.” *Journal of Chemical Physics* **2005**, *122*.

(15) Garcia-Ratés, M.; Neese, F. “Effect of the Solute Cavity on the Solvation Energy and Its Derivatives within the Framework of the Gaussian Charge Scheme.” *Journal of Computational Chemistry* **2020**, *41*, 922–939.
